# Fish Species Sensitivity Ranking Depends on Pesticide Exposure Profiles

**DOI:** 10.1002/etc.5348

**Published:** 2022-06-06

**Authors:** Dirk Nickisch Born Gericke, Björn Christian Rall, Alexander Singer, Roman Ashauer

**Affiliations:** ^1^ RIFCON Hirschberg Germany; ^2^ Syngenta Crop Protection Basel Switzerland; ^3^ Department of Environment and Geography University of York York UK

**Keywords:** Toxicokinetic–toxicodynamic modeling, General unified threshold models of survival, Median lethal profile, Species sensitivity distribution, Fish, Ecotoxicology

## Abstract

In the regulatory environmental risk assessment of plant protection products, the exposure tested in standard toxicity tests assumes simple exposure dynamics, such as constant exposure at the first stage of testing. However, environmental exposure can be highly dynamic. A species response to exposure is governed by toxicokinetics (TK) and toxicodynamics (TD). Therefore, it can be expected that the sensitivity of a species to a substance is dependent on the interplay of TKTD processes with the dynamics of the exposure. We investigated whether exposure dynamics affects species sensitivity of five fish species and if their sensitivity rankings differ among exposure profiles. We analyzed individual survival under projected surface water exposure to benzovindiflupyr. For this purpose, we calibrated compound‐ and species‐specific reduced general unified threshold models of survival (GUTS‐RED) models from standard laboratory toxicity data with the assumptions of stochastic death and individual tolerance. Using the calibrated models, we generated species sensitivity distributions based on median lethal profile multiplication factors for three characteristic exposure profiles. The analysis was performed using different GUTS‐RED implementations: openGUTS (MATLAB® and Windows® versions) and the R package morse. The sensitivity rankings of the fish species changed as a function of exposure profile. For a multiple‐peak scenario, rainbow trout was the most sensitive species. For a single peak followed by a slow concentration decline the most sensitive species was the fathead minnow (GUTS‐RED‐stochastic death) or the common carp (GUTS‐RED‐individual tolerance). Our results suggest that a single most sensitive species cannot be defined for all situations, all exposure profiles, and both GUTS‐RED variants. *Environ Toxicol Chem* 2022;41:1732–1741. © 2022 Syngenta. *Environmental Toxicology and Chemistry* published by Wiley Periodicals LLC on behalf of SETAC.

## INTRODUCTION

For environmental risk assessment (ERA) of plant protection products (PPPs), the toxic risk for a species group is statistically extrapolated from the estimated risks of a sample of representive species. The European Food Safety Authority (EFSA) Panel on Plant Protection Products and Their Residues (PPR; PPR et al., 2018) suggests extrapolation metrics that are based on aggregating the distribution of the species' sensitivity (PPR, 2013). These include (1) the highest estimated risk, assuming that the risk to the most sensitive species conservatively represents the risk to the group, and (2) the species sensitivity distribution (SSD; Aldenberg et al., 2002). The SSD assumes that the tested species are a representative group in terms of risk from which the risk to the 5% most sensitive species can be estimated. Thus, these metrics allow extrapolation of the species group risk from selected representative species for which the risk was determined in standard toxicity tests.

In regulatory ERA, risk is translated into the level of exposure that is deemed acceptable to an affected species. The acceptable exposure is then compared with the predicted environmental concentration (PEC). Environmental exposure can be highly dynamic. In contrast, the exposure tested in standard toxicity tests assumes simple exposure dynamics, such as constant exposure (Organisation for Economic Cooperation and Development, 1992), at the first‐tier level of testing. These simple exposure dynamics hardly represent variable environmental exposure dynamics, and it is an open question how to best relate exposures determined from laboratory studies to PEC values.

To account for this discrepancy in comparing acceptable and environmental exposure, the standard regulatory risk assessment defines safety factors. These safety factors are assumed to indicate how much the critical exposure determined from toxicity tests must exceed the maximal PEC to be regulatorily acceptable. This approach neglects the dynamics of environmental exposure.

Recent toxicokinetic–toxicodynamic (TKTD) modeling approaches provide refined ecotoxicological assessment of the time‐variable PECs (e.g., time series of concentrations in water bodies). Such models are calibrated to standard toxicity tests. Using the calibrated models, expected toxicity‐related effects can be predicted based on the full TKTD dynamics. Hence, TKTD models combine the information contained in standard toxicity tests and time‐variable exposure profiles. The EFSA details the potential application of TKTD modeling in the European regulatory ERA for aquatic species (PPR et al., 2018). To estimate the risk of PPP to species groups with TKTD models, the EFSA suggests extending the metrics that are applied in the standard risk assessment to extrapolate the risks from the representative species to their group. Yet, because of different abilities of TKTD models compared with standard ecotoxicological statistical analyses of toxicity tests, the extension is not straightforward. For example, TKTD models provide estimates of prediction uncertainty, which can be incorporated into SSD assessments (Charles et al., 2021).

We investigated whether the consideration of dynamics in PECs affects the estimates of species sensitivities and thus the risk assessment of species groups. We hypothesized that an impact would be expected because toxicological mechanisms differ among species, such as how a toxicant is taken up and distributed in an individual's body (TK) and how the toxicant affects the species performance (TD; Ashauer & Jager, 2018; Gergs et al., 2019).

We analyzed the lethal risk to fish in a realistic, dynamic environmental exposure scenario using benzovindiflupyr. For this purpose, we estimated the species‐specific risk to five fish species using the general unified threshold models of survival (GUTS; Jager et al., 2011). This is a TKTD modeling approach for the assessment of lethal effects. For each of the species analyzed, GUTS models are calibrated based on observed survival in standard toxicity tests. Using the calibrated models, we predicted the species‐specific mortality risk under three realistic PEC scenarios, that is, three different exposure concentration time series. For each exposure scenario, we ranked the species according to their predicted risk and assessed both the most sensitive species and the SSD. Robustness of results was ensured by repeating the analysis with three software implementations of the GUTS framework.

## MATERIALS AND METHODS

In the present study we addressed (1) if different exposure profiles and/or reduced GUTS (GUTS‐RED) variations lead to differences in determining the most sensitive species, and (2) if different software solutions lead to different calibration results and different species sensitivities.

We used the data from Ashauer et al. (2013) that consist of five acute toxicity tests and one early–life stage (ELS) toxicity test on five fish species exposed to benzovindiflupyr. The five fish species are common carp (*Cyprinus carpio*), fathead minnow (*Pimephales promelas*), sheepshead minnow (*Cyprinodon variegatus*), rainbow trout (*Oncorhynchus mykiss*), and bluegill (*Lepomis macrochirus*).

Each exposure scenario used for calibration consisted of five concentrations in addition to a control and a solvent control. For all acute studies, the control and solvent control showed similar survival and therefore were pooled by summing the number of survivors for each time step.

The mean measured concentration of benzovindiflupyr in the water deviated by >20% from the nominal value for *O. mykiss* and *Cyprinodon variegatus*. To comply with recommendations (PPR et al., 2018) and to apply a consistent methodology across studies, mean measured concentrations were used in GUTS calibrations for all species. In all control measurements, concentrations did not exceed the limit of quantification. Therefore, concentrations in control treatments could be assumed to be zero.

For the ELS study (*P. promelas*), reported survival was censored in several steps prior to applying it for GUTS modeling: (1) Because the GUTS‐RED approach cannot account for changes in life stages, we ignore the prehatch phase in model calibration. However, exposed eggs might carry over potential effects to the fry stage. Therefore, empirically measured fry survival might be decreased as a result of egg exposure. Calibrating GUTS to such decreased fry survival leads to a parametrization of the model, which we expect to lead to decreased survival in its model projections. (2) The reported number of survivors did not monotonically decline over time. Therefore, for each replicate, the observed number of survivors was transformed by limiting the maximum number of observed survivors at each time step to the value in the previous time step. This transformation assumes that individuals were erroneously reported dead, potentially because of misinterpretation of the observed behavior or appearance. The correction preserves the total number of reported individuals at day 28 (the last day of the posthatch phase). (3) Next, for each treatment level, replicates were pooled by summing the number of survivors. (4) Finally, control and solvent control were pooled by summing the number of survivors, in accordance with the procedure for the acute data. Prior to pooling, the similarity of the number of survivors in the control and solvent control was statistically tested using statistical software R, Ver 3.6.3 (R Foundation for Statistical Computing, 2020). No significant difference in the number of survivors was observed throughout the time series (Pr(>|*z*|) = 0.13) using a Cox proportional hazard model (Andersen & Gill, 1982; Therneau, 2020; Therneau & Grambsch, 2000). In addition, no significant difference between the number of survivors in the two controls was found at the end of the ELS study using a two‐sided *t* test (*p* = 0.45).

### The model

The GUTS (Jager et al., 2011) mechanistic modeling approach projects individual survival under temporally varying exposure profiles. The conceptual background of GUTS has been described in detail and is agreed on by the ERA modeling community (Jager & Ashauer, 2018). In particular, the two reduced versions, with the assumptions of stochastic death and individual tolerance, GUTS‐RED‐stochastic death and GUTS‐RED‐individual tolerance, are suggested as models to analyze the lethal effects of PPPs on test species in aquatic risk assessments (PPR et al., 2018). For details, see Supporting Information.

### GUTS software implementations

We applied three different GUTS softwares, including two openGUTS implementations (MATLAB® Ver 1.1 and Windows® Ver 1.1) and the statistical software R (R Foundation for Statistical Computing, 2020) package “morse” (Ver 3.2.5; Baudrot et al., 2019). We chose these software implementations because openGUTS and morse are commonly used and well documented (Baudrot & Charles, 2019, 2021; Charles et al., 2021; Jager, 2019, 2021a). Moreover, these implementations use the two main approaches for parameter fitting, frequentist (openGUTS) and Bayesian (morse), described in the EFSA opinion (PPR et al., 2018). The implementation openGUTS MATLAB has the disadvantage that MATLAB is a commercial software. Therefore, we also used the new user‐friendly openGUTS standalone Windows Ver 1.1, which was not part of the ring test of several GUTS‐RED implementations (Jager & Ashauer, 2018). We tested these three software versions to ensure that the findings are robust to implementation uncertainty and to test the new openGUTS standalone Windows Ver 1.1 on a case study using actual fish data.

### Parameter estimation

Calibration methods implemented in the software implementations openGUTS and morse were used for parameter fitting.

To estimate model parameter values from empirical survival data, the openGUTS implementations use a frequentist approach (Jager, 2019, 2021a). The morse package applies the Monte Carlo Markov chain to sample from the joint posterior of the parameter values under a Bayesian framework (Baudrot et al., 2019).

The openGUTS implementations offer two options for dealing with estimation of background mortality: fitting it to the observed survival in the control only or fitting it together with the other parameters using the entire data set from one experiment. The morse version used for calibrations in the present study (Ver 3.2.5) does not yet offer the first option, so all parameters were fitted together to increase model comparability. In the latest morse version, which was not published at the time this part of the study was conducted, a fixed value can be set as background mortality.

The available ELS toxicity test data did not conform to recommendations for GUTS validation data (PPR et al., 2018, Section 4.1.4.5). In contrast to Ashauer et al. (2013), where the ELS test was used for validation, we decided to use these data for model calibration, together with the acute toxicity data. Using the ELS test for model calibration is a compromise that, on the one hand, follows the EFSA suggestion to use all available suitable data (PPR et al., 2018, Checklist 7d) and aim to maximize the information used. On the other hand, the ELS test design violates GUTS assumptions, for example, that an organism must not change fundamentally during the test. Using ELS data for calibration reduced parameter confidence intervals and led to small changes in the point estimates of the toxicity parameters (see Supporting Information, Figure S8).

In accordance with ethical recommendations to limit vertebrate toxicity tests, no additional studies were conducted for GUTS validation. Such studies are not always mandatory (PPR et al., 2018, Section 7.7.2) considering the quality of the calibrated GUTS models and the multitude of test species. The reduction of vertebrate tests is important (see European Union regulation 283/2013 or EFSA 2021).

### Lethal profile estimation (model use, predictions)

A lethal profile (LP*x*) is the factor with which an exposure profile must be multiplied to predict *x*% mortality with GUTS (Ashauer et al., 2013; Baudrot et al., 2019). We calculated LP50 values with the calibrated GUTS‐RED models (stochastic death and individual tolerance), applying the inbuilt LP*x* calculation routines of the implementations. For morse, we used the newly available Ver 3.3.1 for this purpose, which predicts LP50 more efficiently and reliably than previous versions.

**Figure 1:**
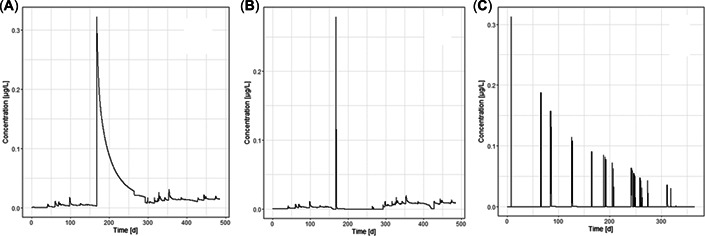
Representative surface water exposure profiles, estimated with the FOCUS SWASH 5.3 surface water models (PRZM 4.3.1, MACRO 5.5.4, and TOXSWA 4.4.3), were used to calculate concentrations in surface waters. d = days.

We predicted LP50 for three characteristic exposure profiles. The exposure profiles were estimated with the FOCUS SWASH 5.3 surface water models (PRZM 4.3.1, MACRO 5.5.4, and TOXSWA 5.5.3), following the FOCUS surface water scenario working group guidelines (FOCUS, 2001). As such, they represent realistic worst‐case simulations for a hypothetical use of benzovindiflupyr in Europe. The hourly resolved exposure concentrations in surface waters were extracted from the TOXSWA outfiles (parameter ConLiqWatLay [grams per cubic meter]) and converted to exposure units (micrograms per liter) to comply with the units of the calibrated GUTS‐RED models. Extraction and unit conversion were performed within the R framework (R Foundation for Statistical Computing, 2020). The exposure profiles (here called *A*, *B*, and *C*) represent different types of typical dynamic exposure patterns (Figure 1).

### SSD estimation

The SSD approach aims at estimating the risk of a substance for a group of species (Aldenberg et al., 2002), in the present study fish; SSDs are cumulative probability distributions that estimate the percentage of species of the group that are lethally affected by a given concentration of a chemical (Aldenberg et al., 2002). The concentration that affects 5% of species is referred to as the *5% hazard concentration* (PPR 2013, Section 8.4). The LP50 for 5% of species was defined as the 5% hazard profiles (HP5; PPR et al., 2018, Chapter 3.2). Thus, HP5 is the factor with which an exposure profile must be multiplied such that for 5% of species mortality is 50% and for 95% of species mortality is <50%.

The R package ssdtools (Thorley & Schwarz, 2018) provides the functions needed to fit SSDs using maximum likelihood and model averaging. Hazard profile values (HP5) for each exposure profile were calculated based on LP50 values (morse, median; openGUTS, maximum likelihood) across species.

## RESULTS

### Parameter estimates

**Figure 2:**
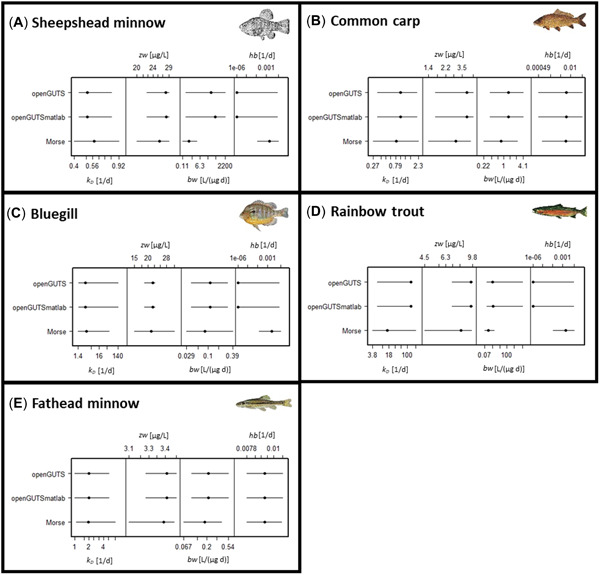
Model comparison for reduced general unified threshold models of survival of fathead minnow (GUTS‐RED‐stochastic death [SD]) across five different fish species: (**A**) sheepshead minnow, (**B**) common carp, (**C**) bluegill, (**D**) rainbow trout, and (**E**) fathead minnow (calibration was performed with acute and early life stage test). Estimated parameters for GUTS‐RED‐SD are dominant rate constant, threshold for effects, killing rate constant, and background mortality. For openGUTS versions, the best fit (dot) and 95% confidence interval (horizontal line) are displayed; for morse, the median (dot) and 95% credible intervals (horizontal line) of the posterior distributions are displayed. *zw* = threshold for effects; *hb* = background mortality; *k*_D_ = dominant rate constant; *bw* = killing rate constant.

**Figure 3:**
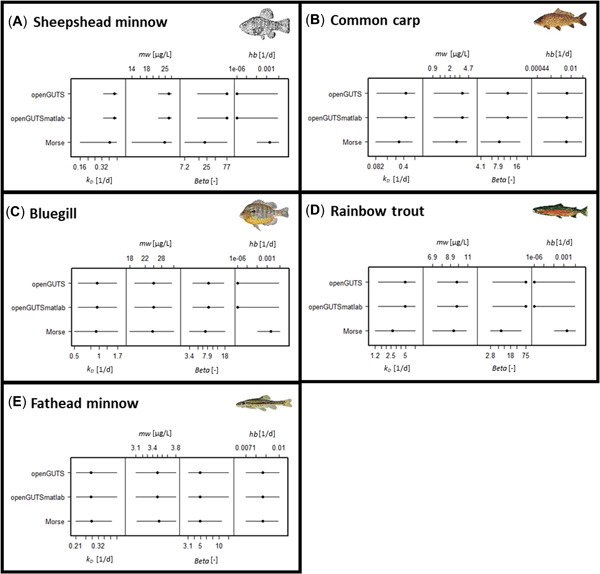
Model comparison for reduced general unified threshold models of survival of common carp (GUTS‐RED‐individual tolerance [IT]) across five different fish species: (**A**) sheepshead minnow, (**B**) common carp, (**C**) bluegill, (**D**) rainbow trout, and (**E**) fathead minnow (calibration was performed with acute and early life stage test). Estimated parameters for GUTS‐RED‐IT are dominant rate constant, median of the threshold distribution, threshold distribution shape parameter, and background mortality. For openGUTS versions, the best fit (dot) and 95% confidence interval (horizontal line) are displayed; for morse, the median (dot) and 95% credible intervals (horizontal line) of the posterior distributions are displayed. *mw* = median of the threshold distribution; *hb* = background mortality; *k*_D_ = dominant rate constant; *beta* = threshold distribution shape parameter.

The three GUTS‐RED software implementations provided generally similar parameter estimates (Figures 2 and 3; Supporting Information, Tables S1–S6) for the parameter dominant rate constant (*k*_D_), the GUTS‐RED‐stochastic death parameters effect threshold, the killing rate (*bw*), as well as the GUTS‐RED‐individual tolerance parameters threshold distribution median and shape. Parameters estimated with morse differ from openGUTS implementations, but median estimates are within the confidence intervals of the openGUTS implementations. As expected, both openGUTS versions calibrated parameters and uncertainities in the same range. One bigger deviation was observed for the killing rate (*bw*) for GUTS‐RED‐stochastic death and species sheepshead minnow (see Figure 2A). The MATLAB version estimated a higher best‐fit value than the standalone version. However, the killing rate is not sensitive in this case, and uncertainties ranged widely.

The visual assessment of the GUTS‐RED simulations was acceptable, and all quantitative performance criteria suggested by PPR et al. (2018) were fulfilled (see Supporting Information, Tables S1–S6).

Calibrated median parameter values from the morse software were within the 95% uncertainty bounds of best‐fit values estimated using the openGUTS versions, with a few exceptions. Larger discrepancies were found when a parameter value was not informed enough by the survival data. In these cases, the estimates from the openGUTS implementations reached the edge of the calibration search space. In contrast, for the morse implementation automatically chosen priors prevented an escape of the calibration algorithm. For three of five species (sheepshead minnow, bluegill, and rainbow trout) openGUTS implementations reached the edge of the calibration search space for the parameter background mortality, and a low value was estimated (see Figures 2 and 3). Lower values were estimated with morse for the killing rate (*bw*) for all species compared with the estimates with openGUTS, which likely is related to the partially informative prior distribution peaking 1–2 orders of magnitude lower than the posterior distribution. We note that also prior distributions for the dominant rate constant (*k*_D_) peaked lower than the posterior distributions, yet the morse estimates coincided with openGUTS estimates (see morse results in Supporting Information). Therefore, it can be concluded that the toxicity data were more informative for the parameter *k*_D_ than for the parameter *bw*.

### LP50 predictions

**Figure 4:**
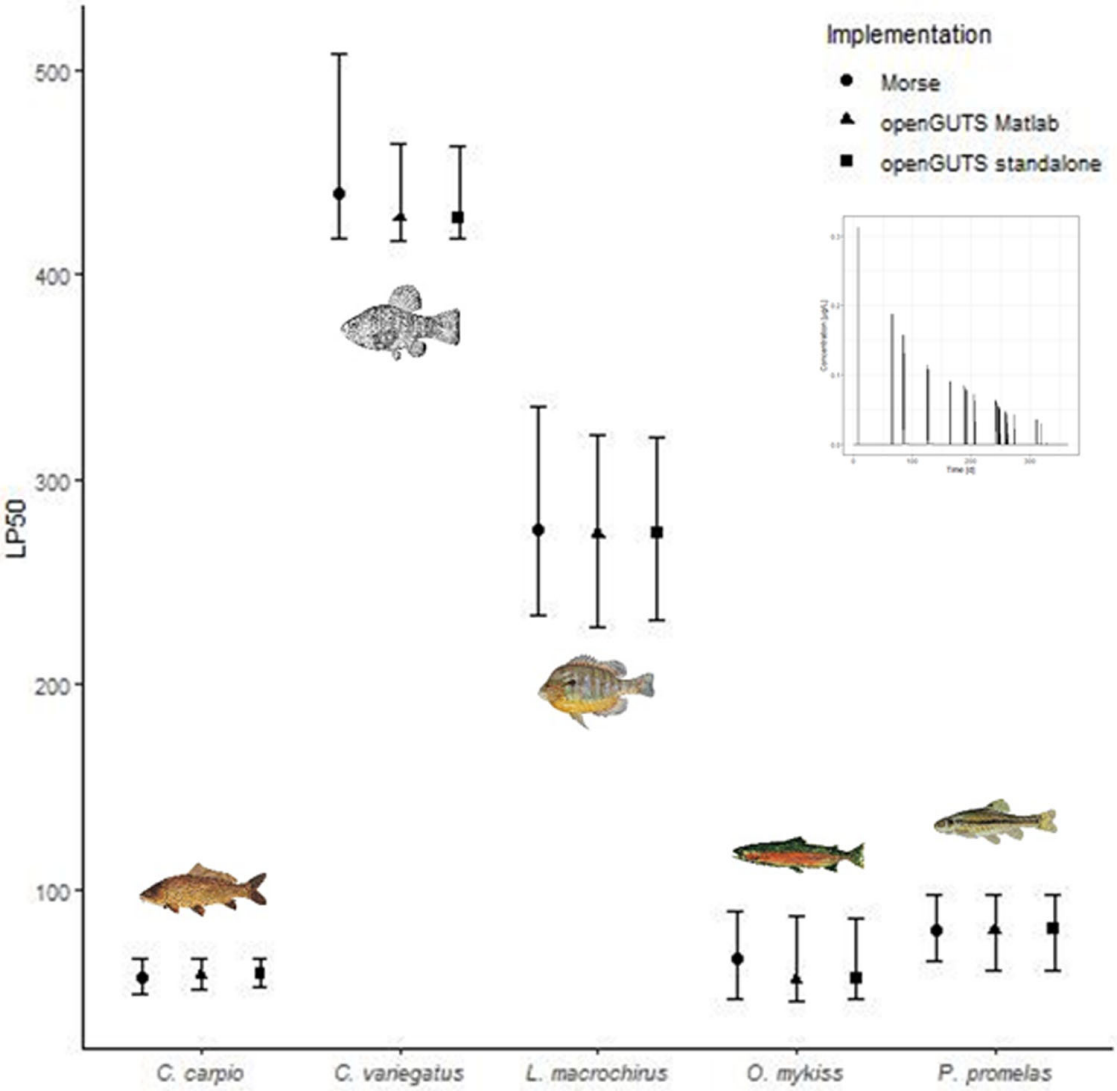
Median lethal profile comparison for reduced general unified threshold models of survival of common carp (GUTS‐RED‐individual tolerance [IT]) implementations (circle = morse, triangle = openGUTS MATLAB®, square = openGUTS standalone) across five different fish species: sheepshead minnow, common carp, bluegill, rainbow trout, and fathead minnow (calibration was performed with acute and early life stage test) for exposure Scenario C. For openGUTS versions, the best fit (dot) and 95% confidence interval (vertical line) are displayed; for morse, the median (dot) and 95% credible intervals (vertical line) of the posterior distributions are displayed. LP50 = median lethal profile.

For all scenarios LP50 values ranged from 14 to 440 (see Figure 4; Supporting Information, S1–S5). The three GUTS‐RED software implementations provided comparable LP50 predictions. The differences of the software implementations in LP50 values were generally within the 95% uncertainty limits. The LP50 values estimated with openGUTS implementations were in some cases slightly higher than those estimated by morse, but confidence intervals were overlapping.

### Estimation of hazard concentrations (SSD)

**Figure 5:**
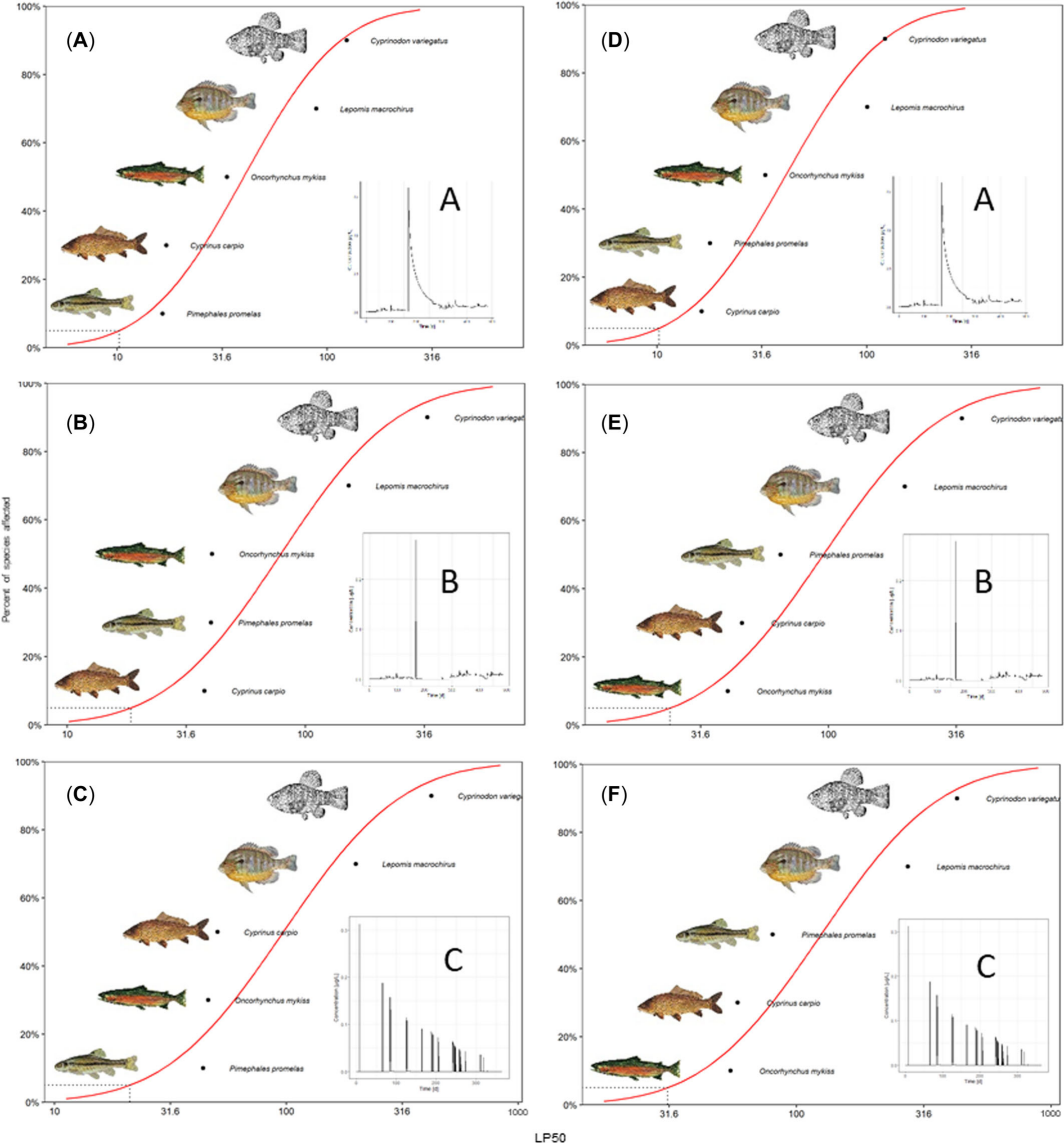
Species sensitivity distributions (SSDs; red line) are cumulative probability distributions that estimate the percentage of species that are affected by a given concentration of a chemical. The concentration that affects 5% of the species is referred to as the 5% hazard concentration (black dashed line). The SSD was built on the median lethal profile estimated with reduced general unified threshold models of survival (GUTS‐RED; openGUTS standalone implementation is shown). Calibration for *Pimephales promelas* was performed using the acute and early life stage test. (**A–C**) Results for fathead minnow GUTS‐RED‐stochastic death (SD); (**D–F**) results for common carp GUTS‐RED‐IT. Insets (**A–C**) describe exposure profiles (shown in Figure 1). LP50 = median lethal profile.

Estimated HP5 values for our test exposure profiles ranged from 9 to 33 (see Supporting Information, Table S7). Sheepshead minnow and bluegill are the least sensitive species, and corresponding LP50 values were in the range of 100 or higher, although bluegill was more sensitive than sheepshead minnow. The sensitivity ranking of these two species was the same in all situations and for all GUTS implementations. The three other species, rainbow trout, fathead minnow, and common carp, were more sensitive than the previously described species. Remarkably, their sensitivity ranking changed among exposure profiles and models. For instance, for a multiple‐peak scenario (Scenario C, Figure 1) the rainbow trout was the most sensitive species in all GUTS implementations, for both GUTS‐RED‐stochastic death and GUTS‐RED‐individual tolerance (see Figure 5C,F). However, for a single peak followed by a slow concentration decline (Scenario A, Figure 1) the most sensitive species was the fathead minnow (GUTS‐RED‐stochastic death; see Figure 5A) or the common carp (GUTS‐RED‐individual tolerance; see Figure 5D). The change in sensitivity rankings as a function of exposure data was also observed in simulation runs with morse, and the order of fish species sometimes differed from the other implementations (e.g., Supporting Information, Figure S7f).

## DISCUSSION

The GUTS‐RED models were used to assess the potential risk of time‐varying exposure profiles on fish species. Our results suggest that choosing a single most sensitive species for all situations, all exposure profiles, and both GUTS‐RED variants (stochastic death and individual tolerance) is not supported by the evidence in the present study. Instead, for each profile at least two species were similarly sensitive. While *Cyprinus carpio* and *P. promelas* were among the most sensitive species for all profiles, *O. mykiss* was less sensitive in Scenario A. Considering the LP50 estimates, we found a tendency that the ranking of species changed, at least if we focused on median estimates as is often done in the risk assessment of PPPs.

We expect that changing species sensitivity rankings across exposure profiles are related to species‐specific differences in TK and TD. The dominant rate constant (*k*_D_) in the GUTS‐RED models is a lumped parameter reflecting both TK and damage dynamics. Its value is dominated by whichever process is slower. Our results show that these processes ranged from slow (*P. promelas*; *k*_D_ = 0.28 day^−1^) to fast (*O. mykiss*; *k*_D_ = 144 day^–1^). Fast TK means that the internal concentration of the toxicant that ultimately exerts a toxic effect closely follows the exposure dynamics. In contrast, slow TK implies that the internal concentration “averages” the external exposure over time, leading to a dampening of exposure peaks and internal concentrations that can be accumulated over longer times. Similar interpretations can be made for fast and slow damage dynamics. Fast damage dynamics means that the damage closely follows the internal concentration of the toxicant. Slow damage dynamics implies dampening of internal concentration peaks and damage accumulation over longer times. Therefore, the risk of a single short spike affecting an individual is higher for *O. mykiss* than for *P. promelas*. On the other hand, exposure over long periods is potentially more critical for *P. promelas*. We saw that *O. mykiss* was less sensitive to Scenario A, a peak with a long tailing, than to Scenarios B and C, one or more short and sharp exposure peaks.

Our LP50 predictions were robust among software implementations for this case study. If, in a few cases, predictions from the different softwares diverged, the discrepancy could be attributed to weak information content of the experimental data that impeded a reliable fit of parameters. Weak information is also often responsible for different results in software implementation using different algorithms (Jager, 2021b). For this reason, the consideration of uncertainties and their incorporation into an ERA are of particular importance (Charles et al., 2021). The similarity of model calibrations and predictions indicates that their different statistical approaches similarly capture the information contained in calibration data. Therefore, critical to the reliability of GUTS predictions is the quality of calibration data rather than the technicalities of model implementations. Our analysis also demonstrates the robustness of the estimation of an HP5 endpoint to using different software implementations for the SSD inputs.

Of the five species the three most sensitive show rather similar sensitivity. This means for the SSD that all fish species in the 10th to 50th percentiles show similar effects, and such a close similarity of species sensitivity cannot be well represented by an SSD fit (see, e.g., Figure 5B,C). Uncertainties of the SSD fit may result in differences in HP5 values among the implementations. The TKTD‐SSD approach or the estimation of the HP5 values is not explained in detail in either the current aquatic guideline (PPR, 2013) or the TKTD opinion (PPR et al., 2018). Charles et al. (2021) recently compared different approaches to calculate SSDs in a case study using data from nontarget plants. Notably, the number of species is usually higher in nontarget plant risk assessments (≥10 species) compared with fish risk assessments (≥5 species). Charles et al. (2021) focused on how the uncertainty of species sensitivity estimates influences the SSD, something that is not normally calculated in regulatory risk assessments. Similarly, in the future the uncertainty from species sensitivity estimates (e.g., LP50) could be propagated to SSD endpoints (e.g., HP5 values) for fish for a more robust sensitivity assessment. However, defining the best method for the fit of an SSD and the estimation of an HP5 was not the focus of the present study.

For species *P. promelas*, we used results from an ELS test for model calibration. In ELS studies, animals grow continously and develop from yolk‐feeding to free‐feeding larvae. In principal, such physical developments cannot be covered by the GUTS‐RED approach because often the toxic response of organisms differs among life stages. Therefore, generally, ELS test data are not modeled with GUTS. In our case, there was a very sensitive phase with increased mortality on day 6. However, because the mortality affected control and treatment levels, this mortality is likely not toxicant‐related; and consequently, GUTS calibration attributed it to background mortality (see, e.g., openGUTS standalone reports in the Supporting Information). An indication could not be found that the toxic response of *P. promelas* changed during the ELS test. Nevertheless, a priori, it is questionable whether the ELS test might bias model calibrations (life stages differed from that in the acute test, and egg stage exposure was ignored) or if the additional ELS information improved the calibrations. Therefore, we repeated the calibration ignoring the data from the ELS test. We found that parameter uncertainties shrank for most of the toxicity‐related parameters when the ELS test data were considered (for details, see Supporting Information, Figure S9). Similarly, LP50 uncertainty was reduced when considering ELS and acute test data in GUTS calibrations (compare Figure 4 and Supporting Information, Figures S1–S5, S9–S14), and the picture of species sensitivities did not change (Supporting Information, Figure S15). The reduction of uncertainties suggests that in our case the incorporation of ELS data can improve GUTS calibration, even though basic model assumptions are violated. This is helpful in the context of reducing animal testing because available data can efficiently be used, but it should be discussed case by case whether violating the model assumptions can be accepted. Further research is needed to generally assess when ELS test data might be suitable for GUTS calibration.

Given that a single most sensitive species could not be identified in this analysis and that the species‐sensitivity ranking order might change among exposure profiles, it is unlikely that a species showing highest sensitivity in a standard acute test with simplistic exposure dynamics can be robustly considered the most sensitive species in all potential environmental exposure dynamics. This is consistent with the finding by Ashauer et al. (2013) that, for the same species and similar exposure profiles as in the present study, the species sensitivity predicted by the GUTS model depends on the exposure profile. The finding that species‐sensitivity ranking changes depending on the exposure profile has also been made for different species of frog and fish exposed to malathion (Ashauer et al., 2016). In the present study we find that even if the ranking of species in the SSD changes, the risk assessment endpoint derived from the SSD method, that is, the HP5, does not change. Therefore, SSD‐based estimates of HP5 could be considered more robust than approaches based on identifying the most sensitive species for ERA.

## Supporting Information

The Supporting Information is available on the Wiley Online Library at https://doi.org/10.1002/etc.5348.

## Disclaimer

All authors have an interest in increasing the acceptance of effect modeling in regulatory environmental risk assessment.

## Author Contributions Statement

**Dirk Nickisch Born Gericke**: Methodology; Writing—original draft; Writing—review & editing. **Björn Christian Rall**: Methodology; Formal analysis; Writing—original draft; Writing—review & editing. **Alexander Singer**: Methodology; Formal analysis; Writing—review & editing. **Roman Ashauer**: Conceptualization; Supervision; Writing—review & editing.

## Supporting information

This article includes online‐only Supporting Information.

Supporting information.

Supporting information.

Supporting information.

Supporting information.

## Data Availability

All needed input data for GUTS modeling are provided as Supporting Information. Data, associated metadata, and calculation tools are available as Supporting Information.
