# Supplementary material for: Fish Species Sensitivity Ranking Depends on Pesticide Exposure Profiles
Source: Environ Toxicol Chem. 2022 Jun 6;41(7):1732–41. doi: 10.1002/etc.5348 (PMC9328144; doi:10.1002/etc.5348)
Supplement: Supplementary file 1 — Supporting information. [file ETC-41-1732-s003.pdf]

SUPPLEMENTARY INFORMATION – GUTS-RED

GUTS-RED links external toxicant concentrations directly to internal damage in a one compartmental first order process. The link is described as

$$\frac{dD_w(t)}{dt} = k_D \cdot (C_w(t) - D_w(t)) \quad (1),$$

where $C_w(t)$ is the external concentration of the toxicant in water [µg a.s./L]; $D_w(t)$ is the scaled damage [µg a.s./L]; k_D is the dominant rate constant [1/d], and t is the time [d]. Equation (1), describes that the scaled damage $D_w(t)$ aims at equilibrating the external water concentration $C_w(t)$ at the speed given by the dominant rate constant, k_D.

In the ***stochastic death (SD)*** model (equation (2) below), an organism has an increased hazard rate ($h(t)$ [-]) , when the scaled damage ($D_w(t)$ [µg a.s./L]) exceeds the threshold for effects (z_w [µg a.s./L]). Above z_w, the hazard rate $h(t)$ linearly increases with $D_w(t)$ at the killing rate b_w [L/(µg a.s. d)]:

$$h(t) = b_w \cdot max(0, D_w(t) - z_w) + h_b \quad (2),$$

where h_b [1/d] is the background hazard rate (a.k.a. background or natural mortality). The survival probability of the SD-model ($S_{SD}(t)$ [-]) then calculates as

$$S_{SD}(t) = \exp(-\int_0^t h(\tau)d\tau) \quad (3).$$

The ***individual tolerance (IT)*** model assumes that each individual can tolerate damage up to an individual threshold. If the scaled damage $D_w(t)$ exceeds the threshold, the individual dies. Characteristic for the IT approach is that the tolerance thresholds differ among individuals. Therefore, the IT formulation defines a probability distribution (here the log-logistic distribution, see also EFSA PPR et al. 2018, Appendix C) for all individuals' thresholds. The survival probability of the IT-model, $S_{IT}(t)$ [-], at time t [d] is described as:

$$S_{IT}(t) = \left(1 - \frac{1}{1 + \left(\frac{1}{m_w} \cdot \max_{0 \le \tau \le t}\left(D_W(\tau)\right)\right)^{-\beta}}\right) \cdot e^{-h_b t} \quad (4).$$

where m_w [µg a.s./L] is the median of the distribution of thresholds; β [-] is the shape parameter for the distribution of thresholds; $D_W(\tau)$ [µg a.s./L] is the scaled damage; h_b [1/d] is the background hazard rate; and t [d] is the time.

TABLES SUPPLEMENTARY DATA

TABLE S1: Estimated parameters (best fit and [95%-confidence interval]) as well as model calibration quality of the GUTS-RED-SD (implementation: openGUTS standalone software) for 5 species

Species	k_D [1/d]	z_w [µg/L]	b_w [L/(µg d)]	h_b [1/d]	NRMSE [%] (< 50)	NSE [-] (> 0.5)	SPPE [%] (-50 to +50)
C. carpio	1.00 [0.335; 2.16]	4.05 [1.67; 4.84]	1.41 [0.354; 4.14]	0.00684 [0.000494; 0.0301]	3.3	1.0	-4.5 to 2.7
P. promelas	2.03 [1.02; 5.05]	3.44 [3.25; 3.54]	0.214 [0.0974; 0.541]	0.00923 [0.00779; 0.0109]	9.0	0.85	-3.0 to 8.0
C. variegatus	0.511 [0.435; 0.805]	27.6 [22.2; 28.6]	84.2 [0.210; 2230][a]	1.00e-06 [1.00e-06; 0.0144][a]	<0.001	1.0	0 to 0.00198
O. mykiss	144 [6.02; 144][a]	9.53 [7.01; 9.80]	1.00 [0.139; 18600]	1.00e-06 [1.0e-06; 0.0137][a]	6.0	0.98	-4.0e-4 to 8.9
L. macrochirus	3.17 [1.45; 144][a]	21.8 [18.7; 22.8]	0.112 [0.0382; 0.290]	1.00e-06 [1.00e-06; 0.0162][a]	2.7	1.0	-1.0e-2 to 2.0

Parameters	Quality criteria
k_D = dominant rate constant	NSE = Nash-Sutcliff model efficiency coefficient
z_w = threshold for effects	NRMSE = Normalised root-means-square error
b_w = killing rate constant	SPPE = Survival-probability prediction error
h_b = background hazard rate	

[a] Upper 95% parameter CI has run into a boundary.

TABLE S2: Estimated parameters (best fit and [95%-confidence interval]) as well as model calibration quality of the GUTS-RED-IT (implementation: openGUTS standalone software) for 5 species

**Species**	k_D **[1/d]**	m_w **[µg/L]**	***Fs*** **[-]**	h_b **[1/d]**	**NRMSE [%] (< 50)**	**NSE [-] (> 0.5)**	**SPPE [%] (-50 to +50)**
C. carpio	0.450 [0.0884; 0.730]	3.55 [0.949; 4.74]	1.38 [1.17; 2.07]	0.00689 [0.000438; 0.0304]	3.6	1.0	-6.2 to 2.8
P. promelas	0.277 [0.209; 0.450]	3.46 [3.14; 3.78]	2.11 [1.29; 3.15]	0.00859 [0.00708; 0.0104]	8.2	0.87	-9.3 to 4.8
C. variegatus	0.479 [0.333; 0.514]	27.0 [22.0; 28.2]	1.05 [1.05; 1.29][a]	1.00e-06 [1.00e-06; 0.0144][a]	0.015	1.0	-0.02 to 0.0004
O. mykiss	4.88 [1.38; 7.67]	9.76 [7.46; 11.4]	1.05 [1.05; 2.95][a]	1.00e-06 [1.00e-06; 0.0132][a]	0.036	1.0	-0.040 to 0.00040
L. macrochirus	0.940 [0.559; 1.64]	25.2 [19.3; 32.4]	1.56 [1.24; 2.52]	1.00e-06 [1.00e-06; 0.0161][a]	2.1	1.0	-0.70 to 0.089

Parameters

k_D = dominant rate constant

m_w = median of threshold distribution

Fs = factor spread[b]

h_b = background hazard rate

Quality criteria

NSE = Nash-Sutcliff model efficiency coefficient

NRMSE = Normalised root-means-square error

SPPE = Survival-probability prediction error

[a] Upper 95% parameter CI has run into a boundary.

[b] factor spread $Fs = 39^{1/beta}$, where beta is the shape parameter of the log-logistic distribution (Jager and Ashauer 2018)

TABLE S3: Estimated parameters (best fit and [95%-confidence interval]) as well as model calibration quality of the GUTS-RED-SD (implementation: openGUTS MATLAB®) for 5 species

**Species**	**k_D [1/d]**	**z_w [µg/L]**	**b_w [L/(µg d)]**	**h_b [1/d]**	**NRMSE [%] (< 50)**	**NSE [-] (> 0.5)**	**SPPE [%] (-50 to +50)**
C. carpio	1.00 [0.335; 2.16]	4.05 [1.67; 4.84]	1.41 [0.353; 4.14]	0.00684 [0.000497; 0.0301]	3.3	1.0	-4.5 to 2.7
P. promelas	2.00 [1.02; 5.05]	3.44 [3.25; 3.54]	0.214 [0.0974; 0.542]	0.00923 [0.00779; 0.0109]	9.0	0.85	-3.0 to 8.0
C. variegatus	0.509 [0.435; 0.805]	27.7 [22.2; 28.6]	210 [0.210; 2230][a]	1.00e-06 [1.00e-06; 0.0144][a]	0.001	1.0	0 to 0.00135
O. mykiss	144 [6.02; 144][b]	9.53 [7.01; 9.79]	1.02 [0.139; 18600]	1.00e-06 [1.00e-06; 0.0137][a]	6.0	0.98	-4.0e-4 to 8.9
L. macrochirus	3.17 [1.50; 144][a]	21.8 [18.7; 22.8]	0.112 [0.0380; 0.291]	1.00e-06 [1.00e-06; 0.0162][a]	2.7	1.0	-5.5e-3 to 2.0

Parameters	Quality criteria
k_D = dominant rate constant	NSE = Nash-Sutcliff model efficiency coefficient
z_w = threshold for effects	NRMSE = Normalised root-means-square error
b_w = killing rate constant	SPPE = Survival-probability prediction error
h_b = background hazard rate	

[a] Upper 95% parameter CI has run into a boundary.

TABLE S4: Estimated parameters (best fit and [95%-confidence interval]) as well as model calibration quality of the GUTS-RED-IT (implementation: <u>openGUTS MATLAB®</u>) for 5 species

**Species**	k_D **[1/d]**	m_w **[µg/L]**	***Fs*** **[-]**	h_b **[1/d]**	**NRMSE [%] (< 50)**	**NSE [-] (> 0.5)**	**SPPE [%] (-50 to +50)**
C. carpio	0.450 [0.0884; 0.727]	3.55 [0.949; 4.70]	1.38 [1.17; 2.07]	0.00689 [0.000454; 0.0303]	3.6	1.0	-6.2 to 2.8
P. promelas	0.277 [0.209; 0.450]	3.46 [3.14; 3.78]	2.11 [1.29; 3.15]	0.00859 [0.00708; 0.0104]	8.2	0.87	-9.3 to 4.8
C. variegatus	0.479 [0.333; 0.513]	27.0 [22.0; 28.2]	1.05 [1.05; 1.29][a]	1.00e-06 [1.00e-06; 0.0144][a]	0.016	1.0	-3.5e-2 to 4.0e-4
O. mykiss	4.88 [1.38; 7.67]	9.76 [7.46; 11.4]	1.05 [1.05; 2.95][a]	1.00e-06 [1.00e-06; 0.0132][a]	0.035	1.0	-0.030 to 0
L. macrochirus	0.940 [0.559; 1.64]	25.2 [19.3; 32.4]	1.56 [1.24; 2.52]	1.00e-06 [1.00e-06; 0.0161][a]	2.1	1.0	-0.70 to 0.089

Parameters	Quality criteria
k_D = dominant rate constant	NSE = Nash-Sutcliff model efficiency coefficient
m_w = median of threshold distribution	NRMSE = Normalised root-means-square error
Fs = factor spread[b]	SPPE = Survival-probability prediction error
h_b = background hazard rate	

[a] Upper 95% parameter CI has run into a boundary.

[b] factor spread $Fs = 39^{1/beta}$, where *beta* is the shape parameter of the log-logistic distribution (Jager and Ashauer 2018)

TABLE S5: Estimated parameters (best fit and [95%-confidence interval]) as well as model calibration quality of the GUTS-RED-SD (implementation: morse) for 5 species

**Species**	k_D **[1/d]**	z_w **[µg/L]**	b_w **[L/(µg d)]**	h_b **[1/d]**	**NRMSE [%] (< 50)**	**PPC [%] (> 50)**	**SPPE [%] (-50 to +50)**
C. carpio	0.817 [0.275; 2.30]	2.95 [1.39; 4.47]	0.802 [0.221; 2.77]	0.00649 [0.000724; 0.0267]	5.3	100	-7.1 to 0
P. promelas	1.97 [1.10; 6.79]	3.41 [3.10; 3.52]	0.178 [0.0667; 0.396]	0.00921 [0.00779; 0.0108]	8.9	77	-2.9 to 21
C. variegatus	0.579 [0.399; 0.923]	25.7 [19.7; 28.5]	0.455 [0.106; 3.01]	0.00228 [0.000120; 0.0168]	0	100	0 to 0
O. mykiss	15.2 [3.79; 224]	8.10 [4.48; 9.62]	0.236 [0.0698; 1.61]	0.00213 [0.000113; 0.0155]	10	100	0 to 43
L. macrochirus	3.76 [1.40; 52.7]	21.1 [15.5; 32.6]	0.0827 [0.0293; 0.394]	0.00278 [0.000138; 0.0235]	2.9	100	0 to 14

Parameters	Quality criteria
k_D = dominant rate constant	NSE = Nash-Sutcliff model efficiency coefficient
z_w = threshold for effects	NRMSE = Normalised root-means-square error
b_w = killing rate constant	SPPE = Survival-probability prediction error
h_b = background hazard rate	

TABLE S6: Estimated parameters (best fit and [95%-confidence interval]) as well as model calibration quality of the GUTS-RED-IT (implementation: morse) for 5 species

**Species**	***k_D*** **[1/d]**	***m_w*** **[µg/L]**	***beta*** **[-]**	***h_b*** **[1/d]**	**NRMSE [%] (< 50)**	**PPC [%] (> 50)**	**SPPE [%] (-50 to +50)**
C. carpio	0.307 [0.0825; 0.628]	2.70 [0.902; 4.38]	8.32 [4.13; 17.8]	0.00660 [0.000773; 0.0272]	4.6	100	-7.1 to 0
P. promelas	0.281 [0.207; 0.408]	3.49 [3.15; 3.78]	4.97 [3.14; 11.0]	0.00860 [0.00705; 0.0103]	8.1	79	-14 to 4.8
C. variegatus	0.407 [0.156; 0.509]	24.9 [13.6; 28.2]	22.2 [7.21; 76.8]	0.00225 [0.000112; 0.0181]	4.1	100	-0.030 to 0.00040
O. mykiss	2.69 [1.19; 7.67]	9.31 [6.94; 11.2]	7.32 [2.76; 46.4]	0.00217 [0.000106; 0.0158]	0	100	0 to 0
L. macrochirus	0.923 [0.505; 1.70]	24.9 [18.1; 33.2]	7.19 [3.38; 17.5]	0.00247 [0.000118; 0.0203]	0	100	0 to 0

Parameters

k_D = dominant rate constant

m_w = median of threshold distribution

beta = shape of threshold distribution

h_b = background hazard rate

Quality criteria

NSE = Nash-Sutcliff model efficiency coefficient

NRMSE = Normalised root-means-square error

SPPE = Survival-probability prediction error

TABLE S7: HP_5 comparison for GUTS-RED-SD and GUTS-RED-IT implementations

**Scenario**	***openGUTS standalone***		***openGUTS MATLAB®***		***morse***	
	**GUTS-RED-SD**	**GUTS-RED-IT**	**GUTS-RED-SD**	**GUTS-RED-IT**	**GUTS-RED-SD**	**GUTS-RED-IT**
A	10.2	10.2	10.2	10.2	9.3	9.2
B	18.5	24.0	18.5	24.0	21.3	24.9
C	21.1	31.4	21.1	31.2	23.3	33.4

FIGURES SUPPLEMENTARY DATA

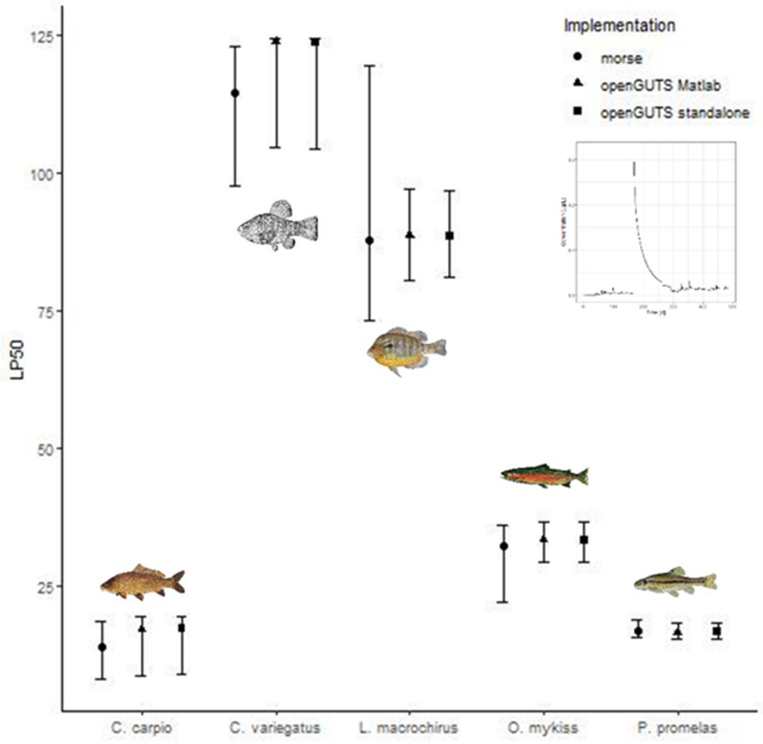

Figure S1: LP_{50} comparison for GUTS-RED-SD implementations (circle = morse, triangle = openGUTS MATLAB®, square = openGUTS standalone) across five different fish species: a) sheepshead minnow; b) common carp; c) bluegill; d) rainbow trout; and e) fathead minnow (acute and ELS test) for exposure scenario A. For openGUTS versions, predictions were conducted from the best fit (dot) and the parameter sample (95% confidence interval - vertical line), for morse from the posterior distribution (median - dot and 95% credible intervals - vertical line).

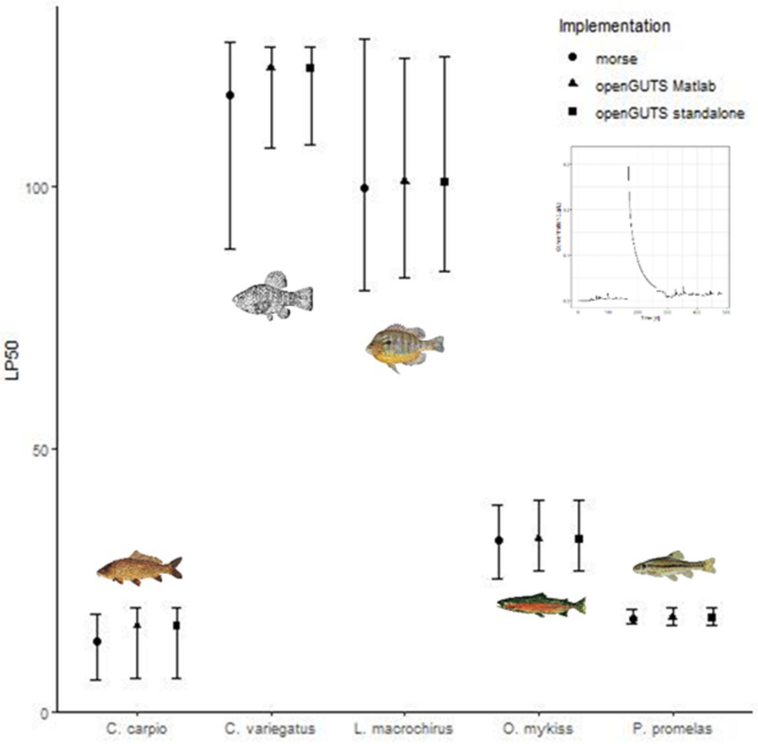

Figure S2: LP_{50} comparison for GUTS-RED-IT implementations (circle = morse, triangle = openGUTS MATLAB®, square = openGUTS standalone) across five different fish species: a) sheepshead minnow; b) common carp; c) bluegill; d) rainbow trout; and e) fathead minnow (acute and ELS test) for exposure scenario A. For openGUTS versions, predictions were conducted from the best fit (dot) and the parameter sample (95% confidence interval - vertical line), for morse from the posterior distribution (median - dot and 95% credible intervals - vertical line).

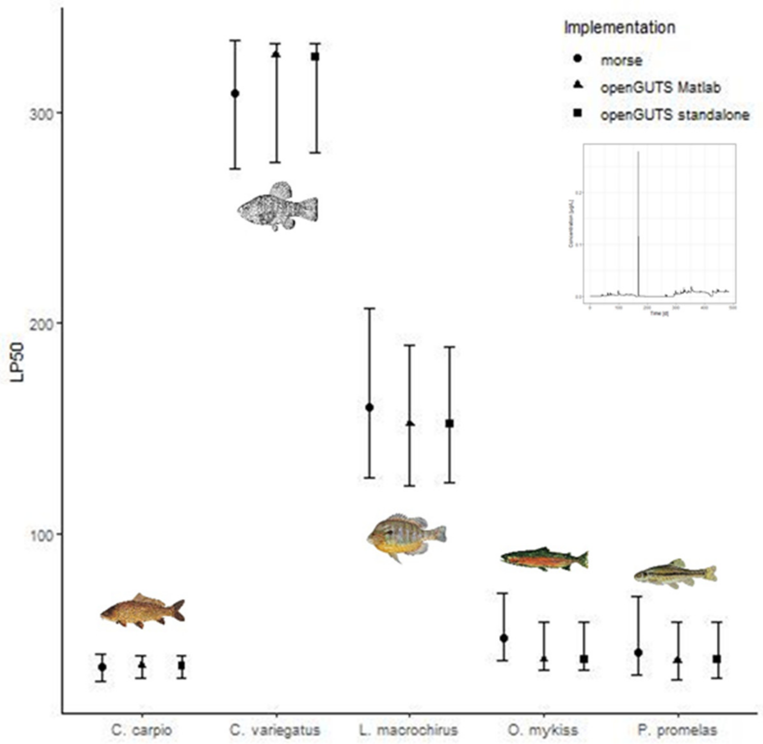

Figure S3 LP_{50} comparison for GUTS-RED-SD implementations (circle = morse, triangle = openGUTS MATLAB®, square = openGUTS standalone) across five different fish species: a) sheepshead minnow; b) common carp; c) bluegill; d) rainbow trout; and e) fathead minnow (acute and ELS test) for exposure scenario B. For openGUTS versions, predictions were conducted from the best fit (dot) and the parameter sample (95% confidence interval - vertical line), for morse from the posterior distribution (median - dot and 95% credible intervals - vertical line).

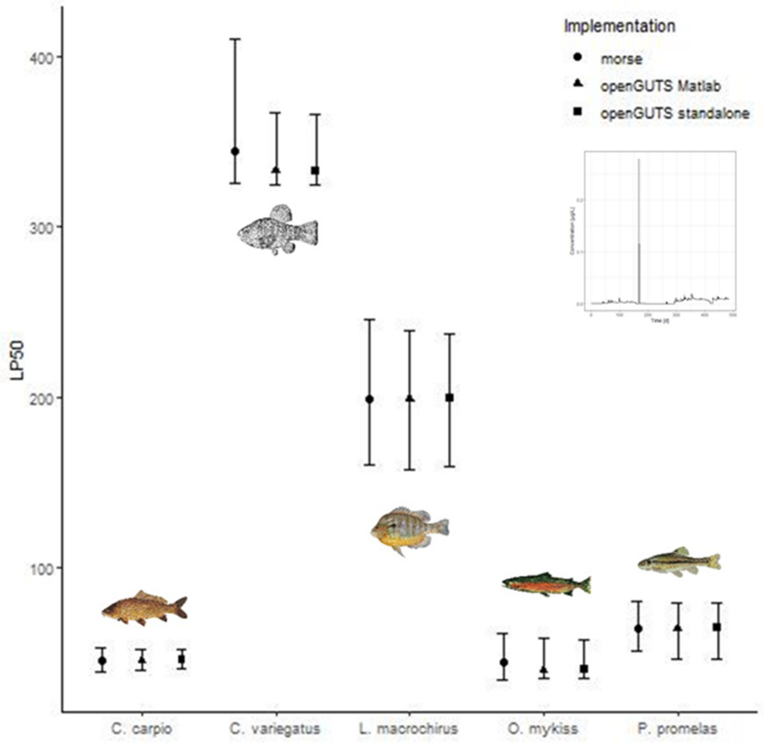

Figure S4: LP_{50} comparison for GUTS-RED-IT implementations (circle = morse, triangle = openGUTS MATLAB®, square = openGUTS standalone) across five different fish species: a) sheepshead minnow; b) common carp; c) bluegill; d) rainbow trout; and e) fathead minnow (acute and ELS test) for exposure scenario B. For openGUTS versions, predictions were conducted from the best fit (dot) and the parameter sample (95% confidence interval - vertical line), for morse from the posterior distribution (median - dot and 95% credible intervals - vertical line).

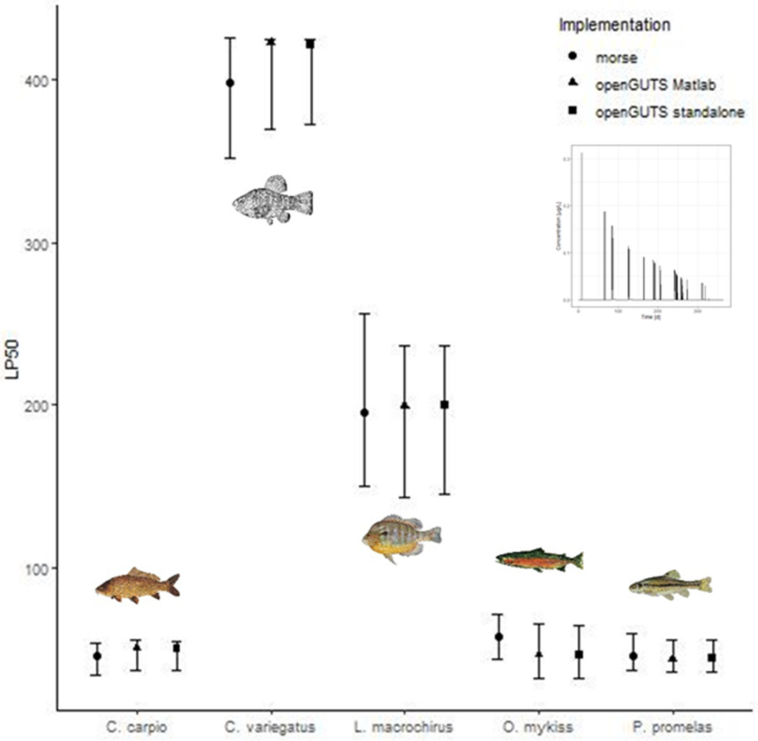

Figure S5: LP_{50} comparison for GUTS-RED-SD implementations (circle = morse, triangle = openGUTS MATLAB®, square = openGUTS standalone) across five different fish species: a) sheepshead minnow; b) common carp; c) bluegill; d) rainbow trout; and e) fathead minnow (acute and ELS test) for exposure scenario C. For openGUTS versions, predictions were conducted from the best fit (dot) and the parameter sample (95% confidence interval - vertical line), for morse from the posterior distribution (median - dot and 95% credible intervals - vertical line).

SSD openGUTS MATLAB® and morse

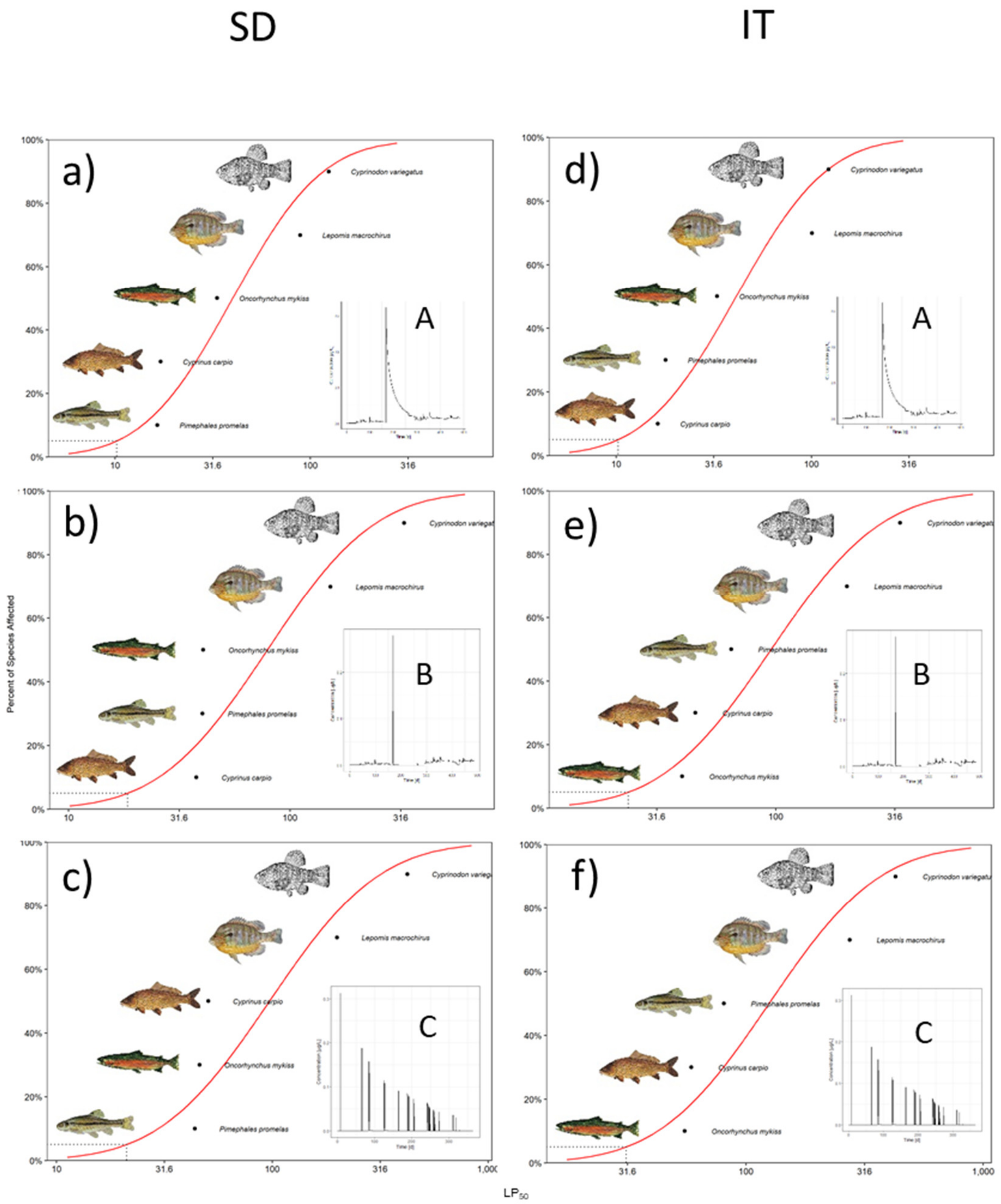

Figure S6: SSDs (red line) are cumulative probability distributions that estimate the percent of species that are affected by a given concentration of a chemical. The profile that affects 5% of the species is referred to as the 5% hazard profile (HP5 - black dashed line). Here, SSD was built on LP_{50} estimated with GUTS-RED (**openGUTS MATLAB®** is shown). Graphs a – c show results for GUTS-RED-SD, graphs d – f show results for GUTS-RED-IT. A, B, C describes exposure profiles (shown in FIGURE 1)

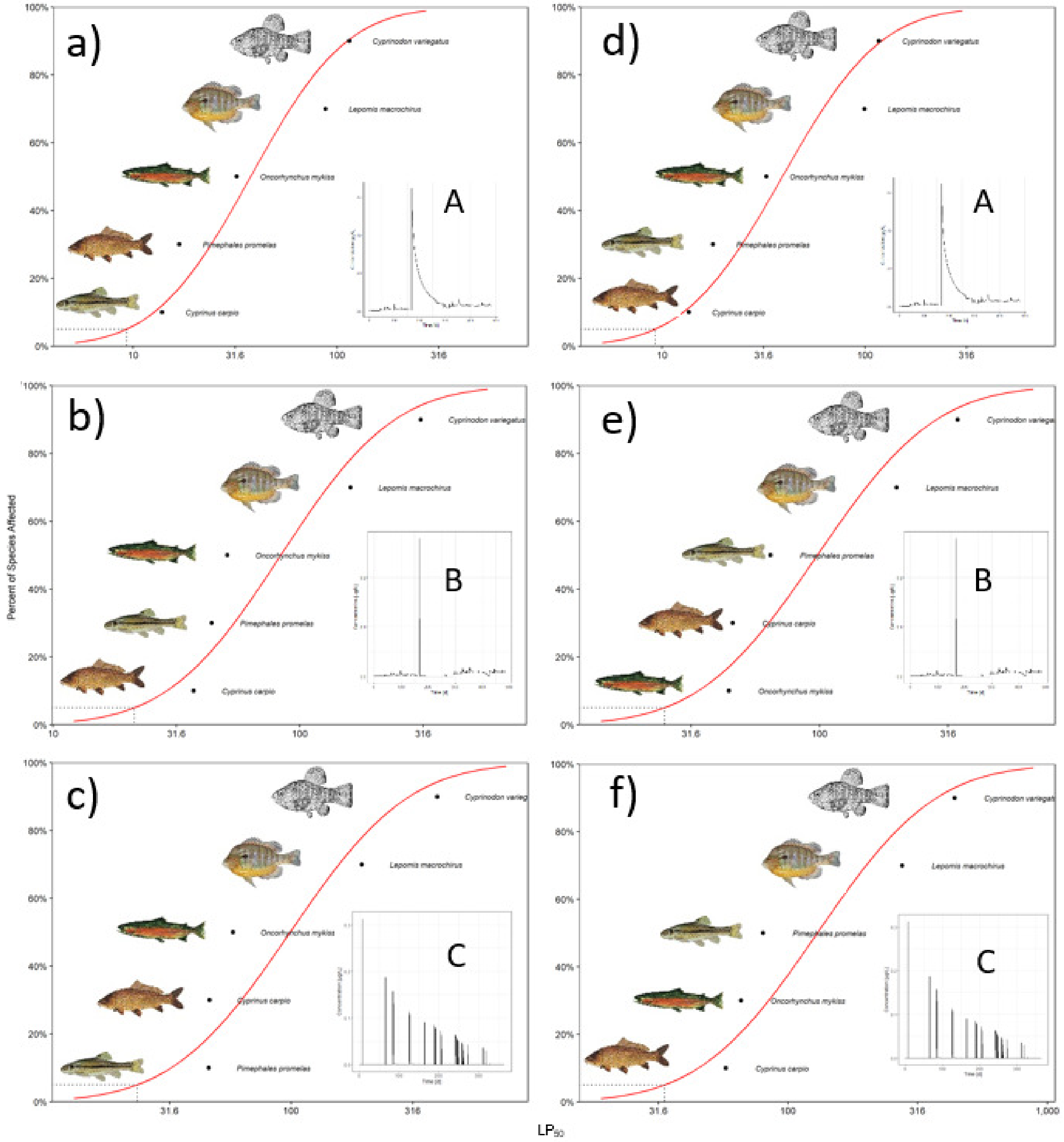

Figure S7: SSDs (red line) are cumulative probability distributions that estimate the percent of species that are affected by a given concentration of a chemical. The profile that affects 5% of the species is referred to as the 5% hazard profile (HP5 - black dashed line). Here, SSD was built on LP_{50} estimated with GUTS-RED (**morse** is shown). Graphs a – c show results for GUTS-RED-SD, graphs d – f show results for GUTS-RED-IT. A, B, C describes exposure profiles (shown in FIGURE 1)

Data for calibration for fathead minnow using the acute test only

Data are presented below where only the acute test was used to calibrate fathead minnow, without the ELS test. The standalone openGUTS implementation was used.

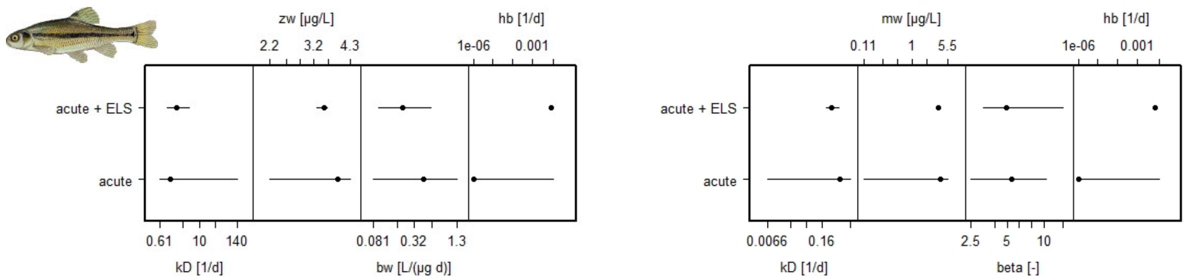

FIGURE S8: Model comparison for GUTS-RED-SD (left panel) and GUTS-RED-IT (right panel) for the openGUTS standalone implementation and species fathead minnow. Calibration was performed using the acute and ELS test and using only the acute test. Estimated parameters for GUTS-RED-SD are dominant rate constant (kD), threshold for effects (zw) and killing rate constant (bw). Estimated parameters for GUTS-RED-IT are dominant rate constant (kD), median of the threshold distribution (mw) and the shape parameter for the distribution of thresholds (beta). The best fit (dot) and 95% confidence interval (horizontal line) are displayed. No mortality was observed in the control runs of the 96-hour acute test, so hb runs against the lower limit. Mortality was observed in the 28-day ELS test, especially on day 6. As this mortality occurred to the same extent in control and all lower exposure treatments, it was fully captured by background mortality (hb, see openGUTS standalone reports in supplementary data). Therefore, the largest differences between the two calibrations were observed for background mortality. Uncertainties of the other parameters were significantly higher in the calibration with only the acute test than in the calibration with the acute and ELS tests. The best fit parameters of the calibrationwith combined data (acute + ELS) were within the uncertainties of the calibration with acute test data.

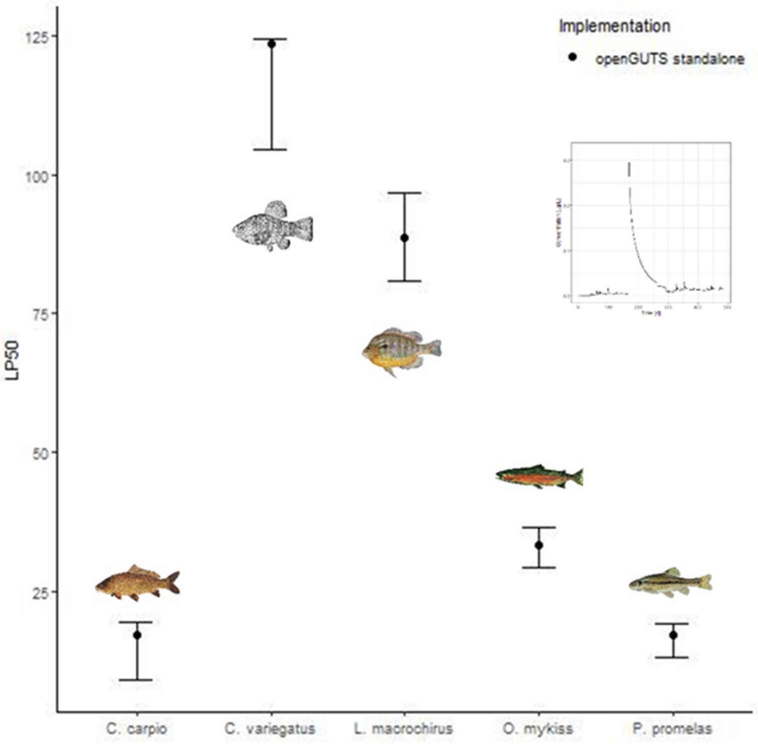

Figure S9: LP_{50} comparison for GUTS-RED-SD implementations (openGUTS standalone) across five different fish species: a) sheepshead minnow; b) common carp; c) bluegill; d) rainbow trout; and e) fathead minnow (**acute test only**) for exposure **scenario A**. Predictions were conducted from the best fit (dot) and the parameter sample (95% confidence interval - vertical line).

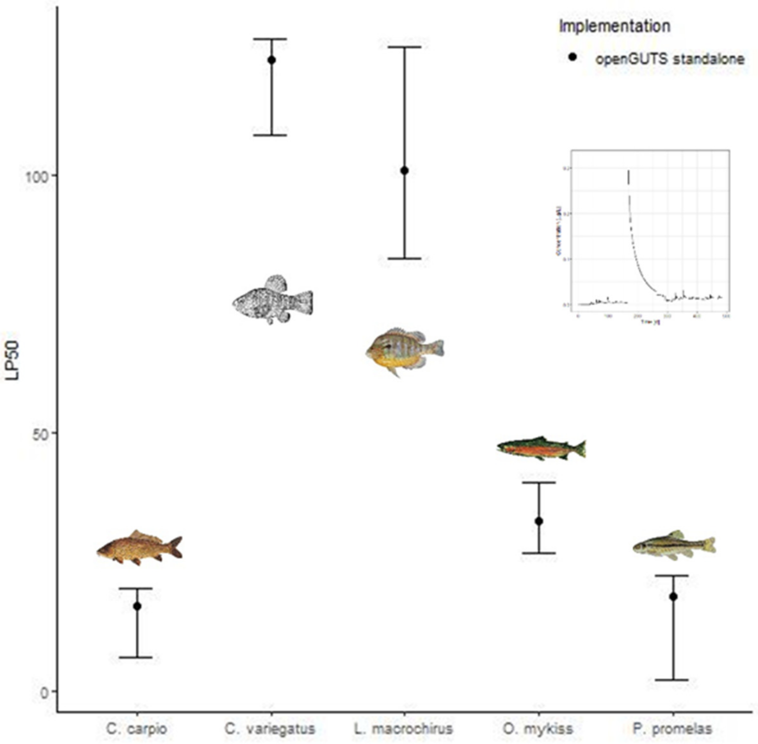

Figure S10: LP_{50} comparison for GUTS-RED-IT (openGUTS standalone) across five different fish species: a) sheepshead minnow; b) common carp; c) bluegill; d) rainbow trout; and e) fathead minnow (**acute test only**) for exposure **scenario A**. Predictions were conducted from the best fit (dot) and the parameter sample (95% confidence interval - vertical line).

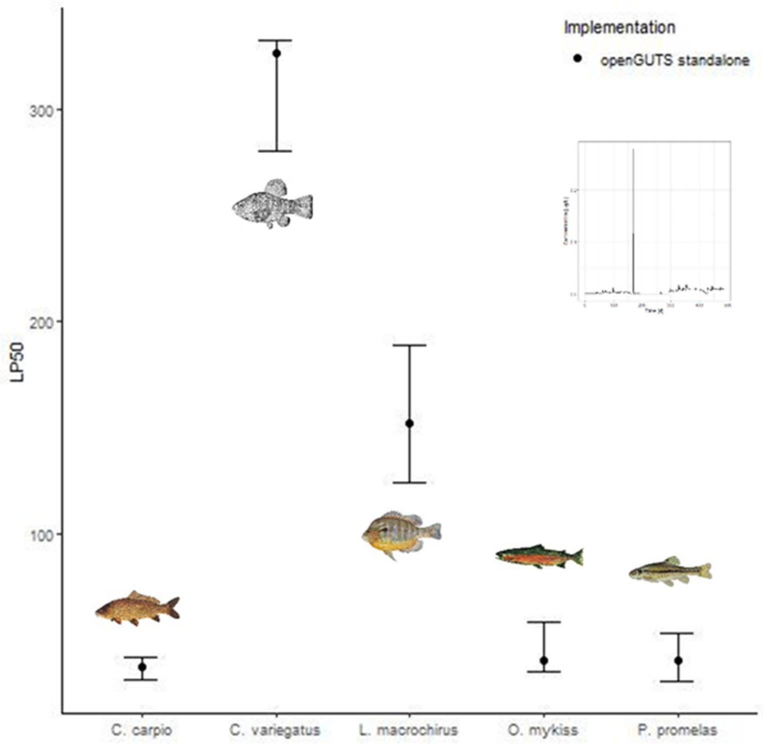

Figure S11 LP_{50} comparison for GUTS-RED-SD (openGUTS standalone) across five different fish species: a) sheepshead minnow; b) common carp; c) bluegill; d) rainbow trout; and e) fathead minnow (**acute test only**) for exposure **scenario B**. Predictions were conducted from the best fit (dot) and the parameter sample (95% confidence interval - vertical line).

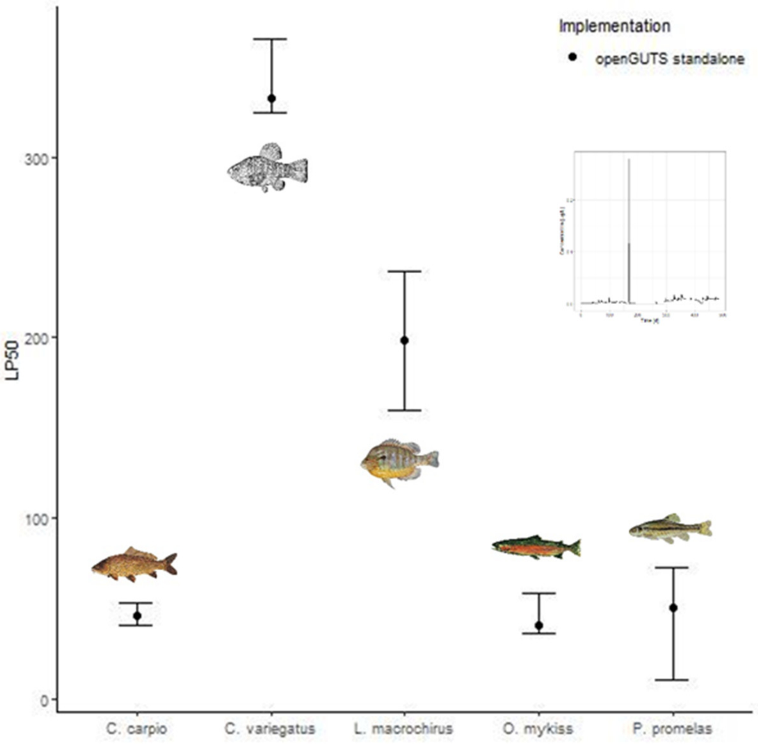

Figure S12: LP_{50} comparison for GUTS-RED-IT (openGUTS standalone) across five different fish species: a) sheepshead minnow; b) common carp; c) bluegill; d) rainbow trout; and e) fathead minnow (**acute test only**) for exposure **scenario B**. Predictions were conducted from the best fit (dot) and the parameter sample (95% confidence interval - vertical line).

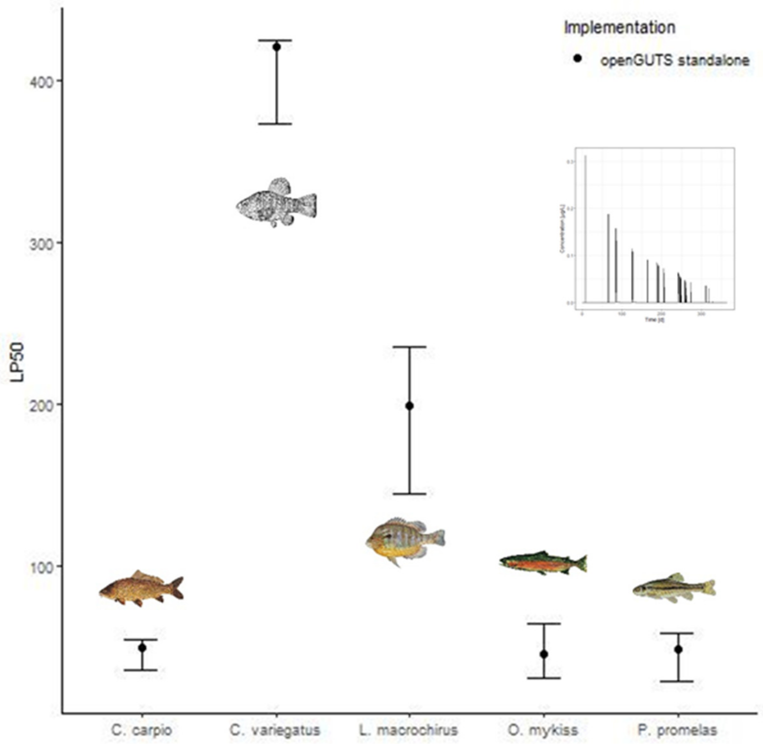

Figure S13: LP_{50} comparison for GUTS-RED-SD (openGUTS standalone) across five different fish species: a) sheepshead minnow; b) common carp; c) bluegill; d) rainbow trout; and e) fathead minnow (**acute test only**) for exposure **scenario C**. Predictions were conducted from the best fit (dot) and the parameter sample (95% confidence interval - vertical line).

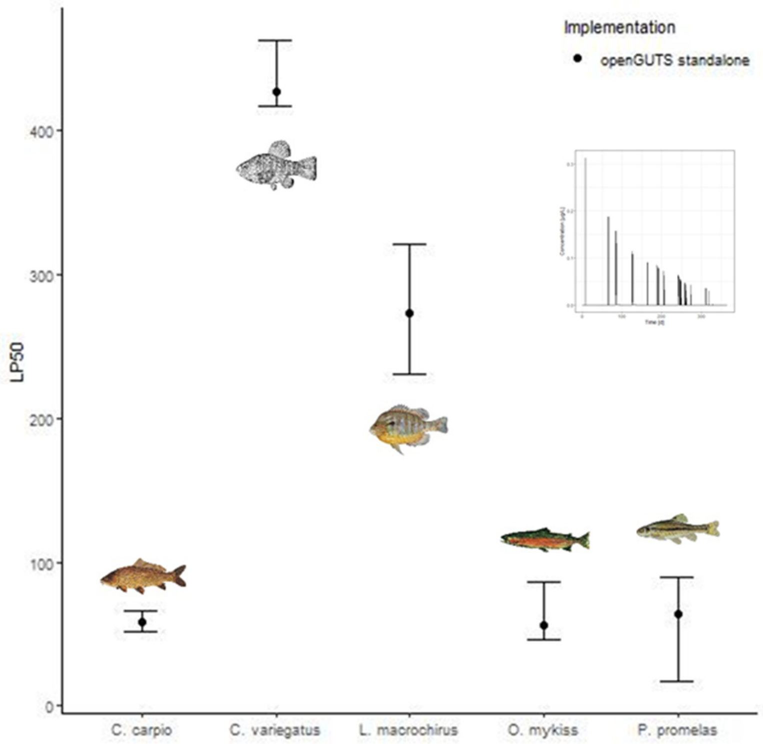

Figure S14: LP_{50} comparison for GUTS-RED-IT (openGUTS standalone) across five different fish species: a) sheepshead minnow; b) common carp; c) bluegill; d) rainbow trout; and e) fathead minnow (**acute test only**) for exposure **scenario C**. Predictions were conducted from the best fit (dot) and the parameter sample (95% confidence interval - vertical line).

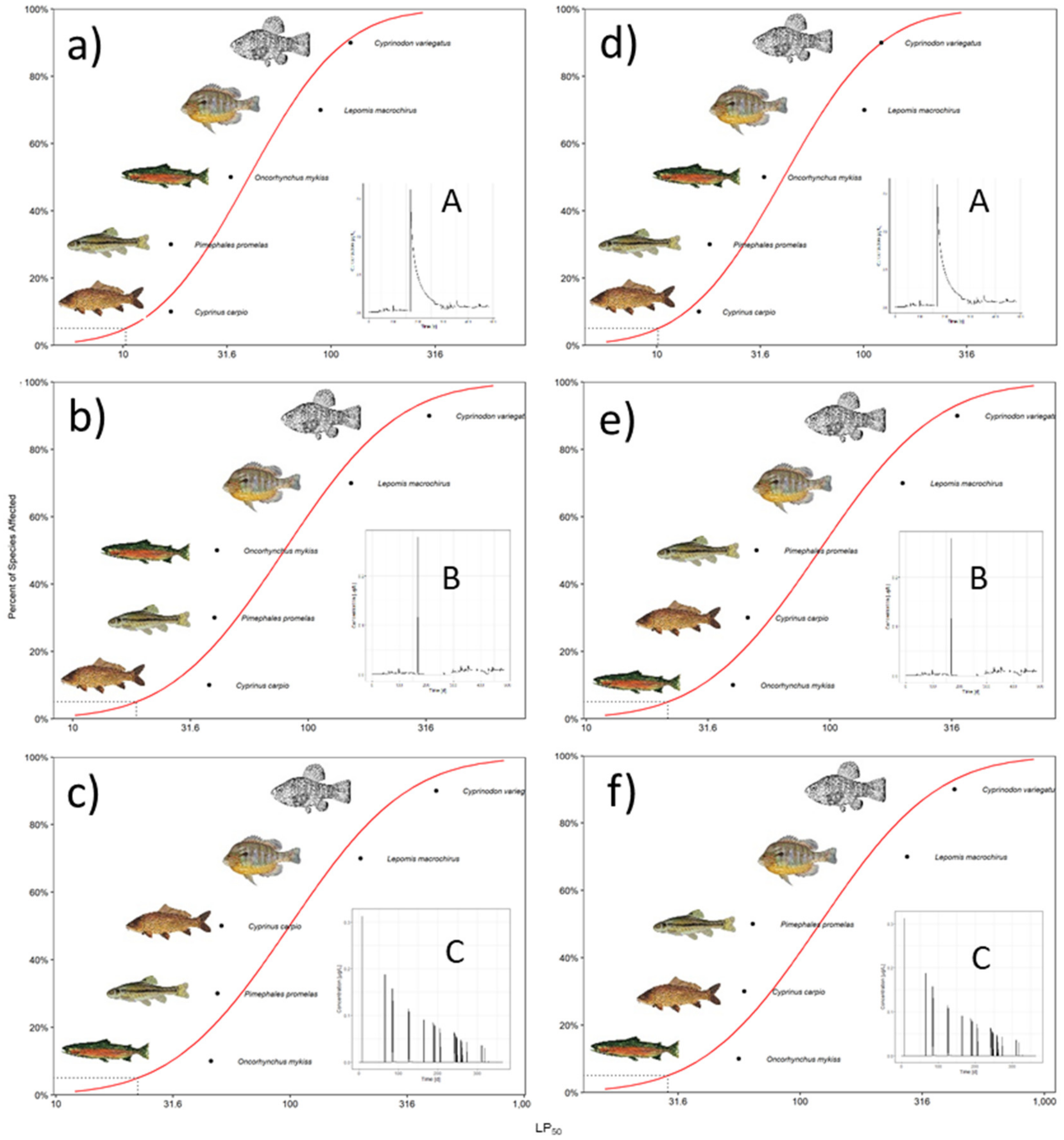

Figure S15: SSDs (red line) are cumulative probability distributions that estimate the percent of species that are affected by a given concentration of a chemical. The concentration that affects 5% of the species is referred to as the 5% Hazard Concentration (black dashed line). Here, the SSDs were built on LP_{50} estimated with GUTS-RED (openGUTS standalone implementation is shown). Calibration for *P. promelas* was performed **using the acute test only**. Graphs a – c show results for GUTS-RED-SD, graphs d – f show results for GUTS-RED-IT. A, B, C describes exposure profiles (shown in FIGURE 1). Best-fit LP_{50} values for *P. promelas* were lower for GUTS-RED-IT and scenarios B and C (panels e and f) than when calibrated with acute and ELS tests (FIGURE 5). However, this did not affect the order of sensitivities. Although lower deviations from the best-fit LP_{50} were observed for GUTS-RED-SD, the order of species changed for Scenario A and C. Note, the differences in LP_{50} estimates of models calibrated with acute and ELS or only acute test data ranged within the 95% uncertainty bounds. Therefore, the impact of the additional ELS data on LP_{50} predictions cannot be distinguished from the projection uncertainty.

FISH PICTOGRAMS

All pictograms are in the public domain:

US government. Drawing of male freshwater phase Steelhead (Oncorhynchus mykiss) [accessed 2021 Apr 28]. https://commons.wikimedia.org/wiki/File:Lake_Washington_Ship_Canal_Fish_Ladder_pamphlet_-_male_freshwater_phase_Steelhead.jpg.

United States Great Lakes Environmental Research Laboratory (GLERL). Cyprinus carpio GLERL 1.jpg. [accessed 2021 Apr 28]. https://commons.wikimedia.org/wiki/File:Cyprinus_carpio_GLERL_1.jpg.

U.S. Fish and Wildlife Service DRSF and W. Illustration of Pimephales promelas, the fathead minnow. [accessed 2021 Apr 28]. https://commons.wikimedia.org/wiki/File:Pimephales_promelas2.jpg.

U.S. National Oceanic and Atmospheric Administration. Sheepshead minnow.jpg. [accessed 2021a Apr 28]. https://commons.wikimedia.org/wiki/File:Sheepshead_minnow.jpg.

U.S. National Oceanic and Atmospheric Administration. Lepomis macrochirus.jpg. [accessed 2021b Apr 28]. https://de.wikipedia.org/wiki/Datei:Lepomis_macrochirus.jpg.
