# Supplementary material for: Fish Species Sensitivity Ranking Depends on Pesticide Exposure Profiles
Source: Environ Toxicol Chem. 2022 Jun 6;41(7):1732–41. doi: 10.1002/etc.5348 (PMC9328144; doi:10.1002/etc.5348)
Supplement: Supplementary file 2 — Supporting information. [file ETC-41-1732-s004.zip › fits openGUTS standalone/calibration_jointFitHb_C_carpio.pdf]

openGUTS Report

Project:

calibration_jointFitHb

Project file:

Project description (optional):

No project description available

Software version:

openGUTS - 1.0

Date of report creation:

30/05/2020 07:41:34

Calibration

Calibration input data

Data set 1

File:

Description (optional):

Control group: 'acute 0 µg a.s./L'

Survival data of input data set 1:

Time [d]	acute 0 µg a.s./L	acute 0.630 µg a.s./L	acute 1.30 µg a.s./L	acute 2.50 µg a.s./L	acute 5.00 µg a.s./L	acute 10.0 µg a.s./L
0	14	7	7	7	7	7
1	14	7	7	7	7	3
2	14	7	7	7	5	0
3	14	7	7	7	2	0
4	13	7	7	7	0	0

Concentration data of input data set 1:

Time [d]	acute 0 µg a.s./L	acute 0.630 µg a.s./L	acute 1.30 µg a.s./L	acute 2.50 µg a.s./L	acute 5.00 µg a.s./L	acute 10.0 µg a.s./L
0	0	0.61	1.1	2.3	5.4	10

Calibration settings

Calibration parameter settings for GUTS-RED-SD:

Parameter	Fit	Min	Max	Scale
kd	Yes	0.001641	143.8	Log
mw	Yes	0.0001669	9.9	Norm
hb	Yes	1E-6	0.07	Norm
bw	Yes	0.002634	33445	Log
Fs	No	1	1	Norm

Calibration parameter settings for GUTS-RED-IT:

Parameter	Fit	Min	Max	Scale
kd	Yes	0.001641	8.35	Log
mw	Yes	0.0001669	15.19	Log
hb	Yes	1E-6	0.07	Norm
bw	No	Inf	Inf	Norm
Fs	Yes	1.05	20	Log

Calibration results

Fitted parameters for GUTS-RED-SD:

Best fit parameter values and their 95% CI

kd: 1.002 (0.3348 - 2.155)

mw: 4.046 (1.666 - 4.835)

hb: 0.006835 (0.0004942 - 0.0301)

bw: 1.409 (0.3535 - 4.138)

* edge of 95% parameter CI has run into a boundary

(this may also affect CIs of other parameters)

Goodness of fit for calibration data (GUTS-RED-SD):

Model efficiency (NSE, r-square): 0.9959

Normalised root-means-square error (NRMSE): 3.317 %

Minus log-likelihood (MLL): 18.92

AIC: 45.84

Survival probability prediction error (SPPE) for each treatment:

Data set	Treatment	Value
1	acute 0 µg a.s./L	-4.45 %
1	acute 0.630 µg a.s./L	2.697 %
1	acute 1.30 µg a.s./L	2.697 %
1	acute 2.50 µg a.s./L	2.697 %
1	acute 5.00 µg a.s./L	-3.85 %
1	acute 10.0 µg a.s./L	-6.73E-8 %

GUTS-RED-SD results table for LCx,t [[C]], with 95% CI:

Time [d]	LC50	LC20	LC10
1	9.588 (7.702 - 13.77)	8.001 (6.163 - 9.734)	7.444 (5.42 - 8.927)
2	5.874 (4.934 - 6.543)	5.284 (3.77 - 5.736)	5.074 (3.348 - 5.535)
3	4.926 (3.625 - 5.435)	4.59 (2.983 - 5.097)	4.473 (2.743 - 5.008)
4	4.552 (2.991 - 5.159)	4.329 (2.577 - 4.966)	4.253 (2.422 - 4.91)
7	4.219 (2.173 - 4.95)	4.122 (1.973 - 4.868)	4.092 (1.903 - 4.845)

14	4.102 (1.756 - 4.869)	4.066 (1.688 - 4.836)	4.056 (1.664 - 4.827)
21	4.078 (1.67 - 4.85)	4.057 (1.633 - 4.829)	4.051 (1.621 - 4.824)
28	4.068 (1.641 - 4.841)	4.053 (1.617 - 4.826)	4.049 (1.61 - 4.822)
42	4.06 (1.622 - 4.833)	4.05 (1.608 - 4.823)	4.048 (1.605 - 4.821)
50	4.057 (1.617 - 4.83)	4.049 (1.606 - 4.823)	4.047 (1.604 - 4.821)
100	4.051 (1.607 - 4.824)	4.047 (1.603 - 4.821)	4.046 (1.602 - 4.82)

Plots for GUTS-RED-SD calibration:

Parameter space plot for the calibration of GUTS-RED-SD:

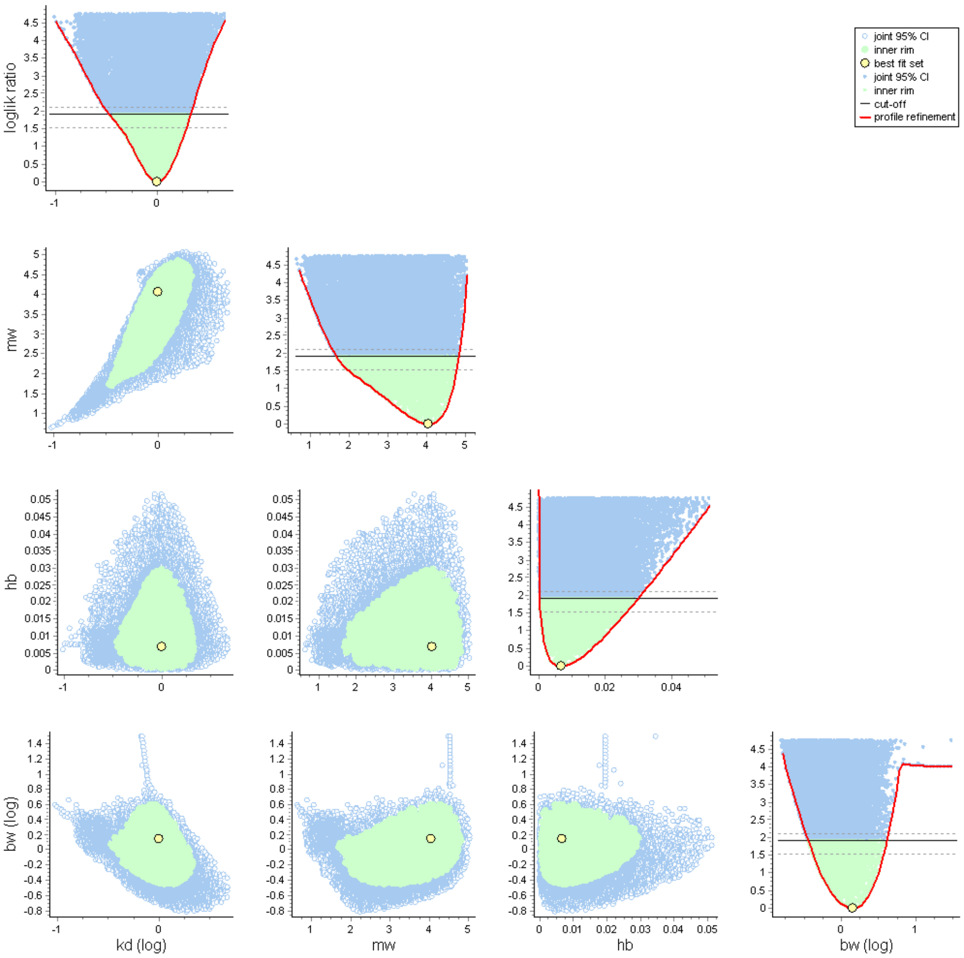

Exposure, damage and survival plots for the calibration of GUTS-RED-SD:

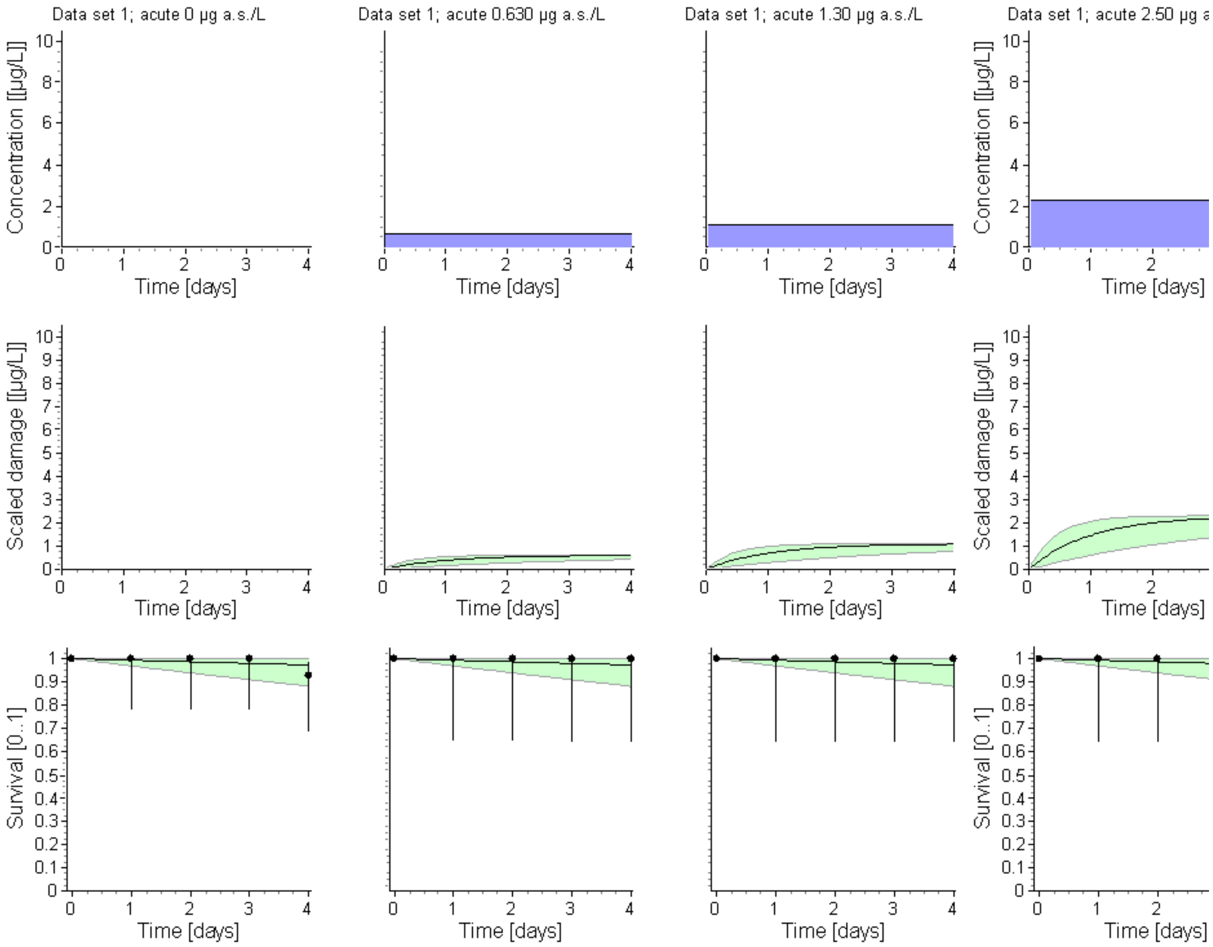

... continued plot:

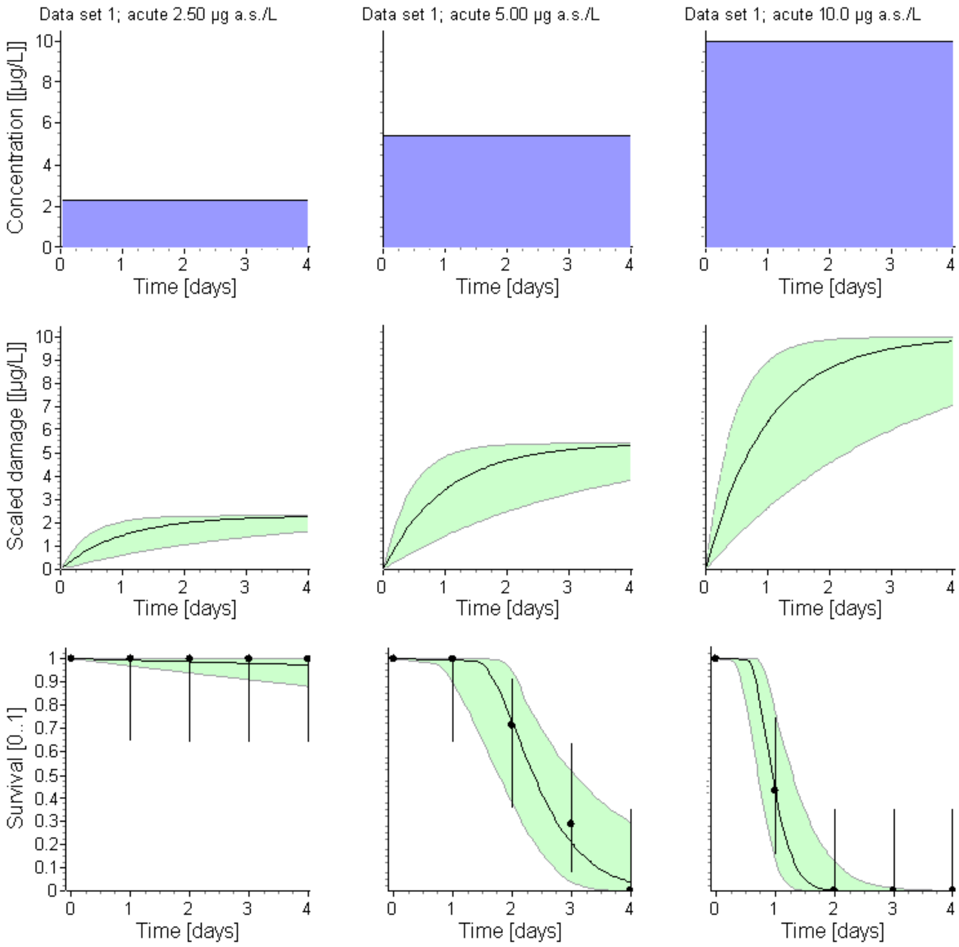

Observed vs. Predicted survival plot for the calibration of GUTS-RED-SD:

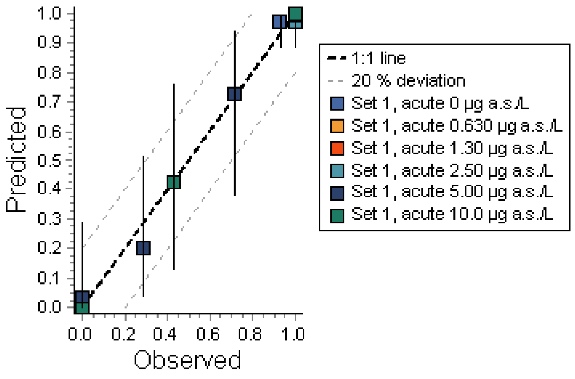

Observed vs. Predicted deaths plot for the calibration of GUTS-RED-SD:

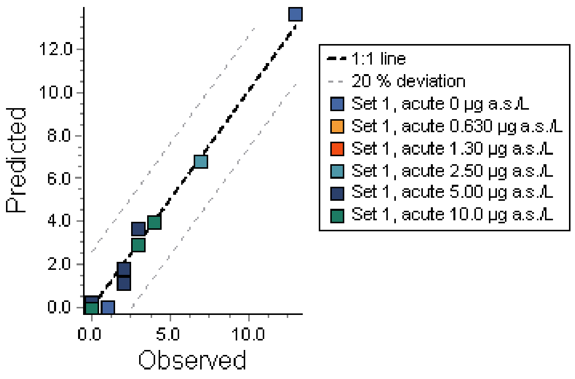

LCx versus time with confidence intervals (plotted for 16 days, GUTS-RED-SD):

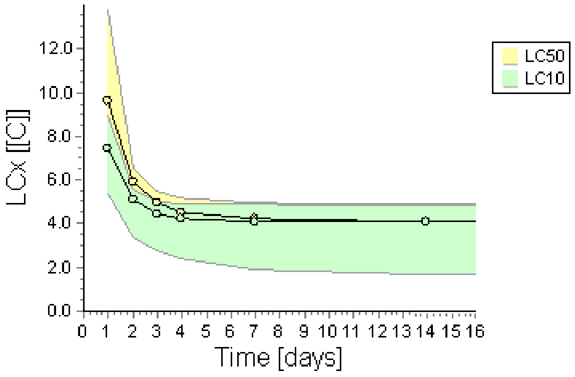

Fitted parameters for GUTS-RED-IT:

Best fit parameter values and their 95% CI

kd: 0.45 (0.08841 - 0.73)

mw: 3.551 (0.9487 - 4.735)

hb: 0.006891 (0.0004375 - 0.03035)

Fs: 1.384 (1.17 - 2.073)

* edge of 95% parameter CI has run into a boundary

(this may also affect CIs of other parameters)

Goodness of fit for calibration data (GUTS-RED-IT):

Model efficiency (NSE, r-square): 0.9947

Normalised root-means-square error (NRMSE): 3.605 %

Minus log-likelihood (MLL): 19.32

AIC: 46.63

Survival probability prediction error (SPPE) for each treatment:

Data set	Treatment	Value
1	acute 0 µg a.s./L	-4.42 %
1	acute 0.630 µg a.s./L	2.719 %
1	acute 1.30 µg a.s./L	2.719 %
1	acute 2.50 µg a.s./L	2.813 %
1	acute 5.00 µg a.s./L	-6.2 %
1	acute 10.0 µg a.s./L	-0.01 %

GUTS-RED-IT results table for LCx,t [[C]], with 95% CI:

Time [d]	LC50	LC20	LC10
1	9.8 (8.563 - 11.98)	8.666 (7.065 - 9.867)	8.064 (6.177 - 9.179)
2	5.984 (5.232 - 6.646)	5.292 (4.181 - 5.904)	4.924 (3.603 - 5.685)
3	4.794 (3.836 - 5.441)	4.239 (3.068 - 4.94)	3.945 (2.658 - 4.727)
4	4.255 (3.061 - 5.041)	3.762 (2.465 - 4.586)	3.501 (2.136 - 4.341)
7	3.71 (1.988 - 4.771)	3.281 (1.602 - 4.327)	3.053 (1.391 - 4.096)
14	3.558 (1.268 - 4.741)	3.146 (1.024 - 4.292)	2.928 (0.8969 - 4.062)

21	3.552 (1.041 - 4.741)	3.141 (0.8426 - 4.292)	2.923 (0.7435 - 4.062)
28	3.551 (0.9425 - 4.741)	3.14 (0.7627 - 4.292)	2.922 (0.6738 - 4.062)
42	3.551 (0.8659 - 4.741)	3.14 (0.7007 - 4.292)	2.922 (0.6191 - 4.062)
50	3.551 (0.8494 - 4.741)	3.14 (0.6873 - 4.292)	2.922 (0.6072 - 4.062)
100	3.551 (0.8308 - 4.741)	3.14 (0.6723 - 4.292)	2.922 (0.594 - 4.062)

Plots for GUTS-RED-IT calibration:

Parameter space plot for the calibration of GUTS-RED-IT:

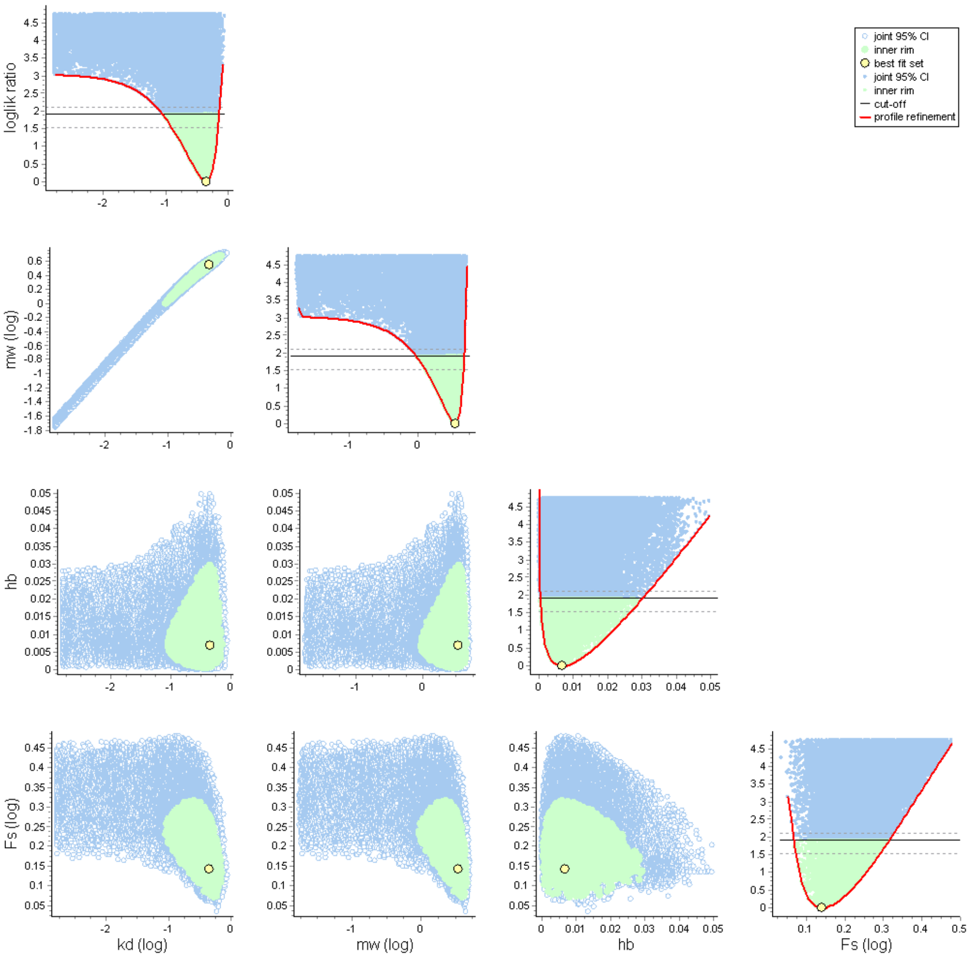

Exposure, damage and survival plots for the calibration of GUTS-RED-IT:

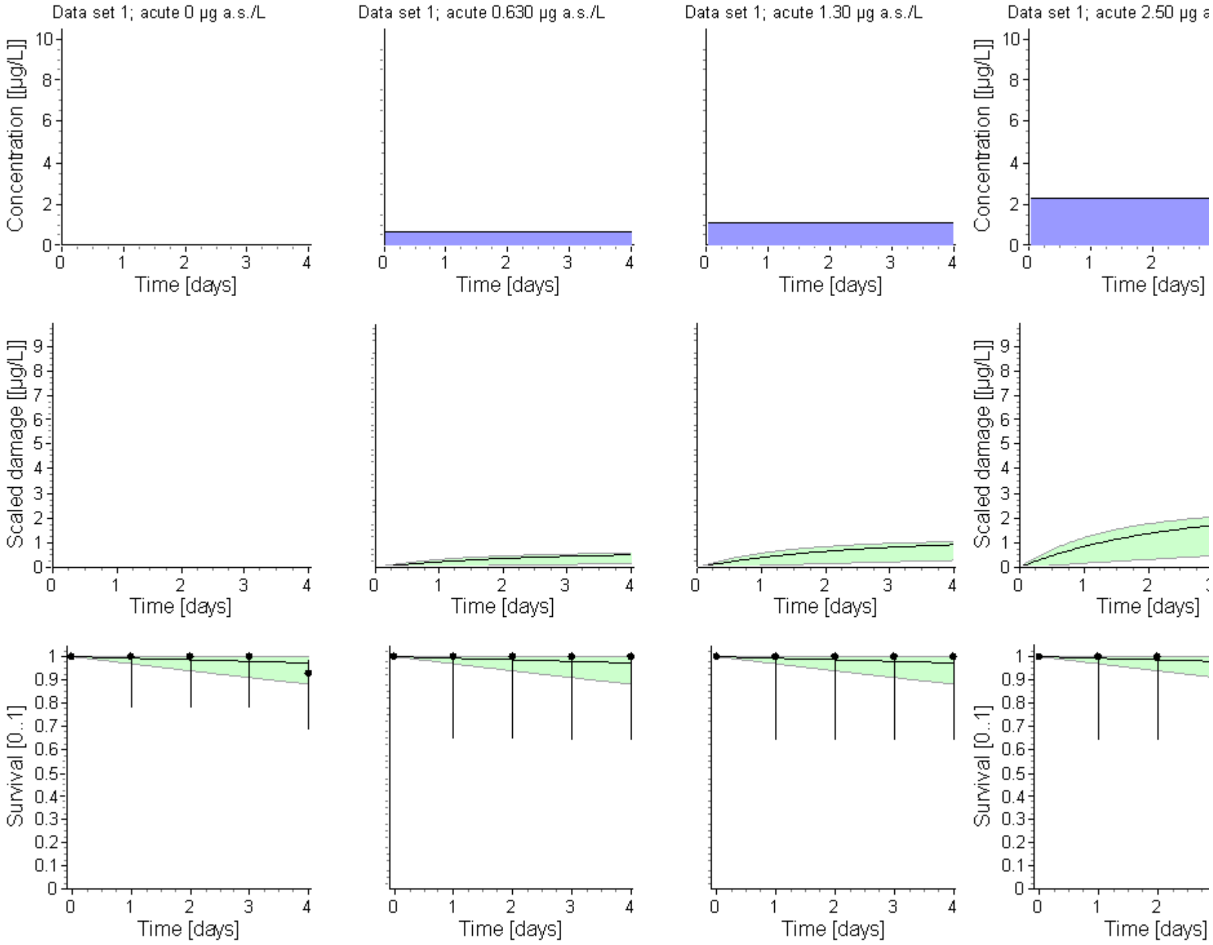

... continued plot:

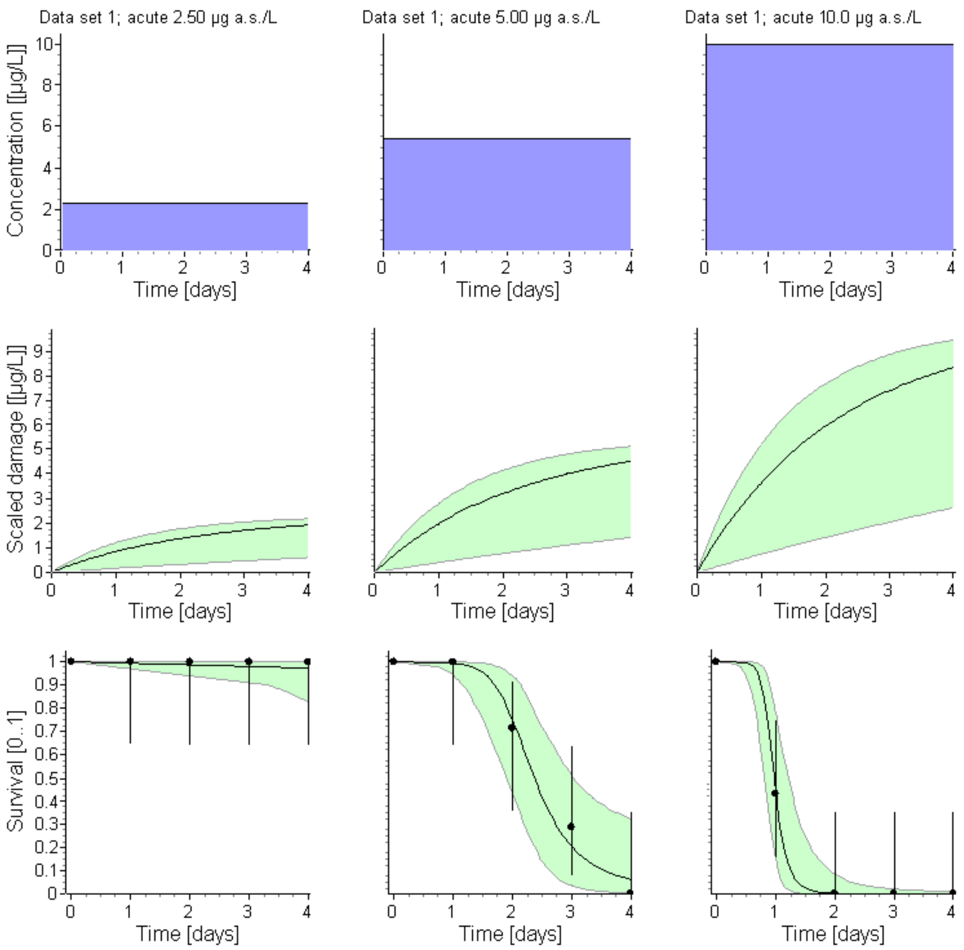

Observed vs. Predicted survival plot for the calibration of GUTS-RED-IT:

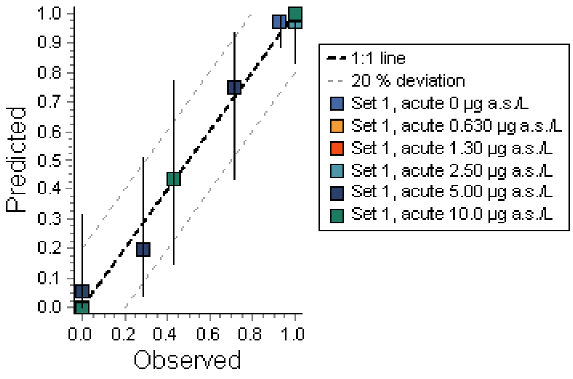

Observed vs. Predicted deaths plot for the calibration of GUTS-RED-IT:

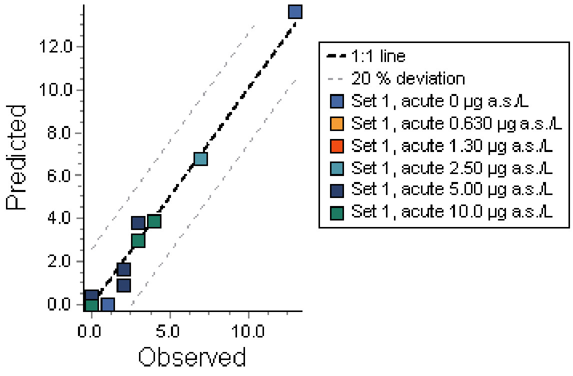

LCx versus time with confidence intervals (plotted for 16 days, GUTS-RED-IT):

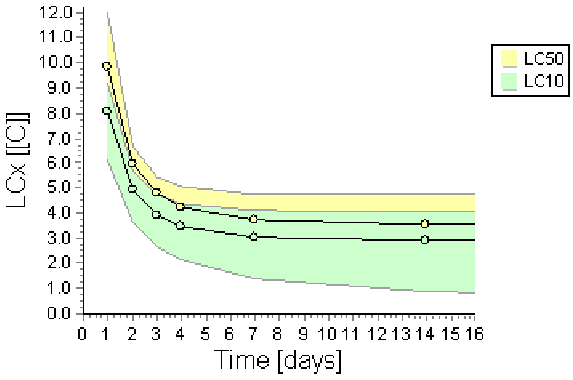

Validation

No validation performed!

Predictions

No predictions performed!
