# Supplementary material for: Fish Species Sensitivity Ranking Depends on Pesticide Exposure Profiles
Source: Environ Toxicol Chem. 2022 Jun 6;41(7):1732–41. doi: 10.1002/etc.5348 (PMC9328144; doi:10.1002/etc.5348)
Supplement: Supplementary file 2 — Supporting information. [file ETC-41-1732-s004.zip › fits openGUTS standalone/calibration_jointFitHb_C_variegatus.pdf]

openGUTS Report

Project:

calibration_jointFitHb

Project file:

No project file saved or loaded

Project description (optional):

No project description available

Software version:

openGUTS - 1.0

Date of report creation:

30/05/2020 08:59:40

Calibration

Calibration input data

Data set 1

File: openGUTSInput_1781

Description (optional):

Control group: 'acute 0 µg a.s./L'

Survival data of input data set 1:

Time [d]	acute 0 µg a.s./L	acute 13.0 µg a.s./L	acute 25.0 µg a.s./L	acute 50.0 µg a.s./L	acute 100 µg a.s./L	acute 200 µg a.s./L
0	14	7	7	7	7	7
1	14	7	7	7	5	0
2	14	7	7	7	0	0
3	14	7	7	7	0	0
4	14	7	7	2	0	0

Concentration data of input data set 1:

Time [d]	acute 0 µg a.s./L	acute 13.0 µg a.s./L	acute 25.0 µg a.s./L	acute 50.0 µg a.s./L	acute 100 µg a.s./L	acute 200 µg a.s./L
0	0	8.7	16	32	70	150

Calibration settings

Calibration parameter settings for GUTS-RED-SD:

Parameter	Fit	Min	Max	Scale
kd	Yes	0.001641	16.06	Log
mw	Yes	0.00238	89.59	Log
hb	Yes	1E-6	0.07	Norm
bw	Yes	0.01578	2230	Log
Fs	No	1	1	Norm

Calibration parameter settings for GUTS-RED-IT:

Parameter	Fit	Min	Max	Scale
kd	Yes	0.001641	6.924	Log
mw	Yes	0.00238	108.7	Log
hb	Yes	1E-6	0.07	Norm
bw	No	Inf	Inf	Norm
Fs	Yes	1.05	20	Log

Calibration results

Fitted parameters for GUTS-RED-SD:

Best fit parameter values and their 95% CI

kd: 0.5114 (0.4354 - 0.805)

mw: 27.61 (22.15 - 28.64)

hb: 1E-6 (1E-6* - 0.01444)

bw: 84.2 (0.2102 - 2230*)

* edge of 95% parameter CI has run into a boundary

(this may also affect CIs of other parameters)

Goodness of fit for calibration data (GUTS-RED-SD):

Model efficiency (NSE, r-square): 1

Normalised root-means-square error (NRMSE): 0.0006404 %

Minus log-likelihood (MLL): 8.38

AIC: 24.75

Survival probability prediction error (SPPE) for each treatment:

Data set	Treatment	Value
1	acute 0 µg a.s./L	0.0004 %
1	acute 13.0 µg a.s./L	0.0004 %
1	acute 25.0 µg a.s./L	0.0004 %
1	acute 50.0 µg a.s./L	0.001977 %
1	acute 100 µg a.s./L	0 %
1	acute 200 µg a.s./L	0 %

GUTS-RED-SD results table for LCx,t [[C]], with 95% CI:

Time [d]	LC50	LC20	LC10
1	70.46 (67.39 - 89.47)	69.81 (60.31 - 76.27)	69.54 (56.94 - 72.12)
2	43.68 (41.29 - 48.33)	43.43 (38.44 - 43.9)	43.33 (37.03 - 43.63)
3	35.52 (33.67 - 37.01)	35.38 (31.6 - 35.56)	35.32 (30.66 - 35.55)
4	31.92 (29.48 - 32.81)	31.83 (27.91 - 31.99)	31.79 (27.3 - 31.98)
7	28.49 (24.38 - 29.65)	28.45 (23.7 - 29.2)	28.43 (23.44 - 29.05)
14	27.64 (22.38 - 28.85)	27.64 (22.17 - 28.71)	27.64 (22.1 - 28.67)
21	27.61 (22.11 - 28.73)	27.61 (22.01 - 28.66)	27.61 (21.98 - 28.65)
28	27.61 (22.04 - 28.69)	27.61 (21.97 - 28.65)	27.61 (21.95 - 28.64)

42	27.61 (21.99 - 28.66)	27.61 (21.95 - 28.64)	27.61 (21.94 - 28.63)
50	27.61 (21.98 - 28.66)	27.61 (21.95 - 28.64)	27.61 (21.94 - 28.63)
100	27.61 (21.95 - 28.64)	27.61 (21.94 - 28.63)	27.61 (21.94 - 28.63)

Plots for GUTS-RED-SD calibration:

Parameter space plot for the calibration of GUTS-RED-SD:

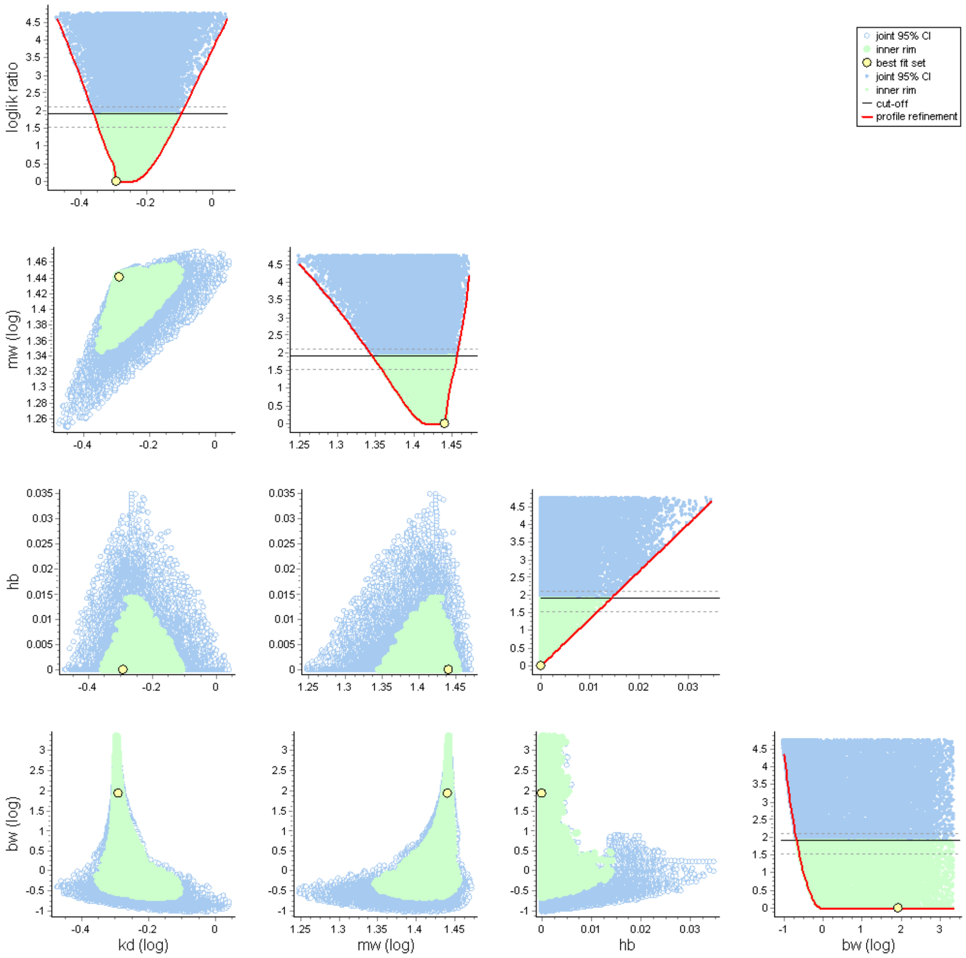

Exposure, damage and survival plots for the calibration of GUTS-RED-SD:

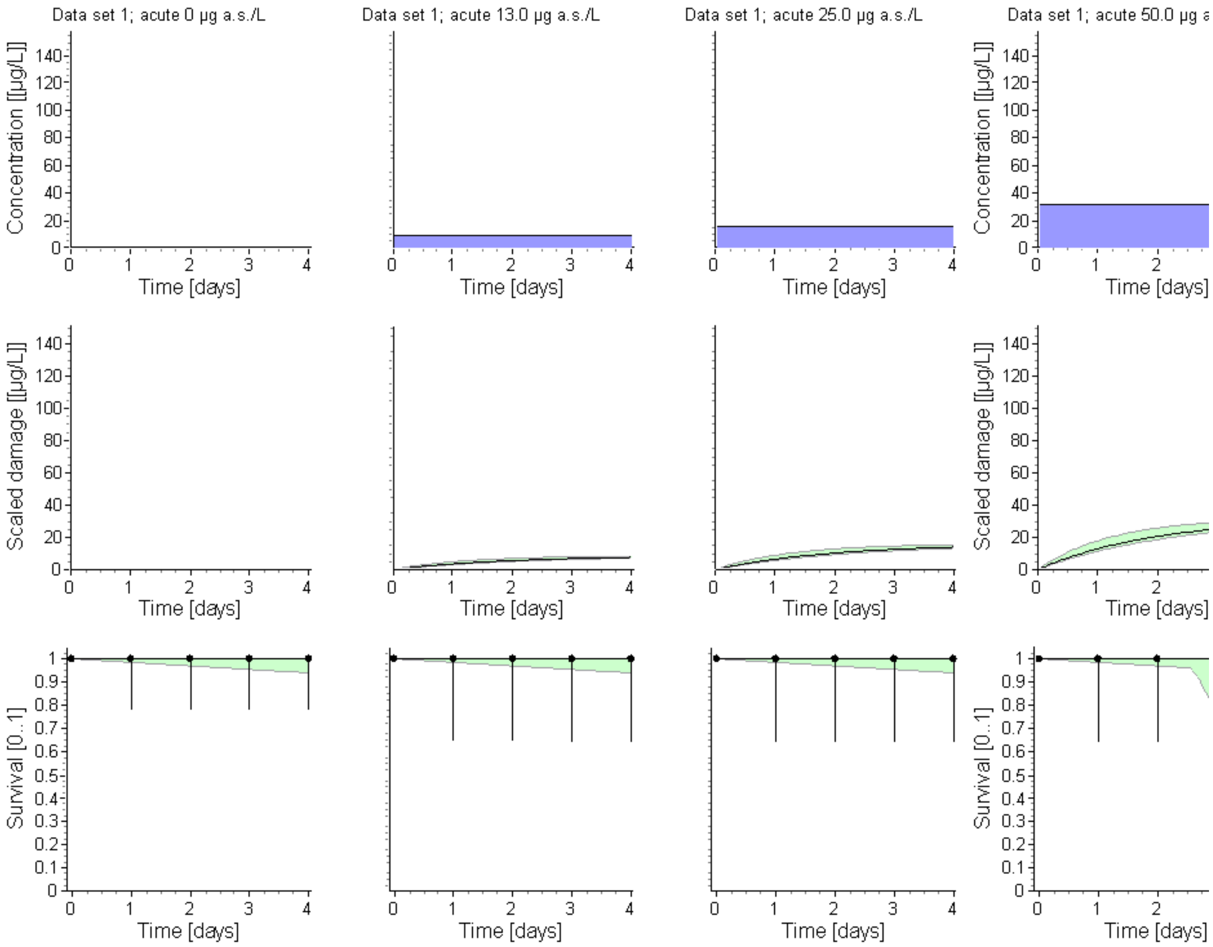

... continued plot:

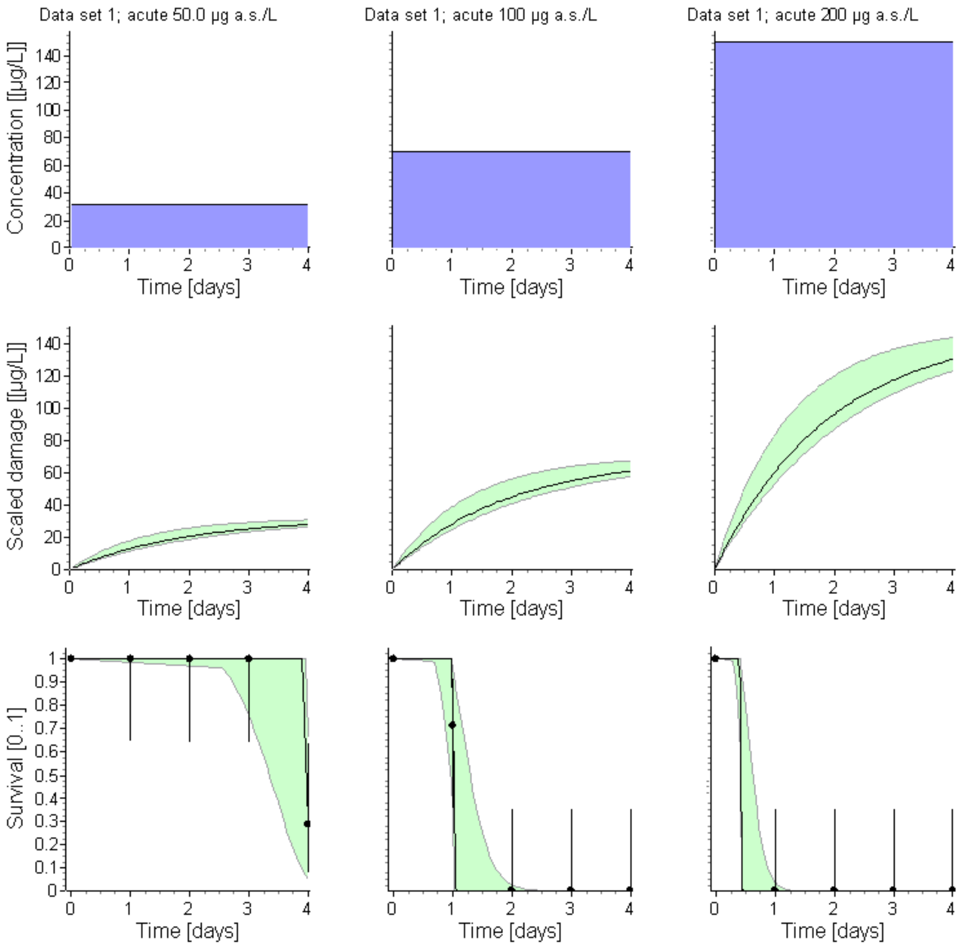

Observed vs. Predicted survival plot for the calibration of GUTS-RED-SD:

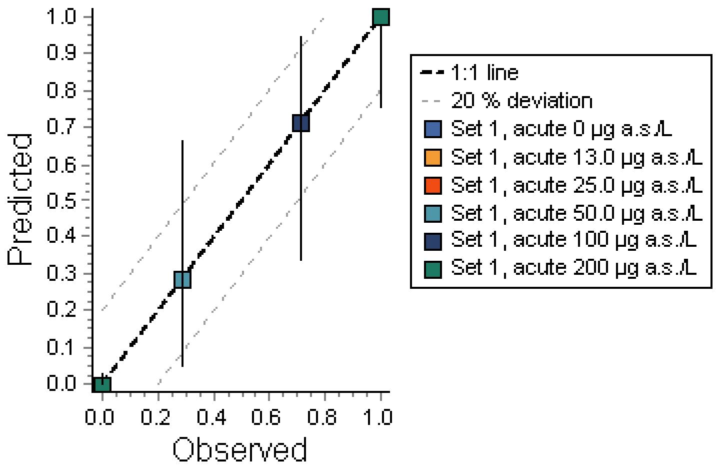

Observed vs. Predicted deaths plot for the calibration of GUTS-RED-SD:

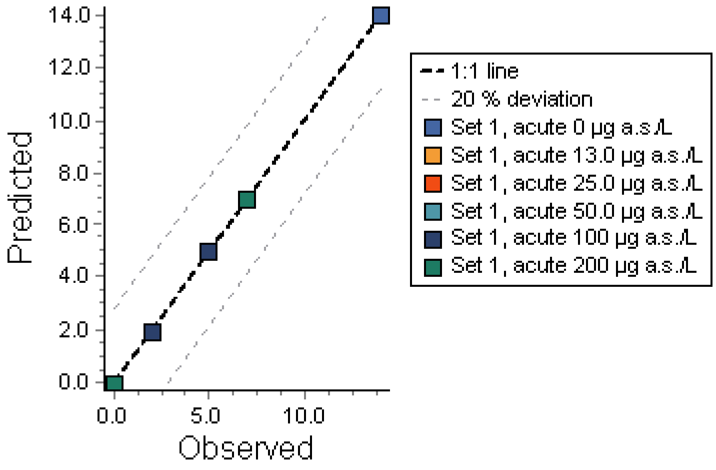

LCx versus time with confidence intervals (plotted for 16 days, GUTS-RED-SD):

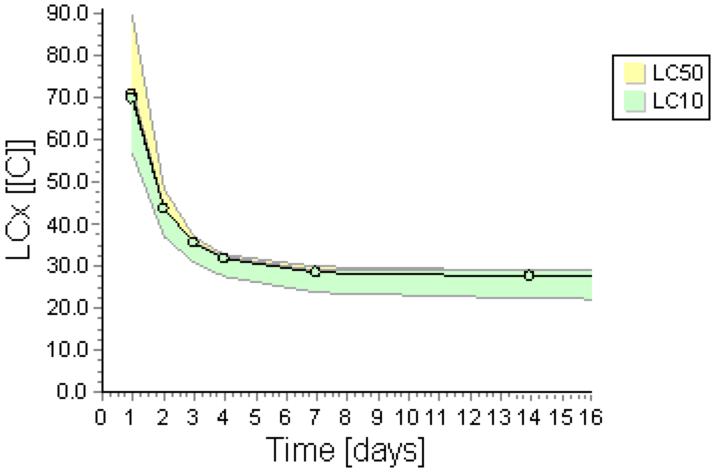

LC50
LC10
LCx [[C]]
Time [days]
90.0
80.0
70.0
60.0
50.0
40.0
30.0
20.0
10.0
0.0
0 1 2 3 4 5 6 7 8 9 10 11 12 13 14 15 16

Fitted parameters for GUTS-RED-IT:

Best fit parameter values and their 95% CI

kd: 0.4786 (0.3333 - 0.5136)

mw: 26.95 (22.02 - 28.17)

hb: 1E-6 (1E-6* - 0.01444)

Fs: 1.05 (1.05* - 1.286)

* edge of 95% parameter CI has run into a boundary

(this may also affect CIs of other parameters)

Goodness of fit for calibration data (GUTS-RED-IT):

Model efficiency (NSE, r-square): 1

Normalised root-means-square error (NRMSE): 0.01535 %

Minus log-likelihood (MLL): 8.38

AIC: 24.76

Survival probability prediction error (SPPE) for each treatment:

Data set	Treatment	Value
1	acute 0 µg a.s./L	0.0004001 %
1	acute 13.0 µg a.s./L	0.0004001 %
1	acute 25.0 µg a.s./L	0.0004001 %
1	acute 50.0 µg a.s./L	-0.02 %
1	acute 100 µg a.s./L	0 %
1	acute 200 µg a.s./L	0 %

GUTS-RED-IT results table for LCx,t [[C]], with 95% CI:

Time [d]	LC50	LC20	LC10
1	70.86 (69.11 - 79.64)	69.56 (65.47 - 73.77)	68.82 (62.87 - 71.37)
2	43.75 (42.62 - 46.63)	42.95 (39.98 - 43.76)	42.49 (38 - 43.2)
3	35.37 (33.81 - 36.6)	34.72 (31.37 - 35.26)	34.35 (29.76 - 34.88)
4	31.61 (29.38 - 32.54)	31.03 (27.07 - 31.6)	30.7 (25.63 - 31.26)
7	27.93 (24.01 - 29.01)	27.42 (22.13 - 28.23)	27.13 (20.94 - 27.93)
14	26.98 (21.84 - 28.21)	26.49 (20.13 - 27.44)	26.21 (19.08 - 27.14)
21	26.95 (21.65 - 28.18)	26.46 (19.93 - 27.42)	26.18 (18.92 - 27.12)
28	26.95 (21.63 - 28.18)	26.46 (19.91 - 27.42)	26.17 (18.9 - 27.12)
42	26.95 (21.63 - 28.18)	26.46 (19.91 - 27.42)	26.17 (18.9 - 27.12)

50	26.95 (21.63 - 28.18)	26.46 (19.91 - 27.42)	26.17 (18.9 - 27.12)
100	26.95 (21.63 - 28.18)	26.46 (19.91 - 27.42)	26.17 (18.9 - 27.12)

Plots for GUTS-RED-IT calibration:

Parameter space plot for the calibration of GUTS-RED-IT:

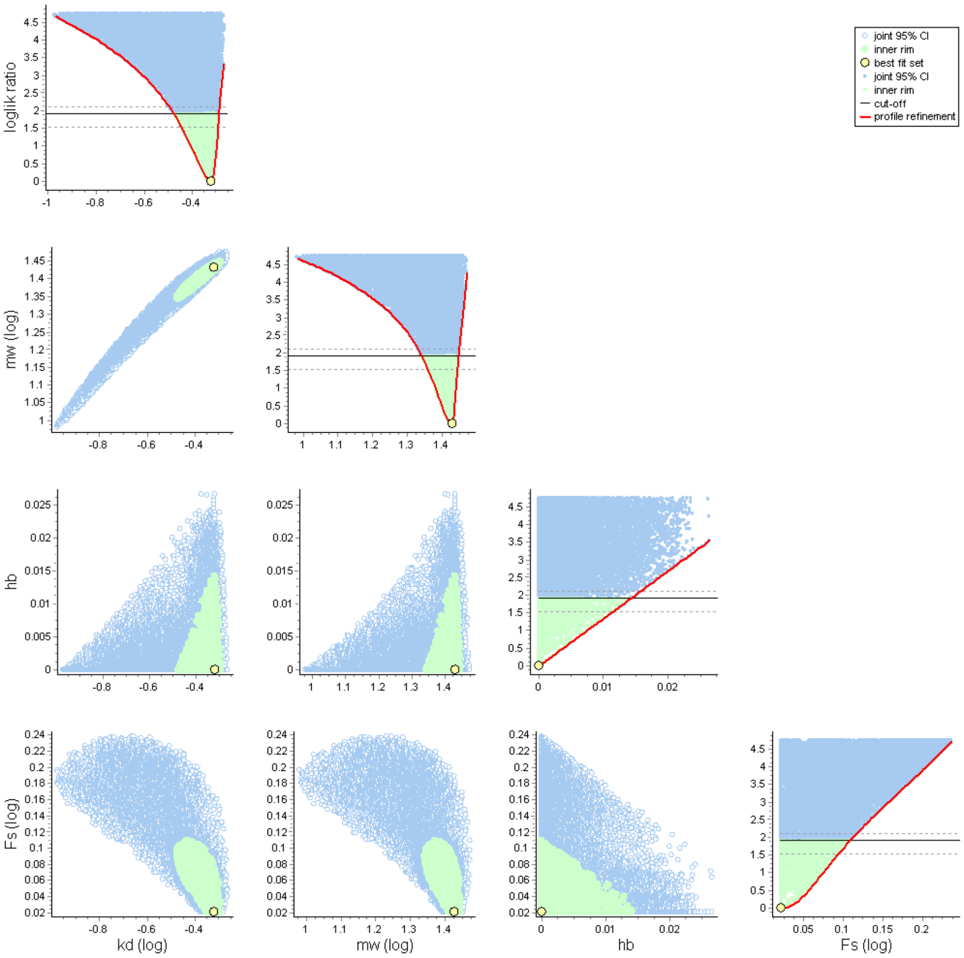

Exposure, damage and survival plots for the calibration of GUTS-RED-IT:

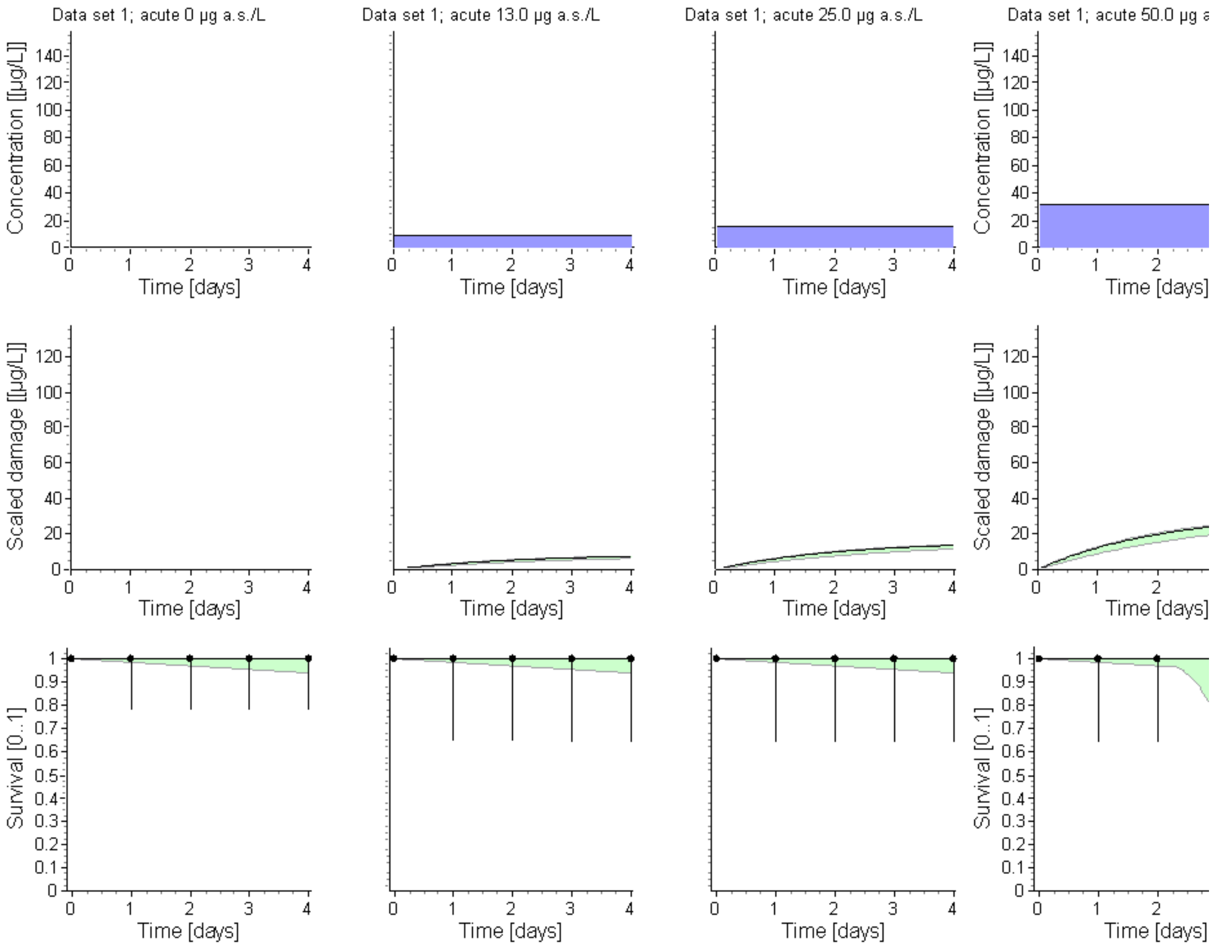

... continued plot:

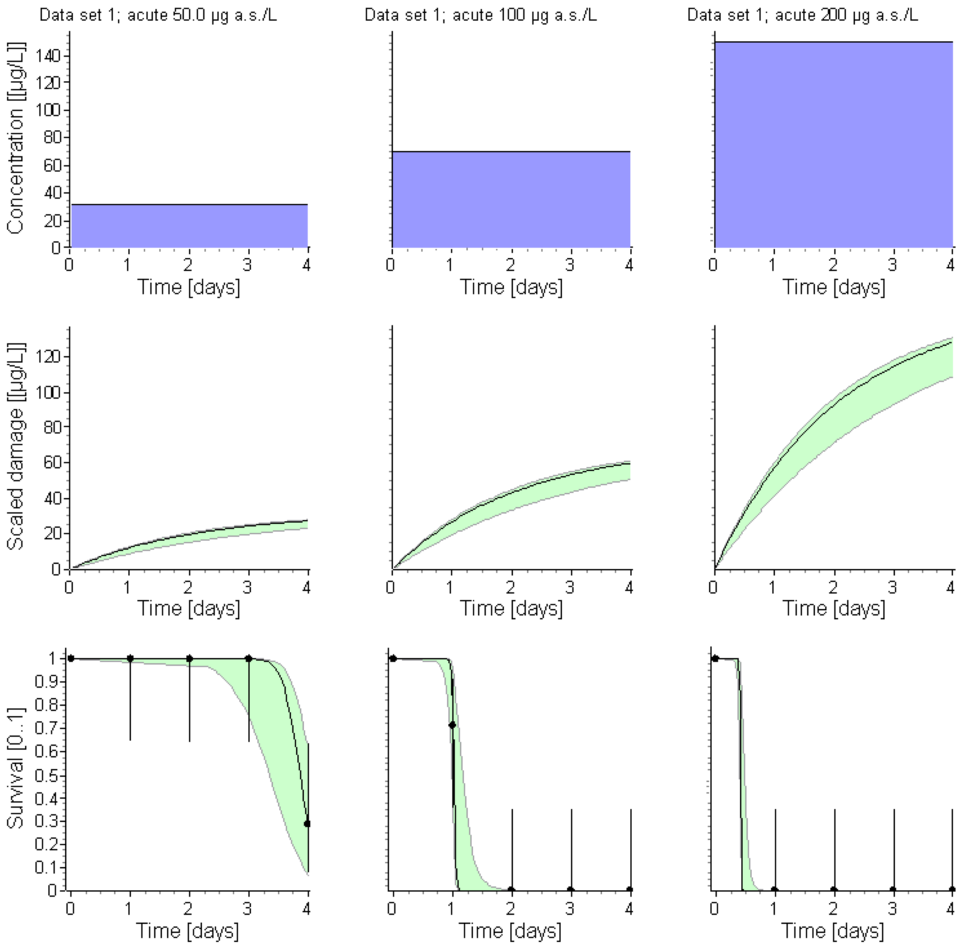

Observed vs. Predicted survival plot for the calibration of GUTS-RED-IT:

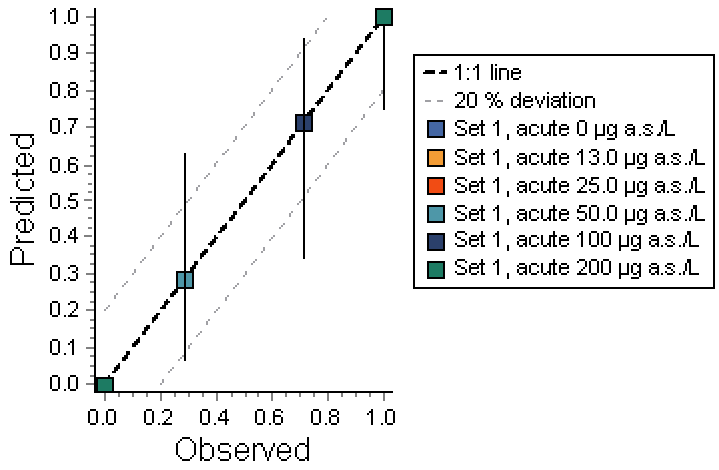

Observed vs. Predicted deaths plot for the calibration of GUTS-RED-IT:

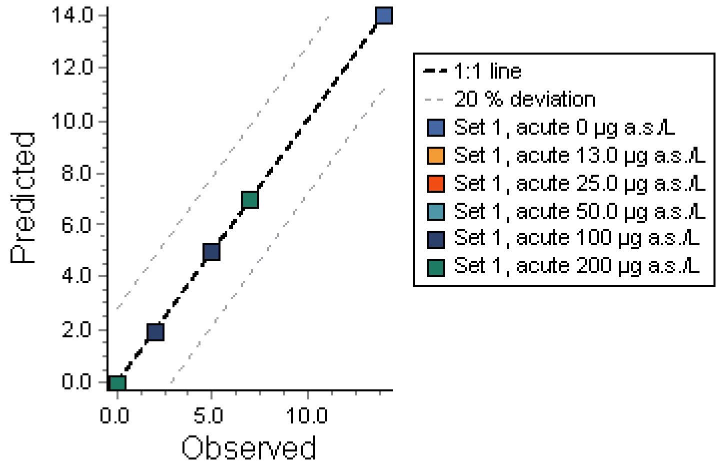

LCx versus time with confidence intervals (plotted for 16 days, GUTS-RED-IT):

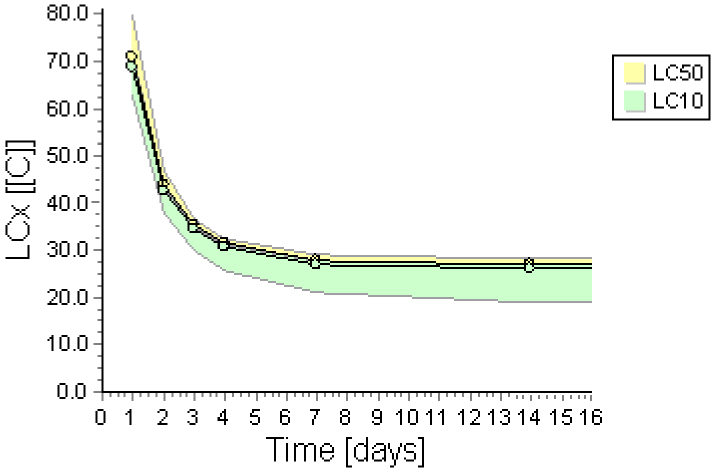

LC50
LC10
LCx [[C]]
Time [days]
80.0
70.0
60.0
50.0
40.0
30.0
20.0
10.0
0.0
0 1 2 3 4 5 6 7 8 9 10 11 12 13 14 15 16

Validation

No validation performed!

Predictions

No predictions performed!
