# Supplementary material for: Fish Species Sensitivity Ranking Depends on Pesticide Exposure Profiles
Source: Environ Toxicol Chem. 2022 Jun 6;41(7):1732–41. doi: 10.1002/etc.5348 (PMC9328144; doi:10.1002/etc.5348)
Supplement: Supplementary file 2 — Supporting information. [file ETC-41-1732-s004.zip › fits openGUTS standalone/calibration_jointFitHb_L_macrochirus.pdf]

openGUTS Report

Project:

calibration_jointFitHb

Project file:

No project file saved or loaded

Project description (optional):

No project description available

Software version:

openGUTS - 1.0

Date of report creation:

30/05/2020 08:22:20

Calibration

Calibration input data

Data set 1

File: openGUTSInput_1781

Description (optional):

Control group: 'acute 0 µg a.s./L'

Survival data of input data set 1:

Time [d]	acute 0 µg a.s./L	acute 6.30 µg a.s./L	acute 13.0 µg a.s./L	acute 25.0 µg a.s./L	acute 50.0 µg a.s./L	acute 100 µg a.s./L
0	14	7	7	7	7	7
1	14	7	7	7	2	0
2	14	7	7	6	0	0
3	14	7	7	5	0	0
4	14	7	7	5	0	0

Concentration data of input data set 1:

Time [d]	acute 0 µg a.s./L	acute 6.30 µg a.s./L	acute 13.0 µg a.s./L	acute 25.0 µg a.s./L	acute 50.0 µg a.s./L	acute 100 µg a.s./L
0	0	5.7	11	23	47	90

Calibration settings

Calibration parameter settings for GUTS-RED-SD:

Parameter	Fit	Min	Max	Scale
kd	Yes	0.001641	143.8	Log
mw	Yes	0.001559	89.1	Norm
hb	Yes	1E-6	0.07	Norm
bw	Yes	0.0002927	3716	Log
Fs	No	1	1	Norm

Calibration parameter settings for GUTS-RED-IT:

Parameter	Fit	Min	Max	Scale
kd	Yes	0.001641	143.8	Log
mw	Yes	0.001559	180	Norm
hb	Yes	1E-6	0.07	Norm
bw	No	Inf	Inf	Norm
Fs	Yes	1.05	20	Log

Calibration results

Fitted parameters for GUTS-RED-SD:

Best fit parameter values and their 95% CI

kd: 3.167 (1.447 - 143.8*)

mw: 21.81 (18.68 - 22.75)

hb: 10E-7 (1E-6* - 0.01618)

bw: 0.1116 (0.03819 - 0.2903)

* edge of 95% parameter CI has run into a boundary

(this may also affect CIs of other parameters)

Goodness of fit for calibration data (GUTS-RED-SD):

Model efficiency (NSE, r-square): 0.9976

Normalised root-means-square error (NRMSE): 2.677 %

Minus log-likelihood (MLL): 10.8

AIC: 29.61

Survival probability prediction error (SPPE) for each treatment:

Data set	Treatment	Value
1	acute 0 µg a.s./L	0.0004 %
1	acute 6.30 µg a.s./L	0.0004 %
1	acute 13.0 µg a.s./L	0.0004 %
1	acute 25.0 µg a.s./L	2.018 %
1	acute 50.0 µg a.s./L	-0.01 %
1	acute 100 µg a.s./L	-1.29E-10 %

GUTS-RED-SD results table for LCx,t [[C]], with 95% CI:

Time [d]	LC50	LC20	LC10
1	36.32 (28.57 - 48.48)	28.71 (23.43 - 35.78)	26.37 (21.26 - 32.38)
2	27.11 (24.85 - 31.48)	23.96 (21.27 - 25.68)	23.04 (19.98 - 24.34)
3	24.88 (22.83 - 27.75)	22.96 (20.21 - 24.16)	22.42 (19.41 - 23.37)
4	23.94 (21.67 - 26.18)	22.57 (19.71 - 23.67)	22.2 (19.14 - 23.15)
7	22.9 (20.3 - 24.43)	22.18 (19.16 - 23.2)	21.99 (18.85 - 22.95)
14	22.31 (19.4 - 23.42)	21.97 (18.84 - 22.95)	21.89 (18.69 - 22.85)
21	22.13 (19.11 - 23.18)	21.92 (18.74 - 22.88)	21.86 (18.63 - 22.82)
28	22.05 (18.97 - 23.06)	21.89 (18.69 - 22.85)	21.85 (18.61 - 22.81)

42	21.96 (18.83 - 22.95)	21.86 (18.63 - 22.82)	21.83 (18.58 - 22.8)
50	21.94 (18.78 - 22.91)	21.85 (18.62 - 22.82)	21.83 (18.58 - 22.79)
100	21.87 (18.66 - 22.84)	21.83 (18.58 - 22.79)	21.82 (18.56 - 22.78)

<u>Plots for GUTS-RED-SD calibration:</u>

Parameter space plot for the calibration of GUTS-RED-SD:

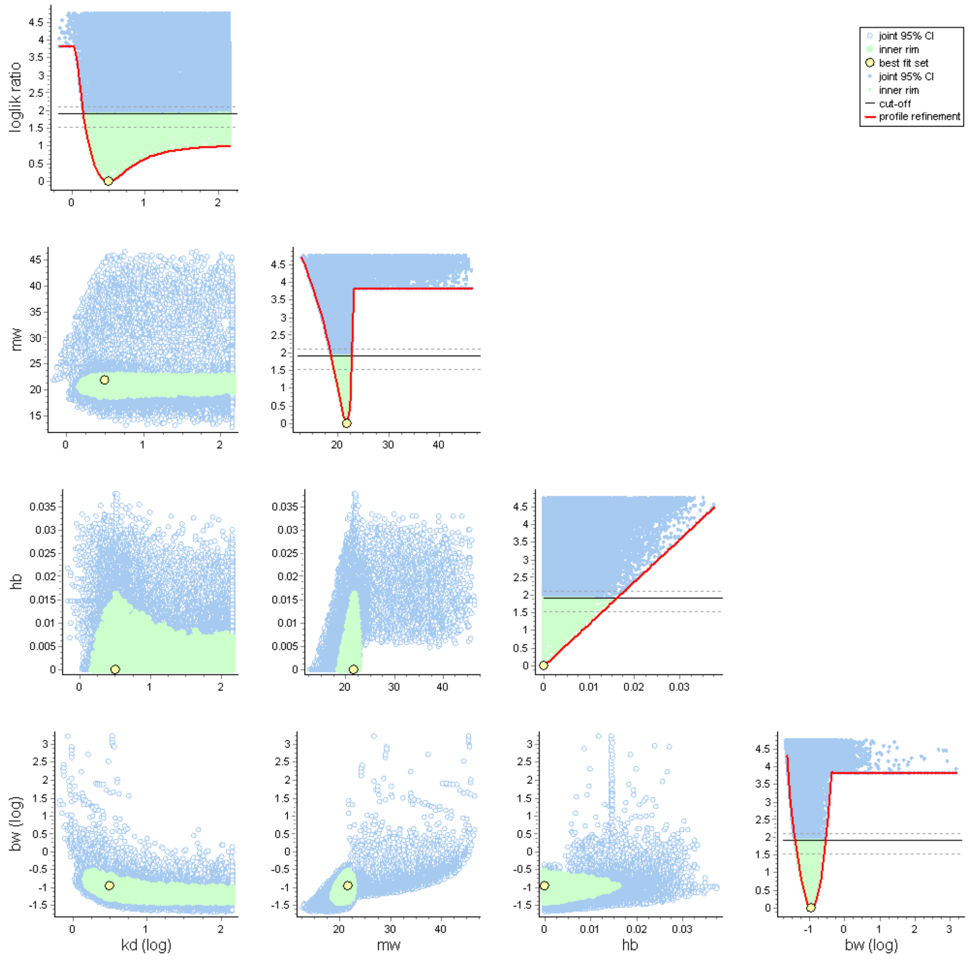

Exposure, damage and survival plots for the calibration of GUTS-RED-SD:

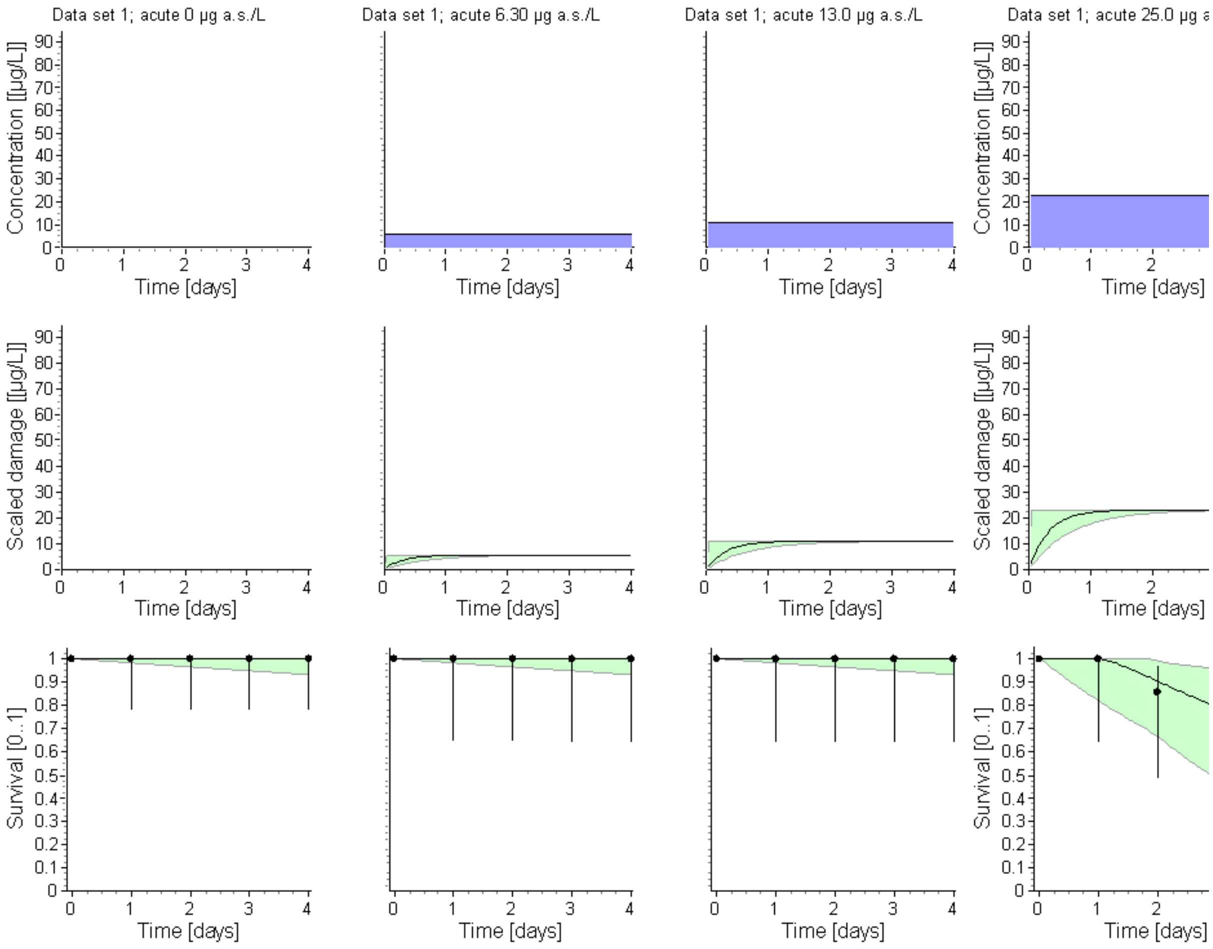

... continued plot:

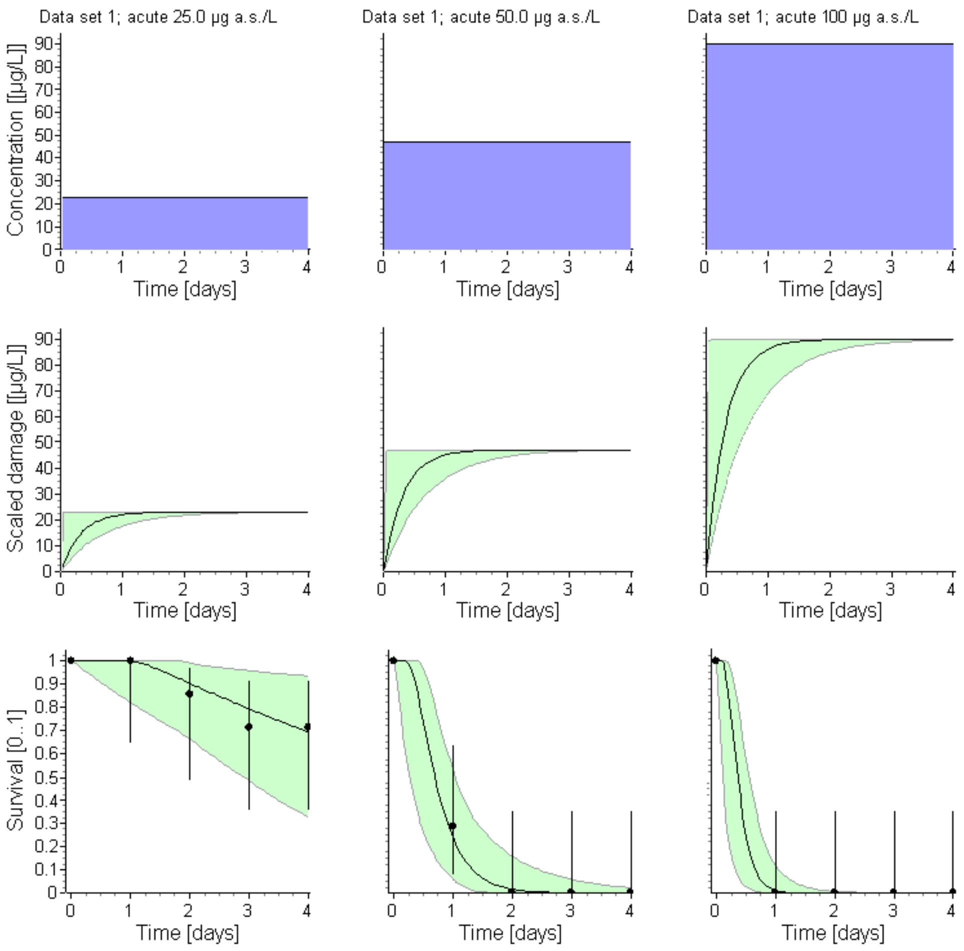

Observed vs. Predicted survival plot for the calibration of GUTS-RED-SD:

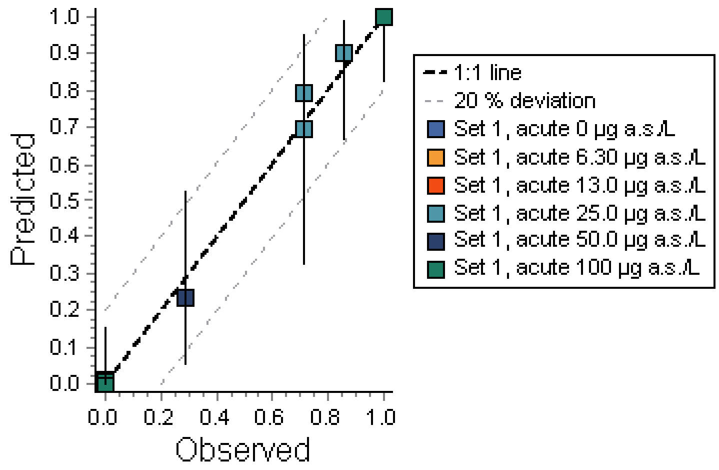

Observed vs. Predicted deaths plot for the calibration of GUTS-RED-SD:

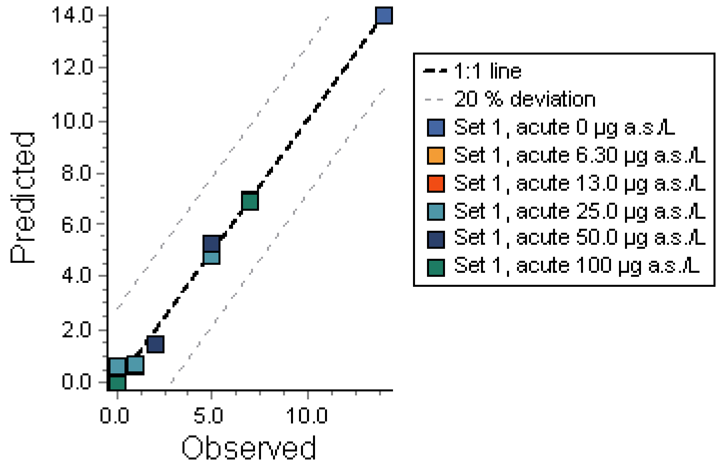

LCx versus time with confidence intervals (plotted for 16 days, GUTS-RED-SD):

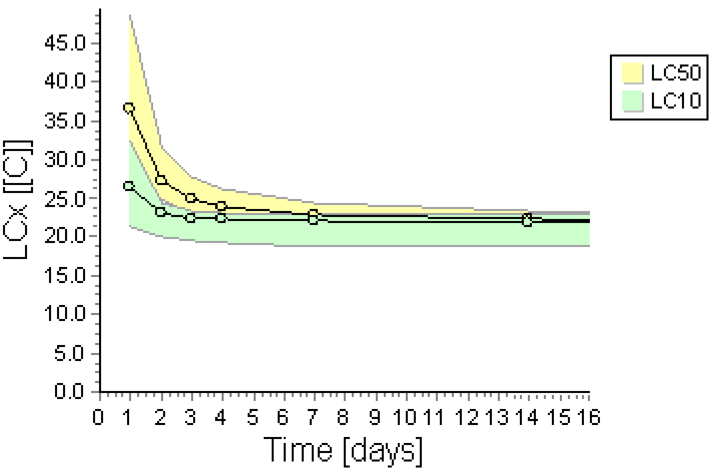

LC50
LC10
LCx [[C]]
Time [days]
0.0
5.0
10.0
15.0
20.0
25.0
30.0
35.0
40.0
45.0
0 1 2 3 4 5 6 7 8 9 10 11 12 13 14 15 16

Fitted parameters for GUTS-RED-IT:

Best fit parameter values and their 95% CI

kd: 0.9403 (0.5585 - 1.642)

mw: 25.18 (19.26 - 32.42)

hb: 1E-6 (1E-6* - 0.0161)

Fs: 1.559 (1.241 - 2.524)

* edge of 95% parameter CI has run into a boundary

(this may also affect CIs of other parameters)

Goodness of fit for calibration data (GUTS-RED-IT):

Model efficiency (NSE, r-square): 0.9985

Normalised root-means-square error (NRMSE): 2.078 %

Minus log-likelihood (MLL): 10.52

AIC: 29.04

Survival probability prediction error (SPPE) for each treatment:

Data set	Treatment	Value
1	acute 0 µg a.s./L	0.0004 %
1	acute 6.30 µg a.s./L	0.0007895 %
1	acute 13.0 µg a.s./L	0.0888 %
1	acute 25.0 µg a.s./L	-0.54 %
1	acute 50.0 µg a.s./L	-0.7 %
1	acute 100 µg a.s./L	0 %

GUTS-RED-IT results table for LCx,t [[C]], with 95% CI:

Time [d]	LC50	LC20	LC10
1	41.32 (33.2 - 50.12)	34.93 (25.4 - 41.95)	31.66 (21.19 - 39.15)
2	29.72 (25.11 - 35.48)	25.12 (19.02 - 29.55)	22.77 (15.75 - 27.35)
3	26.78 (22.34 - 33.33)	22.64 (16.98 - 27.62)	20.52 (14.08 - 25.14)
4	25.78 (20.94 - 32.88)	21.8 (16.07 - 27.18)	19.76 (13.37 - 24.76)
7	25.22 (19.47 - 32.76)	21.32 (15.08 - 27.01)	19.32 (12.75 - 24.66)
14	25.18 (19.16 - 32.75)	21.29 (14.9 - 27.01)	19.3 (12.64 - 24.66)
21	25.18 (19.15 - 32.75)	21.29 (14.9 - 27.01)	19.3 (12.64 - 24.66)
28	25.18 (19.15 - 32.75)	21.29 (14.9 - 27.01)	19.3 (12.64 - 24.66)
42	25.18 (19.15 - 32.75)	21.29 (14.9 - 27.01)	19.3 (12.64 - 24.66)

50	25.18 (19.15 - 32.75)	21.29 (14.9 - 27.01)	19.3 (12.64 - 24.66)
100	25.18 (19.15 - 32.75)	21.29 (14.9 - 27.01)	19.3 (12.64 - 24.66)

Plots for GUTS-RED-IT calibration:

Parameter space plot for the calibration of GUTS-RED-IT:

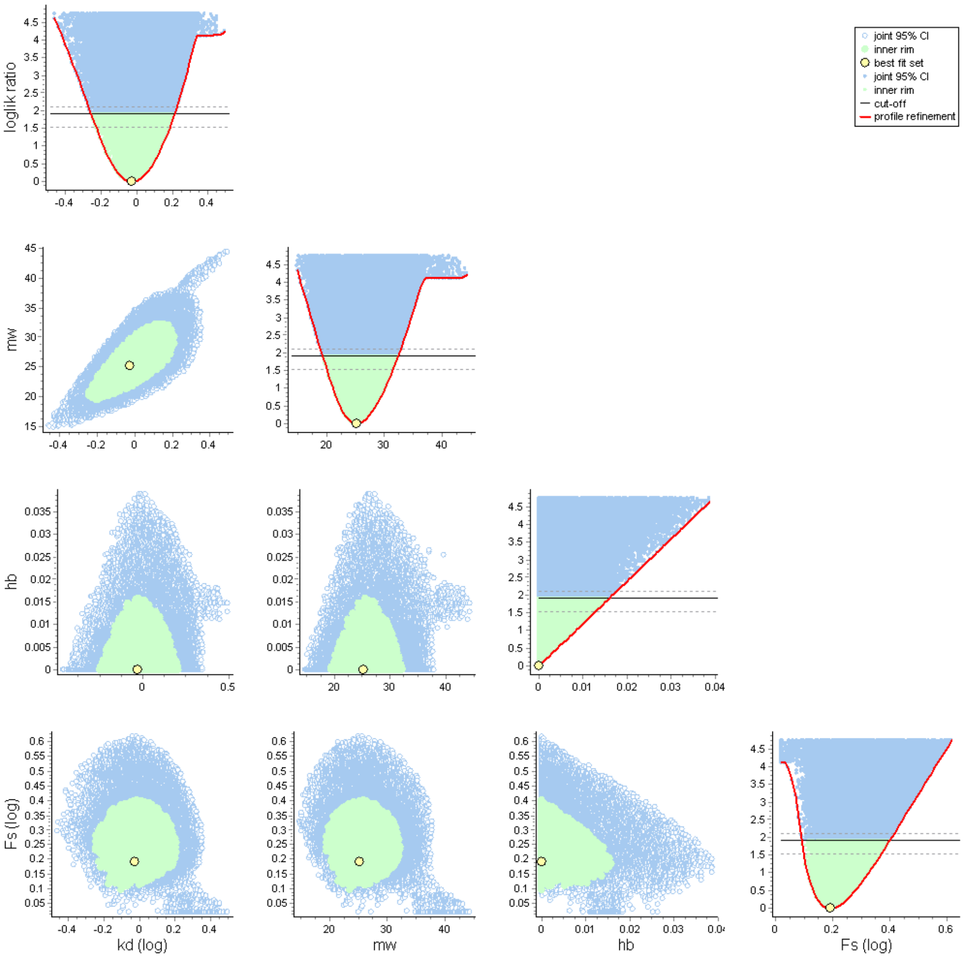

Exposure, damage and survival plots for the calibration of GUTS-RED-IT:

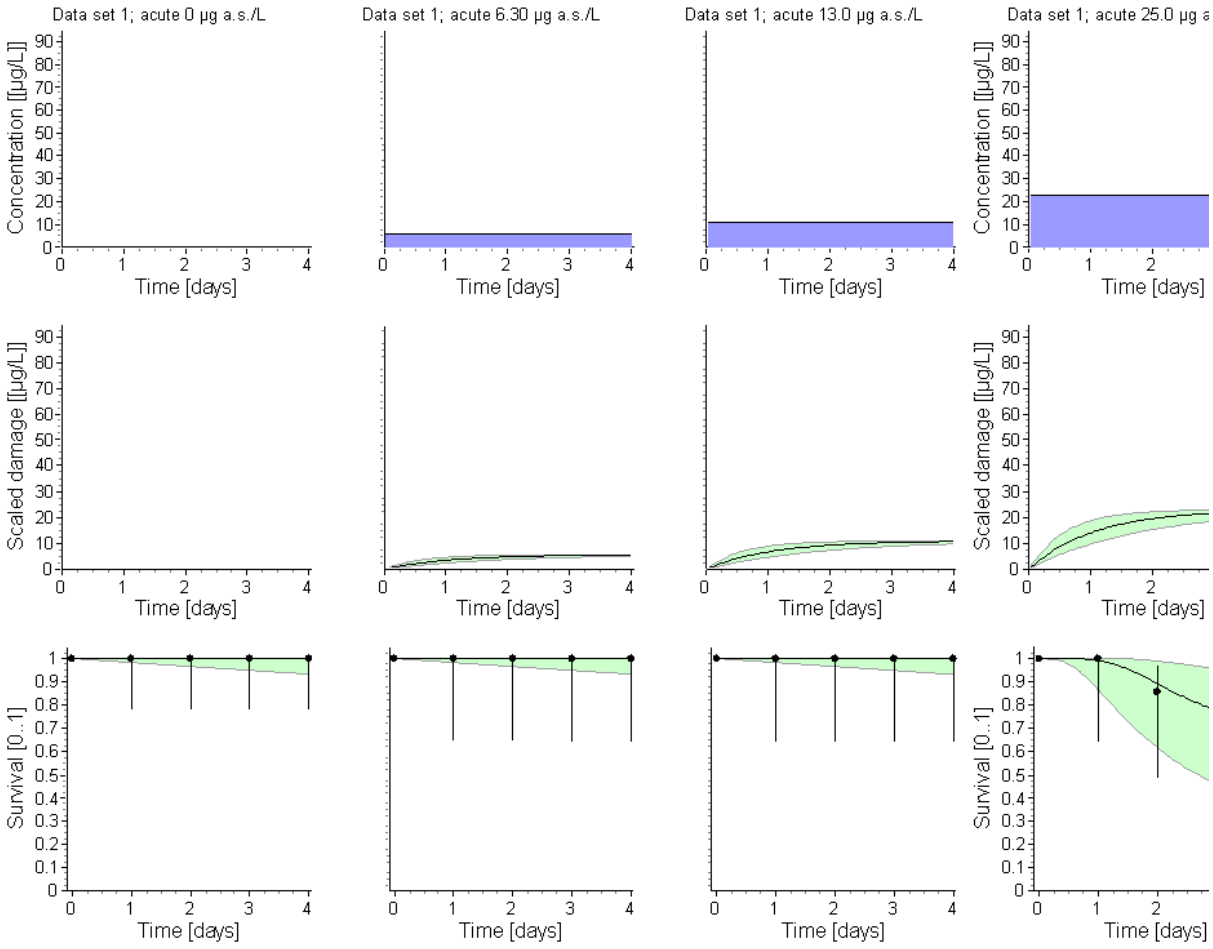

... continued plot:

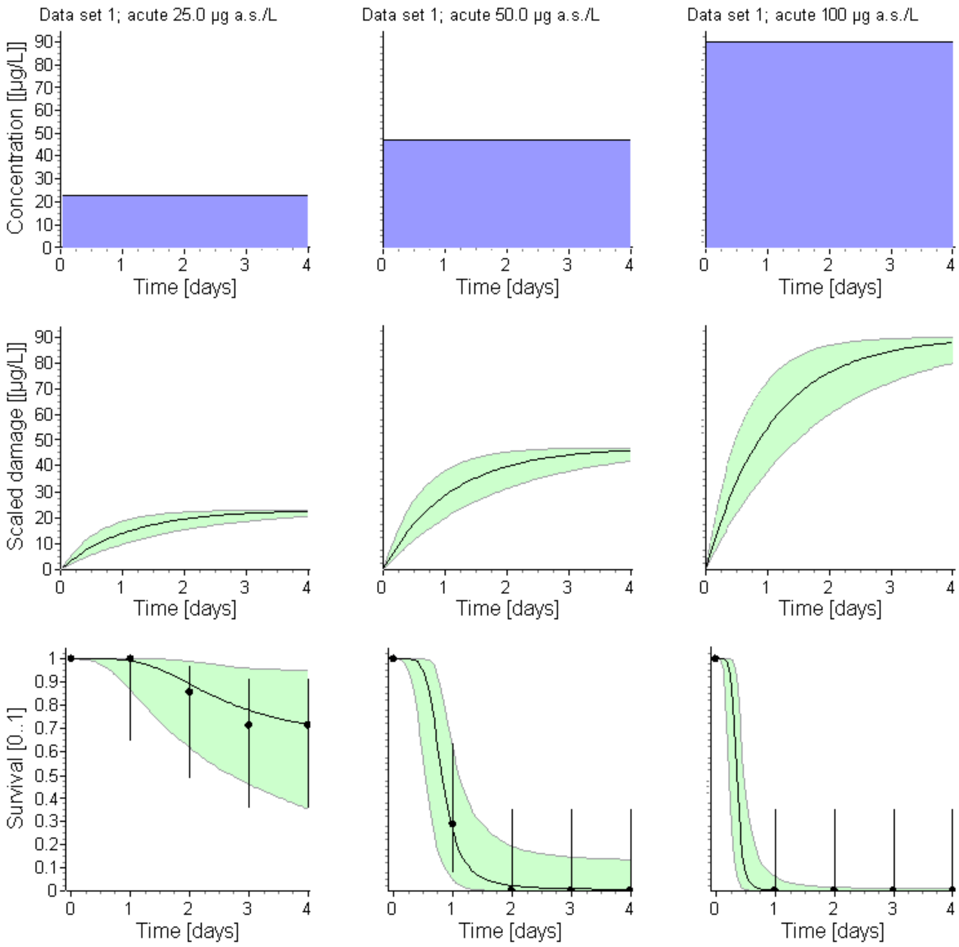

Observed vs. Predicted survival plot for the calibration of GUTS-RED-IT:

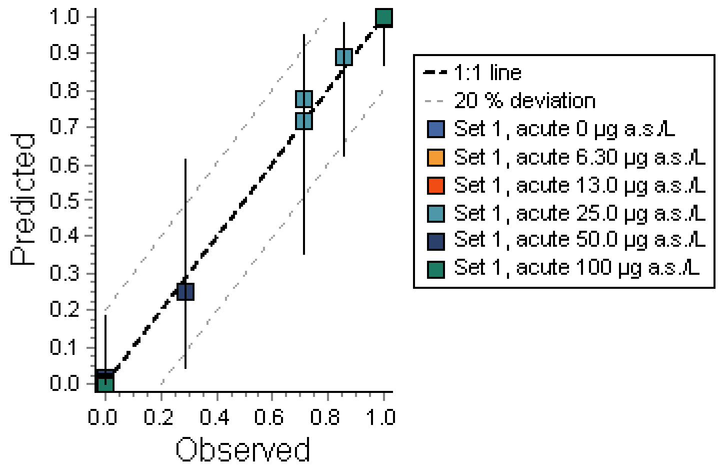

Observed vs. Predicted deaths plot for the calibration of GUTS-RED-IT:

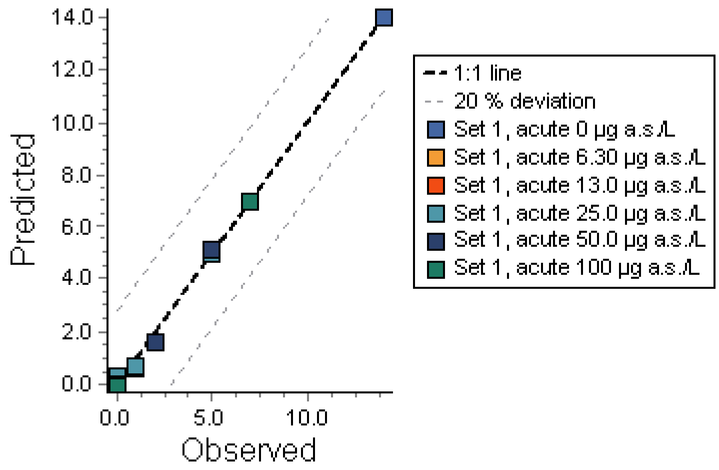

LCx versus time with confidence intervals (plotted for 16 days, GUTS-RED-IT):

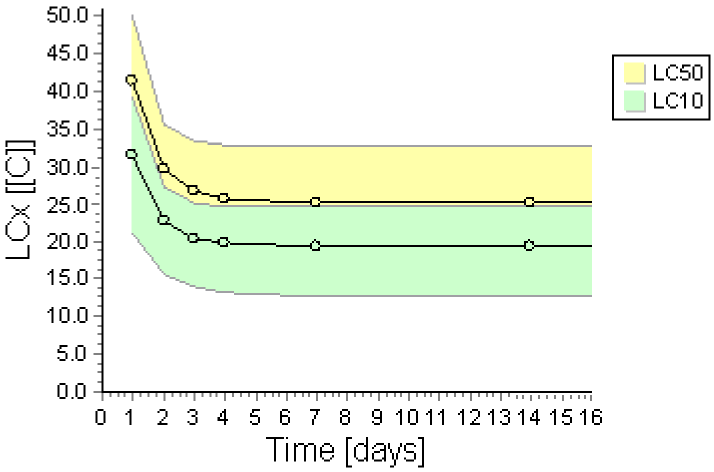

LCx [[C]]
Time [days]
LC50
LC10

Validation

No validation performed!

Predictions

No predictions performed!
