# Supplementary material for: Fish Species Sensitivity Ranking Depends on Pesticide Exposure Profiles
Source: Environ Toxicol Chem. 2022 Jun 6;41(7):1732–41. doi: 10.1002/etc.5348 (PMC9328144; doi:10.1002/etc.5348)
Supplement: Supplementary file 2 — Supporting information. [file ETC-41-1732-s004.zip › fits openGUTS standalone/calibration_jointFitHb_O_mykiss.pdf]

openGUTS Report

Project:

calibration_jointFitHb

Project file:

No project file saved or loaded

Project description (optional):

No project description available

Software version:

openGUTS - 1.0

Date of report creation:

30/05/2020 08:31:26

Calibration

Calibration input data

Data set 1

File: openGUTSInput_1781

Description (optional):

Control group: 'acute 0 µg a.s./L'

Survival data of input data set 1:

Time [d]	acute 0 µg a.s./L	acute 1.60 µg a.s./L	acute 3.10 µg a.s./L	acute 6.30 µg a.s./L	acute 13.0 µg a.s./L	acute 25.0 µg a.s./L
0	14	7	7	7	7	7
1	14	7	7	7	4	0
2	14	7	7	7	3	0
3	14	7	7	7	3	0
4	14	7	7	7	3	0

Concentration data of input data set 1:

Time [d]	acute 0 µg a.s./L	acute 1.60 µg a.s./L	acute 3.10 µg a.s./L	acute 6.30 µg a.s./L	acute 13.0 µg a.s./L	acute 25.0 µg a.s./L
0	0	1.5	2.3	4.7	9.8	18

Calibration settings

Calibration parameter settings for GUTS-RED-SD:

Parameter	Fit	Min	Max	Scale
kd	Yes	0.001641	143.8	Log
mw	Yes	0.0004103	17.82	Norm
hb	Yes	1E-6	0.07	Norm
bw	Yes	0.001463	18581	Log
Fs	No	1	1	Norm

Calibration parameter settings for GUTS-RED-IT:

Parameter	Fit	Min	Max	Scale
kd	Yes	0.001641	143.8	Log
mw	Yes	0.0004103	36	Norm
hb	Yes	1E-6	0.07	Norm
bw	No	Inf	Inf	Norm
Fs	Yes	1.05	20	Log

Calibration results

Fitted parameters for GUTS-RED-SD:

Best fit parameter values and their 95% CI

kd: 143.8 (6.017 - 143.8*)

mw: 9.528 (7.012 - 9.804)

hb: 1E-6 (1E-6* - 0.01374)

bw: 1 (0.139 - 18581*)

* edge of 95% parameter CI has run into a boundary

(this may also affect CIs of other parameters)

Goodness of fit for calibration data (GUTS-RED-SD):

Model efficiency (NSE, r-square): 0.9801

Normalised root-means-square error (NRMSE): 5.995 %

Minus log-likelihood (MLL): 9.33

AIC: 26.65

Survival probability prediction error (SPPE) for each treatment:

Data set	Treatment	Value
1	acute 0 µg a.s./L	0.0004 %
1	acute 1.60 µg a.s./L	0.0004 %
1	acute 3.10 µg a.s./L	0.0004 %
1	acute 6.30 µg a.s./L	0.0004 %
1	acute 13.0 µg a.s./L	8.899 %
1	acute 25.0 µg a.s./L	-2.12E-13 %

GUTS-RED-SD results table for LCx,t [[C]], with 95% CI:

Time [d]	LC50	LC20	LC10
1	10.24 (9.8 - 13.12)	9.759 (8.18 - 10.28)	9.637 (7.498 - 9.854)
2	9.88 (8.855 - 10.85)	9.642 (7.531 - 9.864)	9.582 (7.14 - 9.8)
3	9.761 (8.207 - 10.26)	9.603 (7.282 - 9.8)	9.564 (7.02 - 9.8)
4	9.703 (7.884 - 10.04)	9.584 (7.158 - 9.8)	9.554 (6.957 - 9.8)
7	9.627 (7.442 - 9.829)	9.56 (6.995 - 9.8)	9.543 (6.876 - 9.8)
14	9.577 (7.115 - 9.8)	9.544 (6.882 - 9.8)	9.535 (6.823 - 9.8)
21	9.561 (7.003 - 9.8)	9.538 (6.844 - 9.8)	9.533 (6.805 - 9.8)
28	9.553 (6.944 - 9.8)	9.536 (6.826 - 9.8)	9.532 (6.796 - 9.8)

42	9.544 (6.886 - 9.8)	9.533 (6.807 - 9.8)	9.53 (6.787 - 9.8)
50	9.542 (6.867 - 9.8)	9.532 (6.801 - 9.8)	9.53 (6.784 - 9.8)
100	9.535 (6.818 - 9.8)	9.53 (6.785 - 9.8)	9.529 (6.777 - 9.8)

Plots for GUTS-RED-SD calibration:

Parameter space plot for the calibration of GUTS-RED-SD:

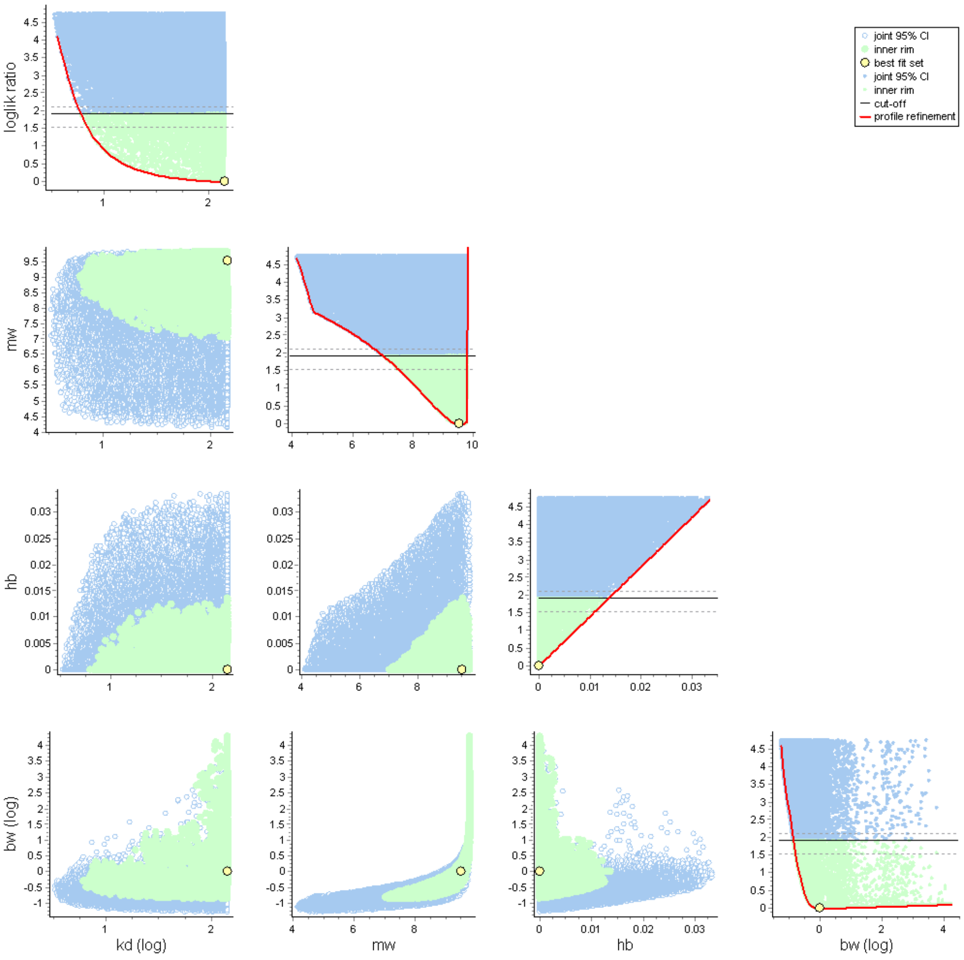

Exposure, damage and survival plots for the calibration of GUTS-RED-SD:

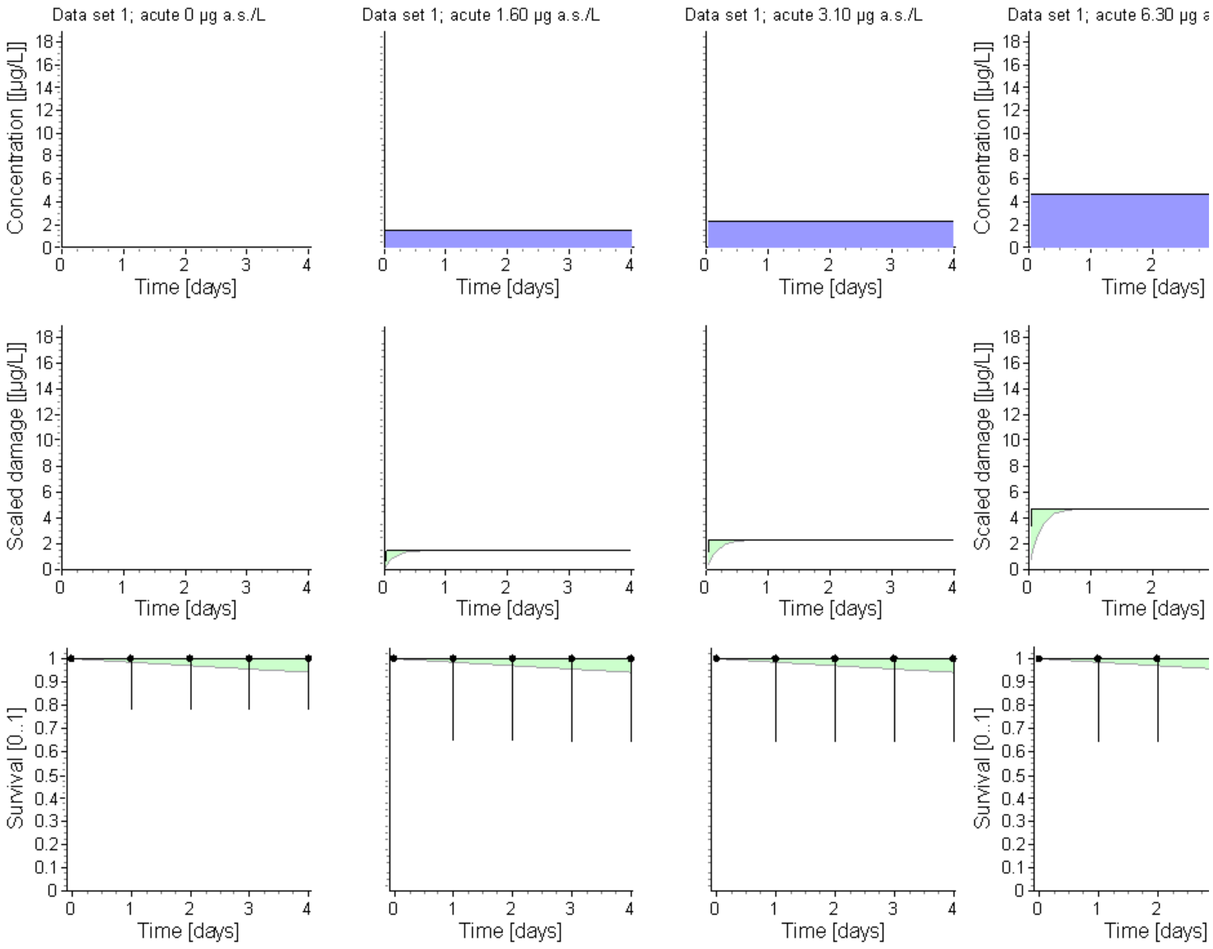

... continued plot:

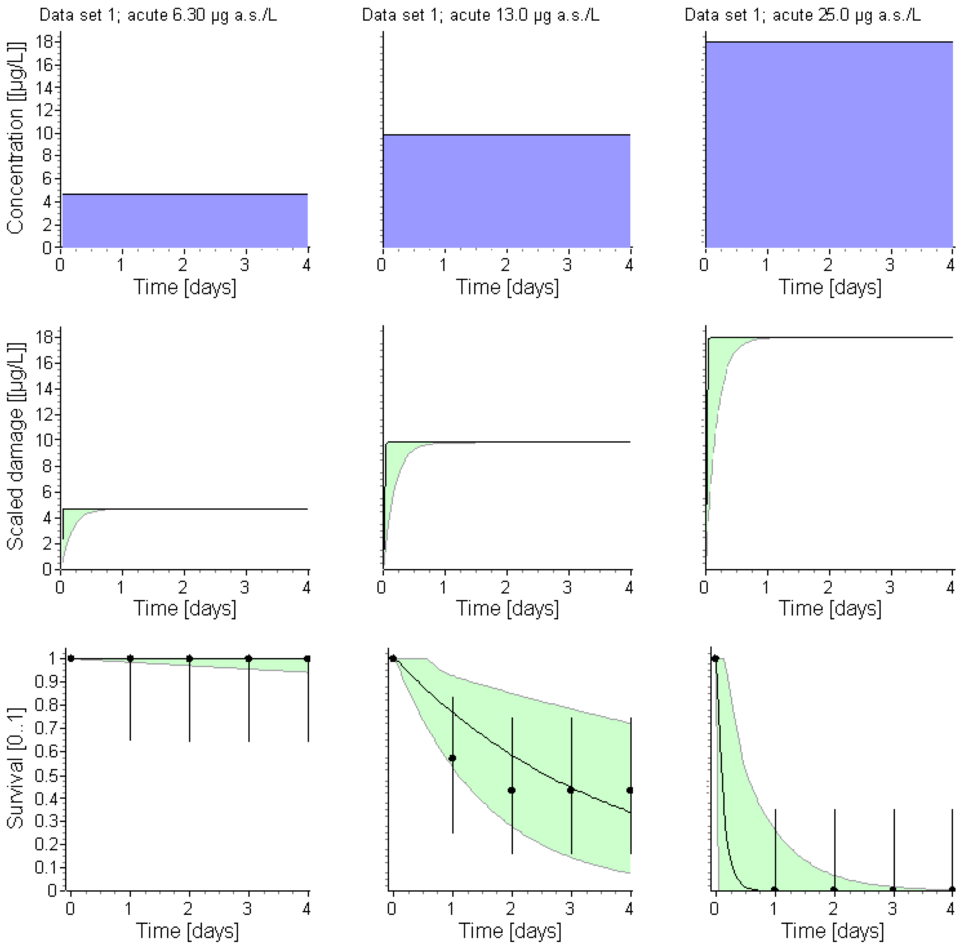

Observed vs. Predicted survival plot for the calibration of GUTS-RED-SD:

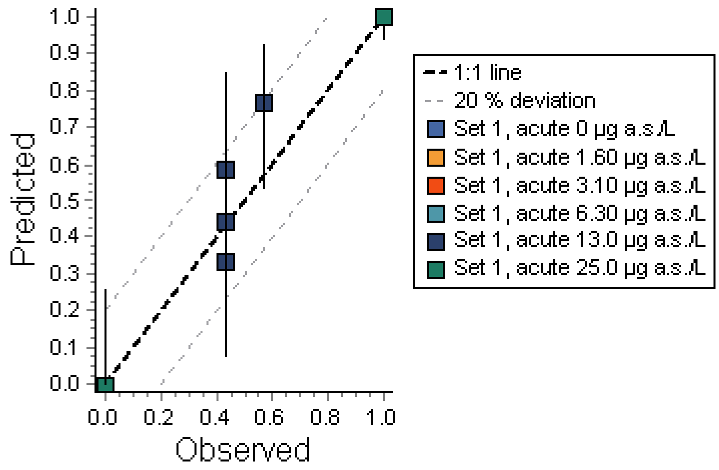

Observed vs. Predicted deaths plot for the calibration of GUTS-RED-SD:

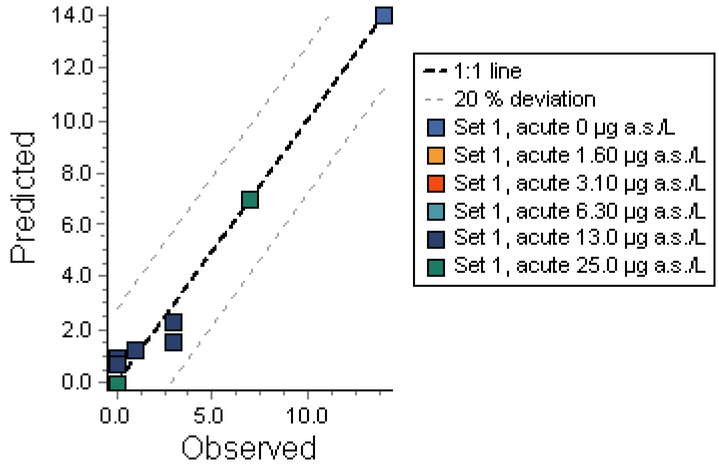

LCx versus time with confidence intervals (plotted for 16 days, GUTS-RED-SD):

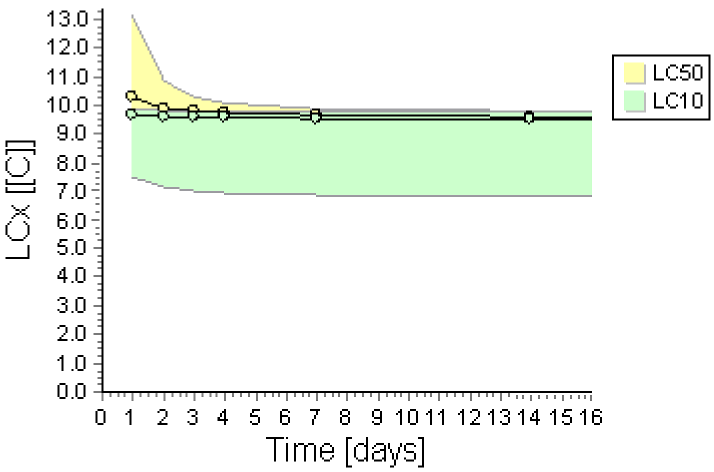
LCx [[C]]
13.0
12.0
11.0
10.0
9.0
8.0
7.0
6.0
5.0
4.0
3.0
2.0
1.0
0.0
0 1 2 3 4 5 6 7 8 9 10 11 12 13 14 15 16
Time [days]
LC50
LC10

Fitted parameters for GUTS-RED-IT:

Best fit parameter values and their 95% CI

kd: 4.882 (1.38 - 7.667)

mw: 9.763 (7.455 - 11.4)

hb: 10E-7 (1E-6* - 0.01316)

Fs: 1.05 (1.05* - 2.948)

* edge of 95% parameter CI has run into a boundary

(this may also affect CIs of other parameters)

Goodness of fit for calibration data (GUTS-RED-IT):

Model efficiency (NSE, r-square): 1

Normalised root-means-square error (NRMSE): 0.03639 %

Minus log-likelihood (MLL): 7.04

AIC: 22.07

Survival probability prediction error (SPPE) for each treatment:

Data set	Treatment	Value
1	acute 0 µg a.s./L	0.0004 %
1	acute 1.60 µg a.s./L	0.0004 %
1	acute 3.10 µg a.s./L	0.0004 %
1	acute 6.30 µg a.s./L	0.0004 %
1	acute 13.0 µg a.s./L	-0.04 %
1	acute 25.0 µg a.s./L	0 %

GUTS-RED-IT results table for LCx,t [[C]], with 95% CI:

Time [d]	LC50	LC20	LC10
1	9.837 (8.117 - 12.57)	9.657 (5.95 - 10.24)	9.554 (4.796 - 9.759)
2	9.763 (7.518 - 11.56)	9.585 (5.437 - 9.777)	9.482 (4.385 - 9.672)
3	9.763 (7.383 - 11.54)	9.584 (5.384 - 9.777)	9.481 (4.348 - 9.672)
4	9.763 (7.358 - 11.53)	9.584 (5.378 - 9.777)	9.481 (4.344 - 9.672)
7	9.763 (7.353 - 11.53)	9.584 (5.376 - 9.777)	9.481 (4.342 - 9.672)
14	9.763 (7.353 - 11.53)	9.584 (5.376 - 9.777)	9.481 (4.342 - 9.672)
21	9.763 (7.353 - 11.53)	9.584 (5.376 - 9.777)	9.481 (4.342 - 9.672)
28	9.763 (7.353 - 11.53)	9.584 (5.376 - 9.777)	9.481 (4.342 - 9.672)
42	9.763 (7.353 - 11.53)	9.584 (5.376 - 9.777)	9.481 (4.342 - 9.672)

50	9.763 (7.353 - 11.53)	9.584 (5.376 - 9.777)	9.481 (4.342 - 9.672)
100	9.763 (7.353 - 11.53)	9.584 (5.376 - 9.777)	9.481 (4.342 - 9.672)

<u>**Plots for GUTS-RED-IT calibration:**</u>

Parameter space plot for the calibration of GUTS-RED-IT:

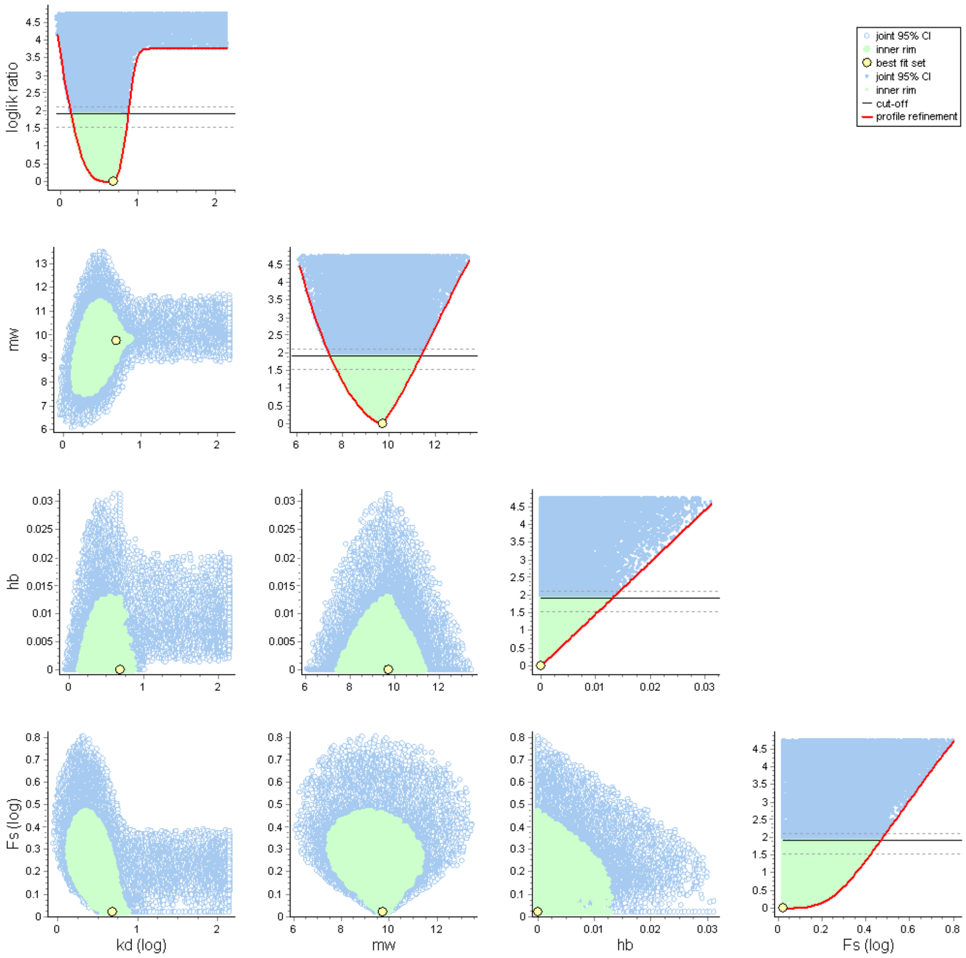

Exposure, damage and survival plots for the calibration of GUTS-RED-IT:

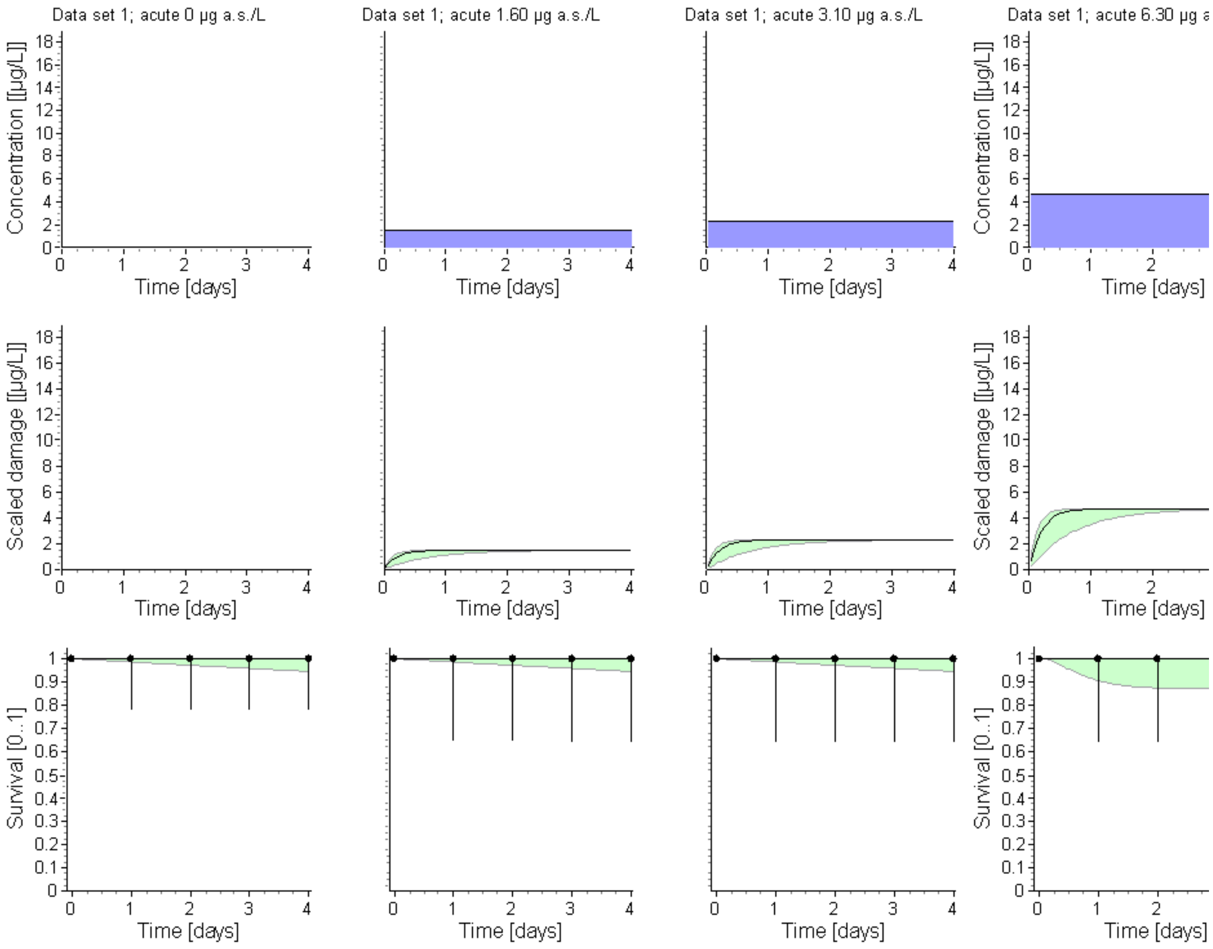

... continued plot:

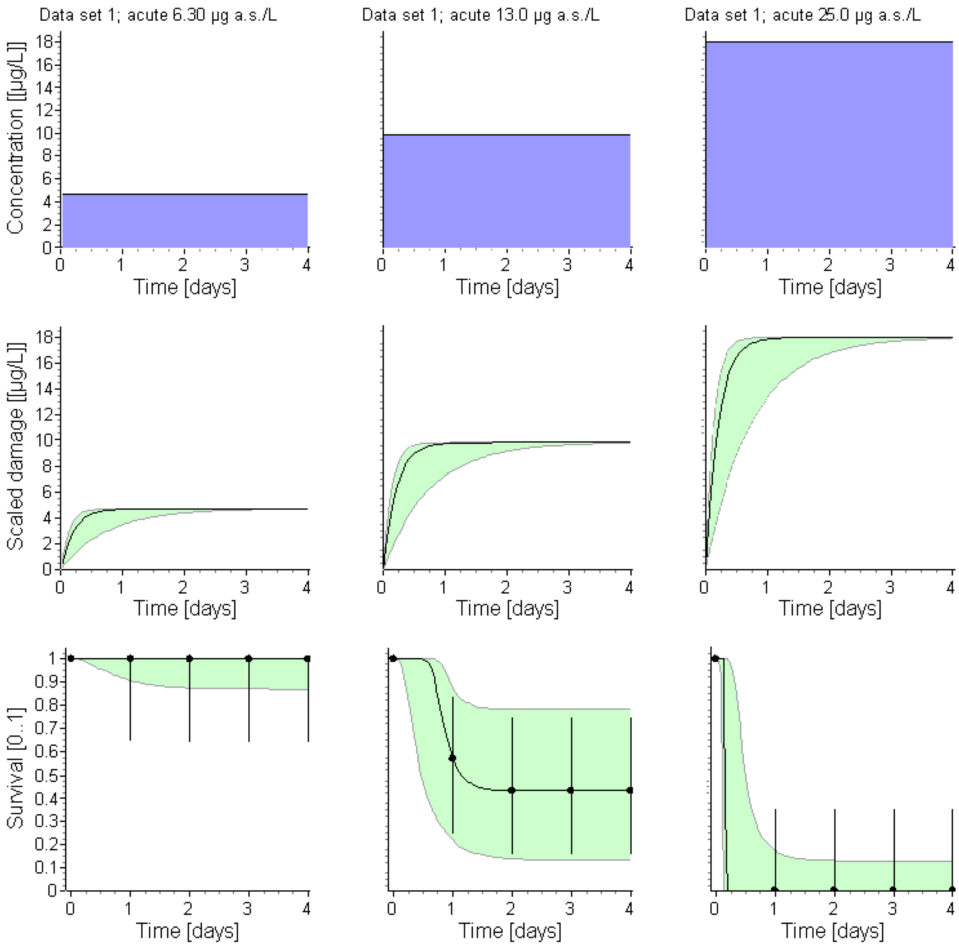

Observed vs. Predicted survival plot for the calibration of GUTS-RED-IT:

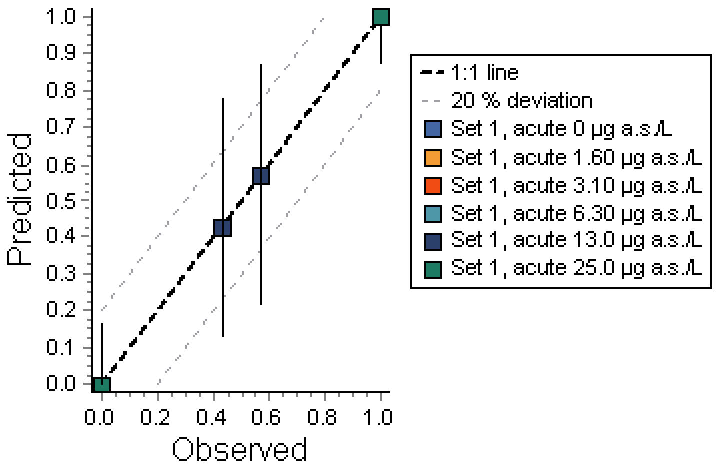

Observed vs. Predicted deaths plot for the calibration of GUTS-RED-IT:

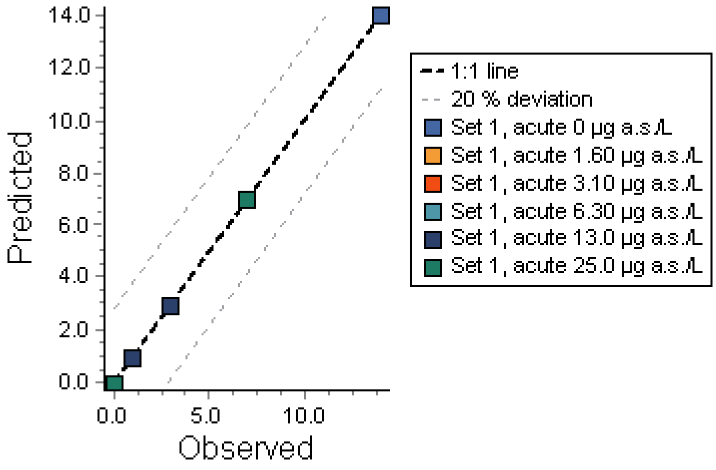

LCx versus time with confidence intervals (plotted for 16 days, GUTS-RED-IT):

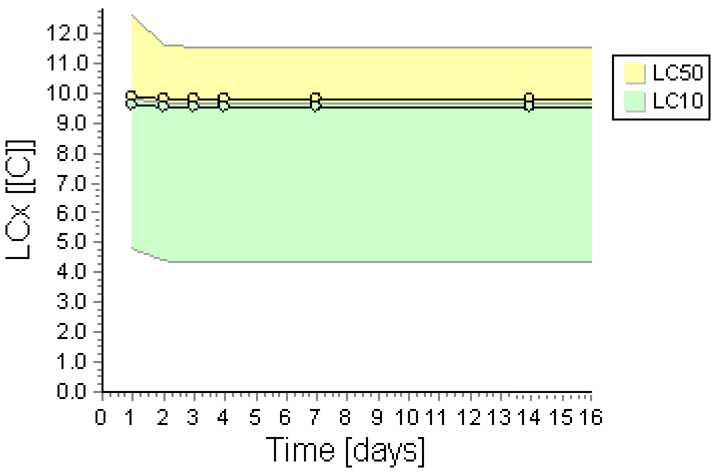

LC50
LC10
LCx [[C]]
Time [days]

Validation

No validation performed!

Predictions

No predictions performed!
