# Supplementary material for: Fish Species Sensitivity Ranking Depends on Pesticide Exposure Profiles
Source: Environ Toxicol Chem. 2022 Jun 6;41(7):1732–41. doi: 10.1002/etc.5348 (PMC9328144; doi:10.1002/etc.5348)
Supplement: Supplementary file 2 — Supporting information. [file ETC-41-1732-s004.zip › fits openGUTS standalone/calibration_jointFitHb_P_promelas.pdf]

openGUTS Report

Project:

calibration_jointFitHb

Project file:

No project file saved or loaded

Project description (optional):

No project description available

Software version:

openGUTS - 1.0

Date of report creation:

28/04/2020 12:51:18

Calibration

Calibration input data

Data set 1

File: openGUTSInput_1781

Description (optional):

Control group: 'acute 0 µg a.s./L'

Survival data of input data set 1:

Time [d]	acute 0 µg a.s./L	acute 0.630 µg a.s./L	acute 1.30 µg a.s./L	acute 2.50 µg a.s./L	acute 5.00 µg a.s./L	acute 10.0 µg a.s./L
0	14	7	7	7	7	7
1	14	7	7	7	7	3
2	14	7	7	7	7	2
3	14	7	7	7	7	0
4	14	7	7	7	4	0

Concentration data of input data set 1:

Time [d]	acute 0 µg a.s./L	acute 0.630 µg a.s./L	acute 1.30 µg a.s./L	acute 2.50 µg a.s./L	acute 5.00 µg a.s./L	acute 10.0 µg a.s./L
0	0	0.54	1.1	2.2	4.4	9.4

Data set 2

File: openGUTSInput_1781

Description (optional):

Control group: 'ELS 0 µg a.s./L'

Survival data of input data set 2:

Time [d]	ELS 0 µg a.s./L	ELS 0.250 µg a.s./L	ELS 0.500 µg a.s./L	ELS 1.00 µg a.s./L	ELS 2.00 µg a.s./L	ELS 4.00 µg a.s./L
0	212	109	105	107	106	104
1	212	109	105	107	106	104
2	212	109	105	107	106	102
3	212	108	105	106	106	99
4	210	108	104	104	104	98
5	209	108	103	104	104	98

6	176	95	90	88	87	65
7	176	95	90	88	87	57
8	175	95	90	88	87	56
9	175	95	90	87	87	55
10	174	94	87	87	87	54
11	174	94	85	86	87	54
12	174	94	85	86	87	54
13	174	93	83	86	87	53
14	174	93	83	85	86	48
15	174	93	83	85	86	46
16	174	92	83	85	86	45
17	171	92	83	85	86	45
18	171	92	83	85	86	44
19	171	92	83	85	86	44
20	170	92	83	85	86	44
21	170	92	83	84	86	43
22	168	92	83	83	85	43
23	167	92	83	82	85	43
24	167	92	83	81	85	43
25	165	92	80	81	85	42
26	165	92	80	81	85	42
27	165	92	80	81	85	42
28	164	90	78	80	85	42

Concentration data of input data set 2:

Time [d]	ELS 0 µg a.s./L	ELS 0.250 µg a.s./L	ELS 0.500 µg a.s./L	ELS 1.00 µg a.s./L	ELS 2.00 µg a.s./L	ELS 4.00 µg a.s./L
0	0	0.29	0.49	0.95	1.8	3.6

Calibration settings

Calibration parameter settings for GUTS-RED-SD:

Parameter	Fit	Min	Max	Scale
kd	Yes	0.001641	143.8	Log
mw	Yes	7.933E-5	9.306	Norm
hb	Yes	1E-6	0.07	Norm
bw	Yes	0.0004003	36288	Log
Fs	No	1	1	Norm

Calibration parameter settings for GUTS-RED-IT:

Parameter	Fit	Min	Max	Scale
kd	Yes	0.001641	143.8	Log
mw	Yes	7.933E-5	18.8	Norm
hb	Yes	1E-6	0.07	Norm
bw	No	Inf	Inf	Norm
Fs	Yes	1.05	20	Log

Calibration results

Fitted parameters for GUTS-RED-SD:

Best fit parameter values and their 95% CI

kd: 2.003 (1.021 - 5.049)

mw: 3.444 (3.247 - 3.537)

hb: 0.009225 (0.007788 - 0.01087)

bw: 0.2143 (0.0974 - 0.5414)

* edge of 95% parameter CI has run into a boundary

(this may also affect CIs of other parameters)

Goodness of fit for calibration data (GUTS-RED-SD):

Model efficiency (NSE, r-square): 0.8454

Normalised root-means-square error (NRMSE): 8.957 %

Minus log-likelihood (MLL): 1084.67

AIC: 2177.34

Survival probability prediction error (SPPE) for each treatment:

Data set	Treatment	Value
1	acute 0 µg a.s./L	3.623 %
1	acute 0.630 µg a.s./L	3.623 %
1	acute 1.30 µg a.s./L	3.623 %
1	acute 2.50 µg a.s./L	3.623 %
1	acute 5.00 µg a.s./L	2.166 %
1	acute 10.0 µg a.s./L	-1.48 %
2	ELS 0 µg a.s./L	0.1218 %
2	ELS 0.250 µg a.s./L	5.332 %
2	ELS 0.500 µg a.s./L	-2.95 %
2	ELS 1.00 µg a.s./L	-2.47 %
2	ELS 2.00 µg a.s./L	2.952 %
2	ELS 4.00 µg a.s./L	7.981 %

GUTS-RED-SD results table for LCx,t [[C]], with 95% CI:

Time [d]	LC50	LC20	LC10
1	11.24 (7.786 - 19.11)	6.982 (5.26 - 10.34)	5.754 (4.512 - 8.091)
2	6.314 (5.158 - 8.978)	4.651 (4.144 - 5.748)	4.168 (3.838 - 4.935)

3	5.107 (4.461 - 6.605)	4.098 (3.861 - 4.623)	3.81 (3.671 - 4.164)
4	4.595 (4.166 - 5.599)	3.876 (3.744 - 4.177)	3.675 (3.586 - 3.867)
7	4.028 (3.834 - 4.495)	3.649 (3.588 - 3.739)	3.547 (3.431 - 3.62)
14	3.709 (3.644 - 3.857)	3.533 (3.414 - 3.588)	3.487 (3.328 - 3.561)
21	3.614 (3.551 - 3.683)	3.5 (3.355 - 3.566)	3.471 (3.296 - 3.552)
28	3.569 (3.481 - 3.618)	3.485 (3.325 - 3.558)	3.463 (3.28 - 3.548)
42	3.525 (3.404 - 3.578)	3.47 (3.295 - 3.55)	3.456 (3.265 - 3.544)
50	3.512 (3.379 - 3.569)	3.466 (3.286 - 3.548)	3.454 (3.261 - 3.543)
100	3.477 (3.31 - 3.553)	3.454 (3.261 - 3.543)	3.449 (3.249 - 3.54)

Plots for GUTS-RED-SD calibration:

Parameter space plot for the calibration of GUTS-RED-SD:

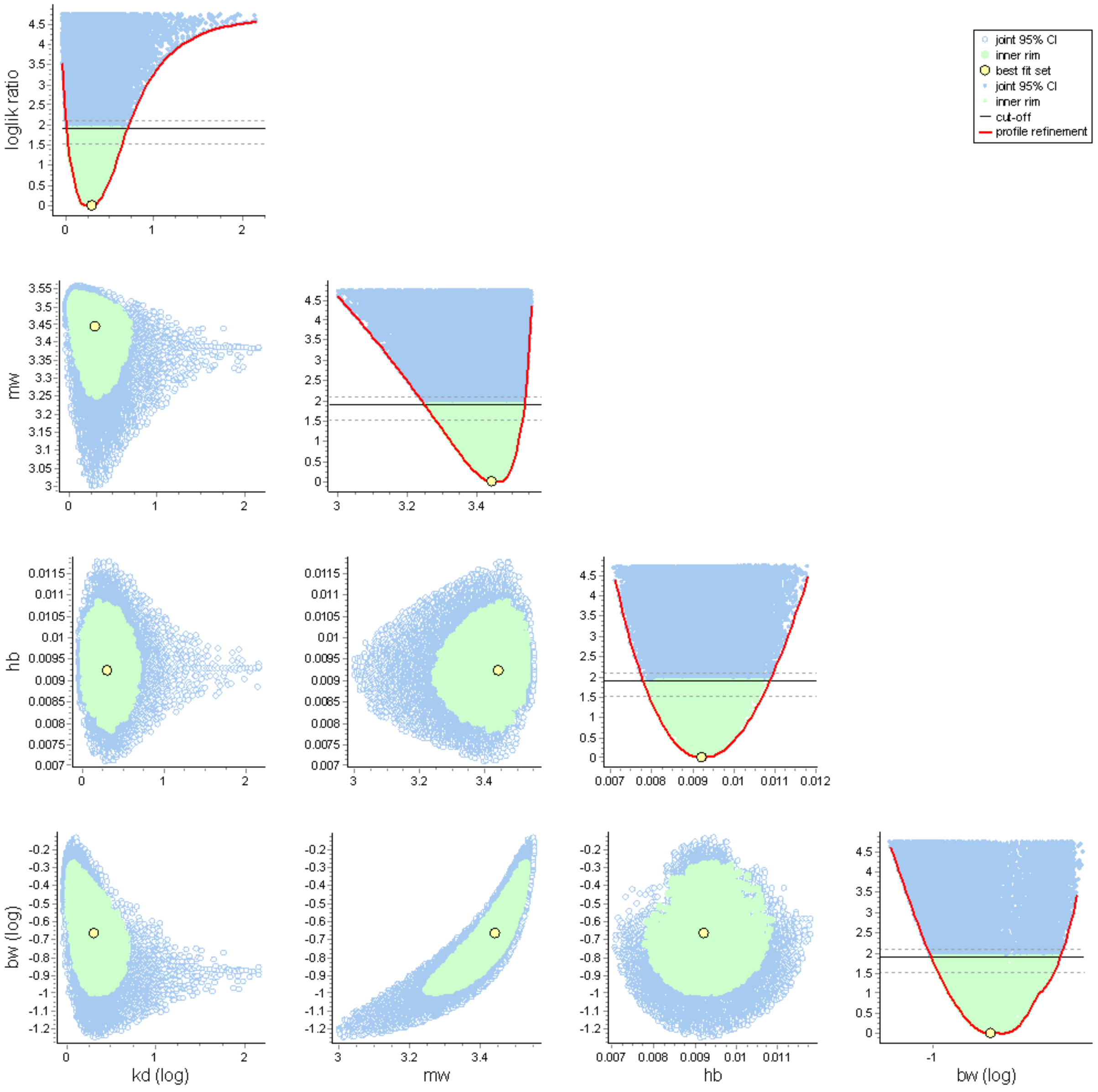

Exposure, damage and survival plots for the calibration of GUTS-RED-SD:

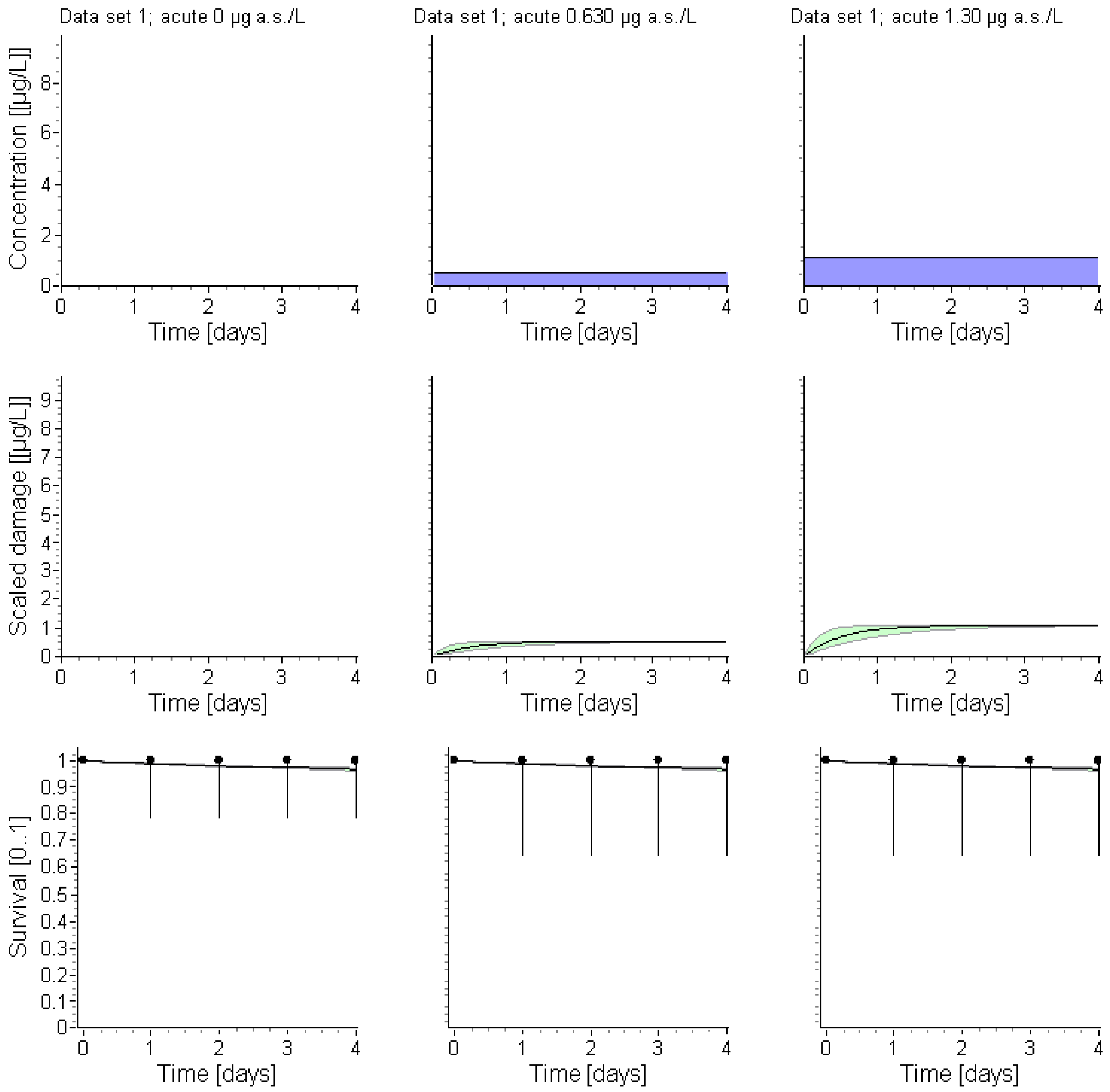

... continued plot:

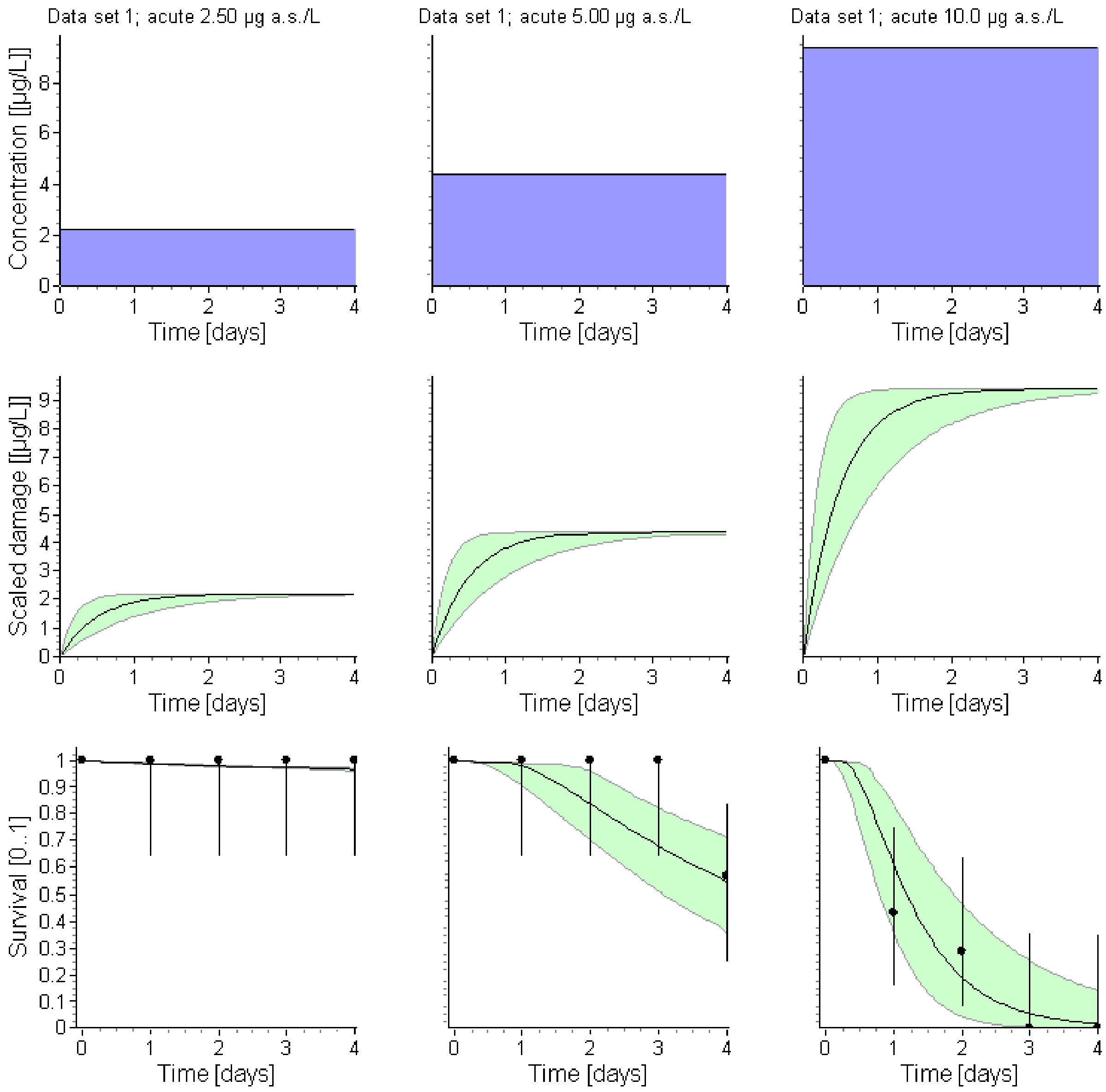

... continued plot:

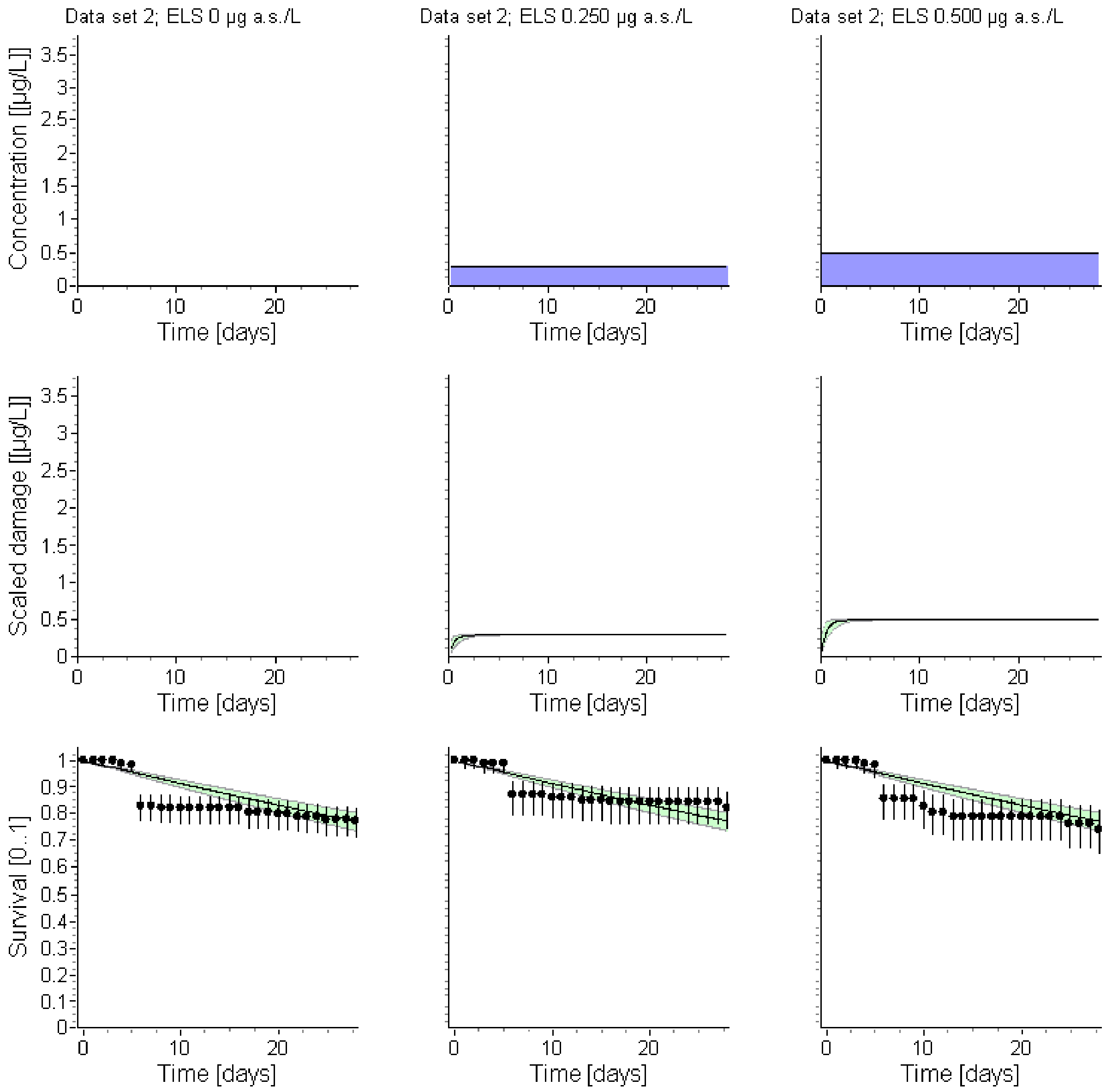

... continued plot:

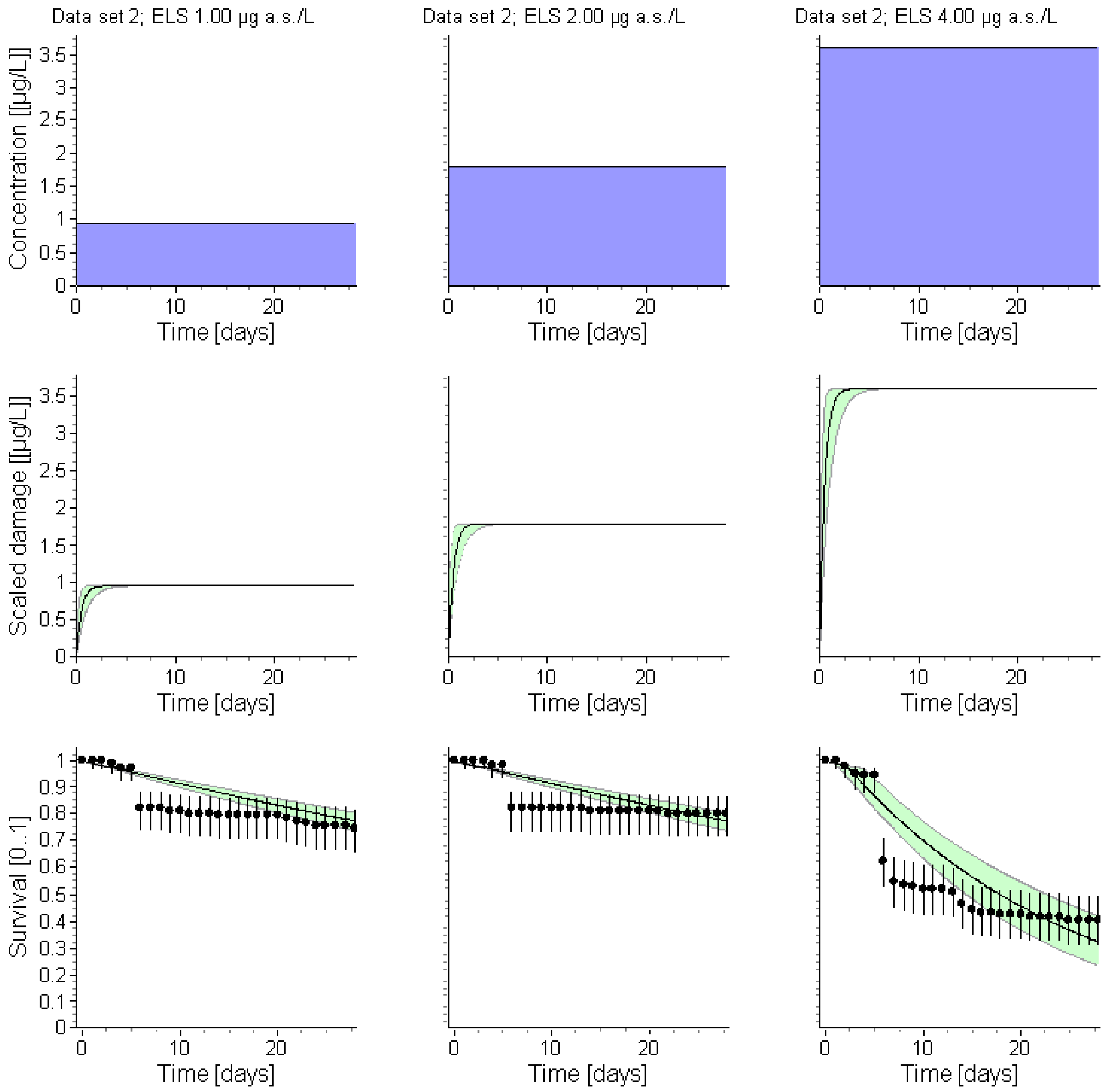

Observed vs. Predicted survival plot for the calibration of GUTS-RED-SD:

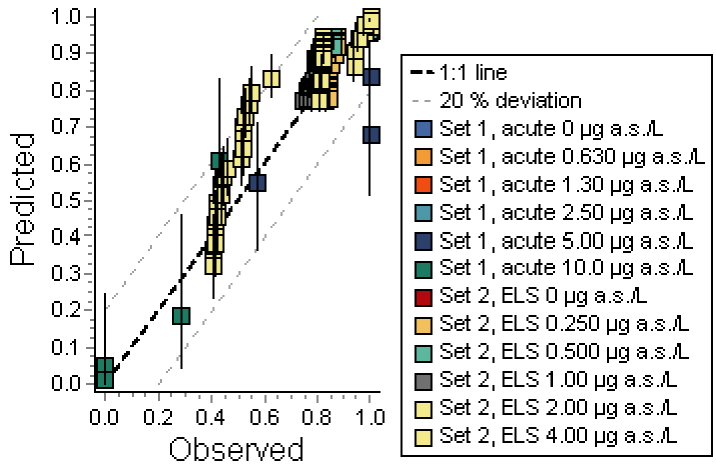

Observed vs. Predicted deaths plot for the calibration of GUTS-RED-SD:

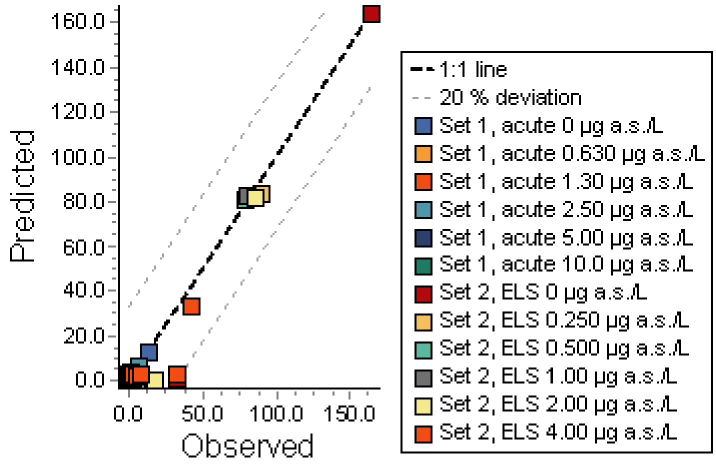

LCx versus time with confidence intervals (plotted for 16 days, GUTS-RED-SD):

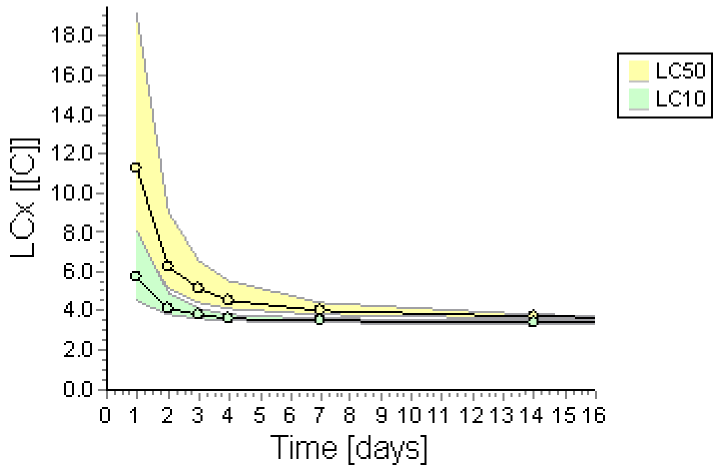

Fitted parameters for GUTS-RED-IT:

Best fit parameter values and their 95% CI

kd: 0.2774 (0.2089 - 0.4499)

mw: 3.464 (3.138 - 3.779)

hb: 0.008589 (0.007077 - 0.01041)

Fs: 2.108 (1.291 - 3.15)

* edge of 95% parameter CI has run into a boundary

(this may also affect CIs of other parameters)

Goodness of fit for calibration data (GUTS-RED-IT):

Model efficiency (NSE, r-square): 0.8676

Normalised root-means-square error (NRMSE): 8.207 %

Minus log-likelihood (MLL): 1058.16

AIC: 2124.31

Survival probability prediction error (SPPE) for each treatment:

Data set	Treatment	Value
1	acute 0 µg a.s./L	3.377 %
1	acute 0.630 µg a.s./L	3.379 %
1	acute 1.30 µg a.s./L	3.426 %
1	acute 2.50 µg a.s./L	4.81 %
1	acute 5.00 µg a.s./L	-9.33 %
1	acute 10.0 µg a.s./L	-4.85 %
2	ELS 0 µg a.s./L	-1.27 %
2	ELS 0.250 µg a.s./L	3.945 %
2	ELS 0.500 µg a.s./L	-4.33 %
2	ELS 1.00 µg a.s./L	-3.72 %
2	ELS 2.00 µg a.s./L	4.59 %
2	ELS 4.00 µg a.s./L	4.74 %

GUTS-RED-IT results table for LCx,t [[C]], with 95% CI:

Time [d]	LC50	LC20	LC10
1	14.3 (10.02 - 17.99)	10.78 (8.991 - 12.37)	9.144 (7.878 - 10.42)
2	8.136 (6.113 - 9.965)	6.136 (5.475 - 6.904)	5.202 (4.462 - 5.886)
3	6.132 (4.894 - 7.333)	4.625 (4.167 - 5.119)	3.921 (3.336 - 4.477)

4	5.168 (4.336 - 6.06)	3.898 (3.499 - 4.278)	3.305 (2.791 - 3.852)
7	4.044 (3.746 - 4.535)	3.05 (2.677 - 3.485)	2.586 (2.12 - 3.281)
14	3.537 (3.247 - 3.854)	2.667 (2.244 - 3.322)	2.262 (1.752 - 3.134)
21	3.474 (3.154 - 3.797)	2.62 (2.162 - 3.315)	2.222 (1.686 - 3.128)
28	3.465 (3.134 - 3.791)	2.614 (2.145 - 3.315)	2.216 (1.673 - 3.127)
42	3.464 (3.129 - 3.79)	2.612 (2.141 - 3.315)	2.215 (1.669 - 3.127)
50	3.464 (3.129 - 3.79)	2.612 (2.141 - 3.315)	2.215 (1.669 - 3.127)
100	3.464 (3.129 - 3.79)	2.612 (2.141 - 3.315)	2.215 (1.669 - 3.127)

Plots for GUTS-RED-IT calibration:

Parameter space plot for the calibration of GUTS-RED-IT:

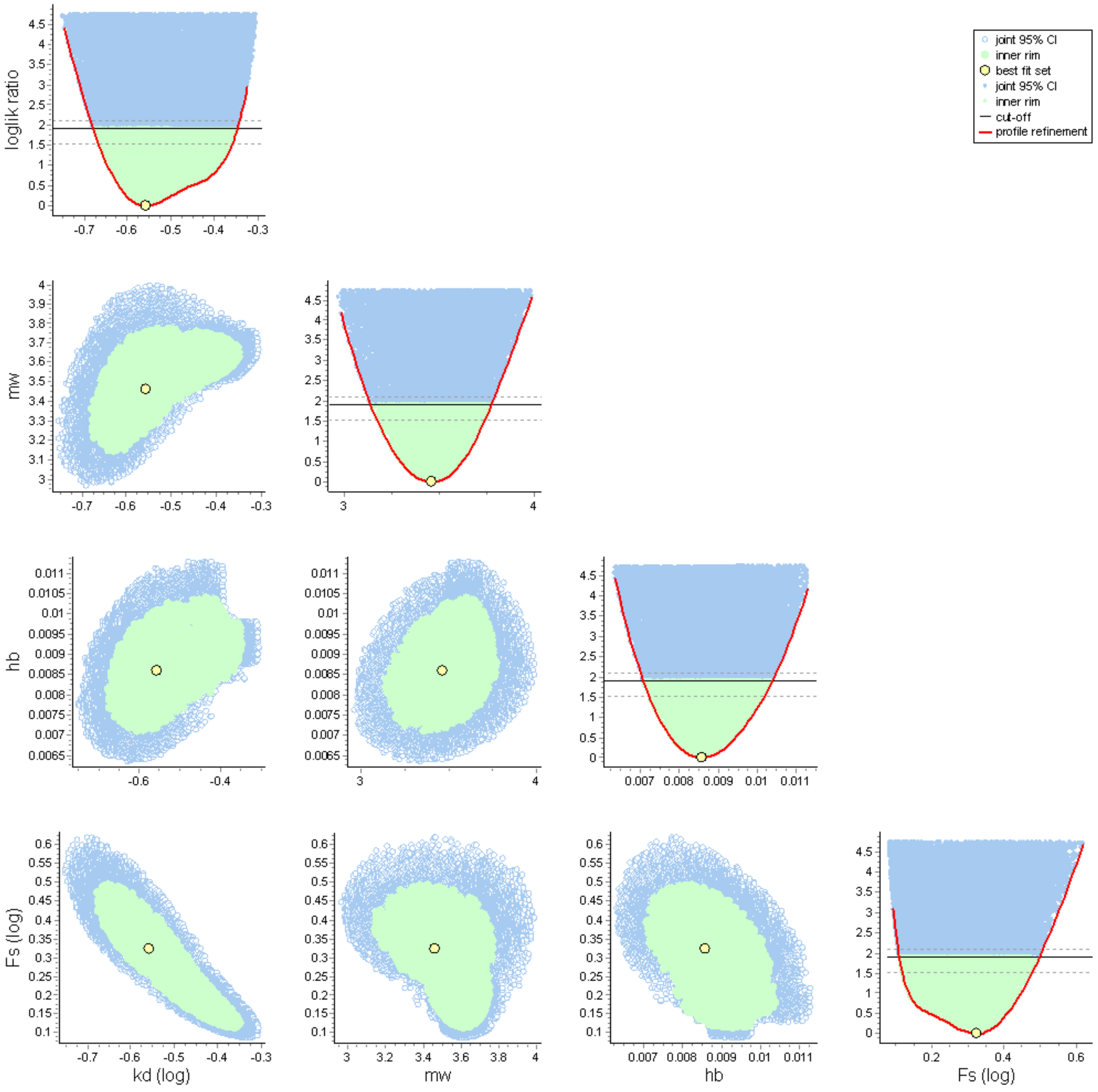

Exposure, damage and survival plots for the calibration of GUTS-RED-IT:

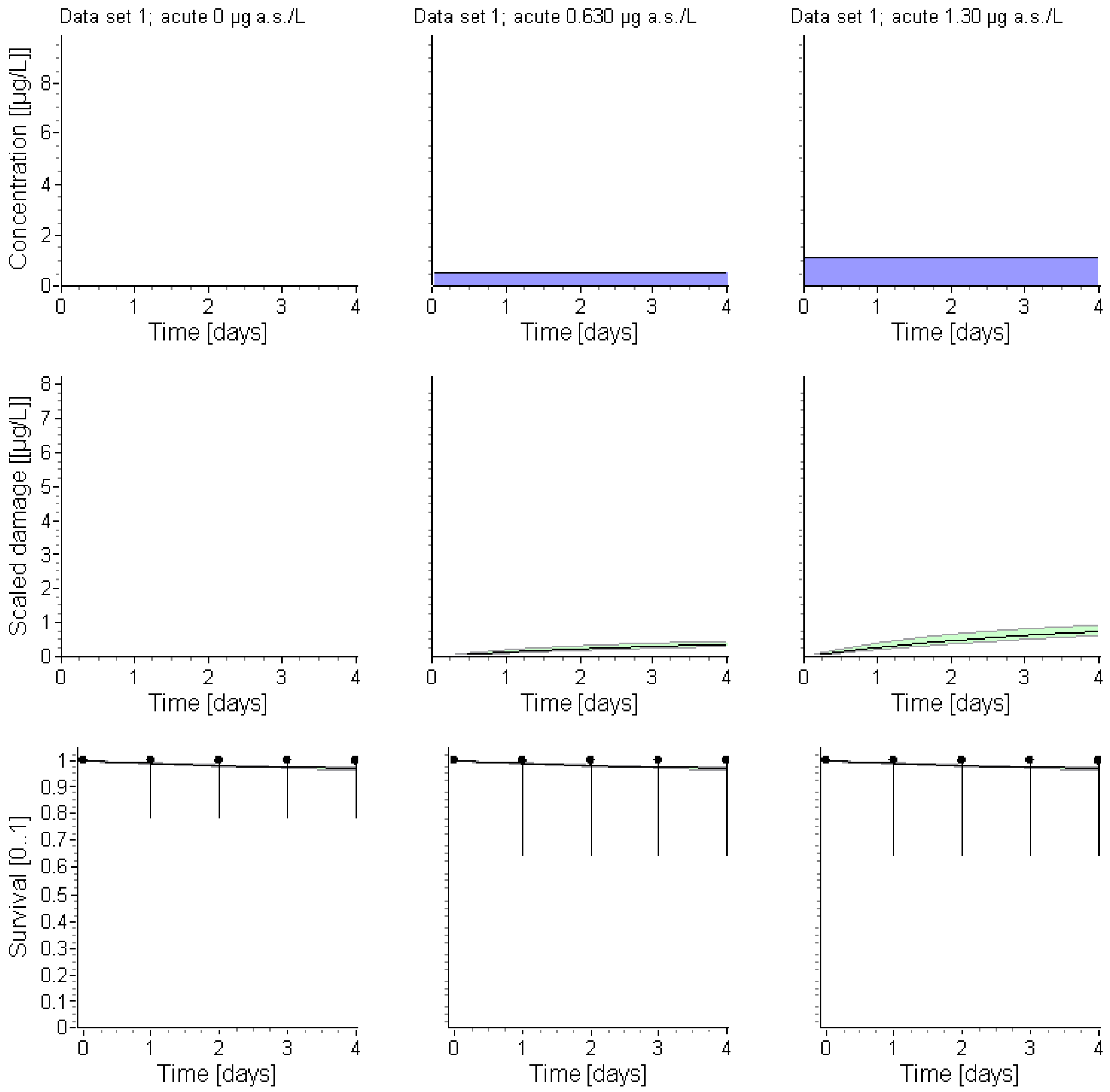

... continued plot:

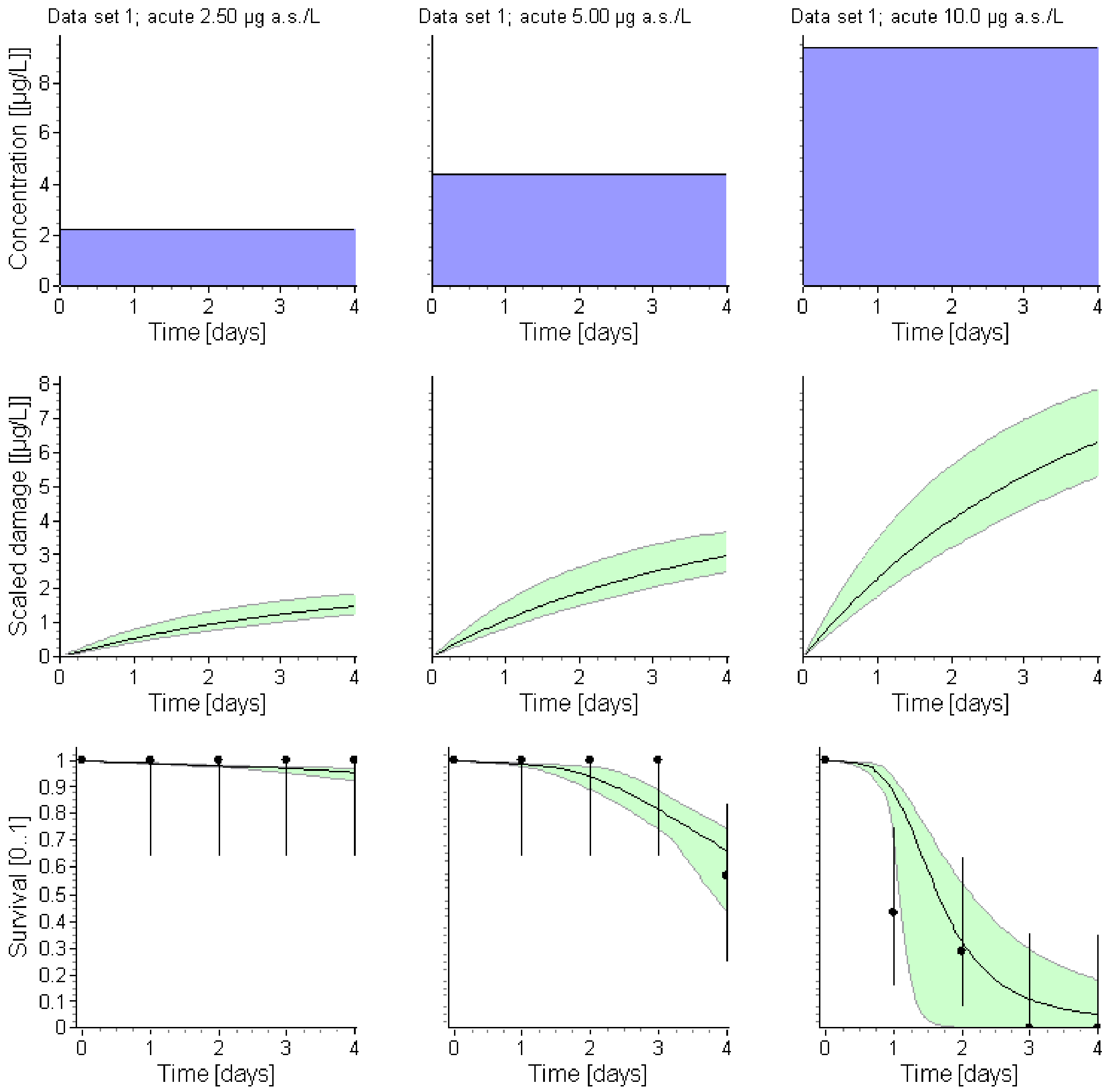

... continued plot:

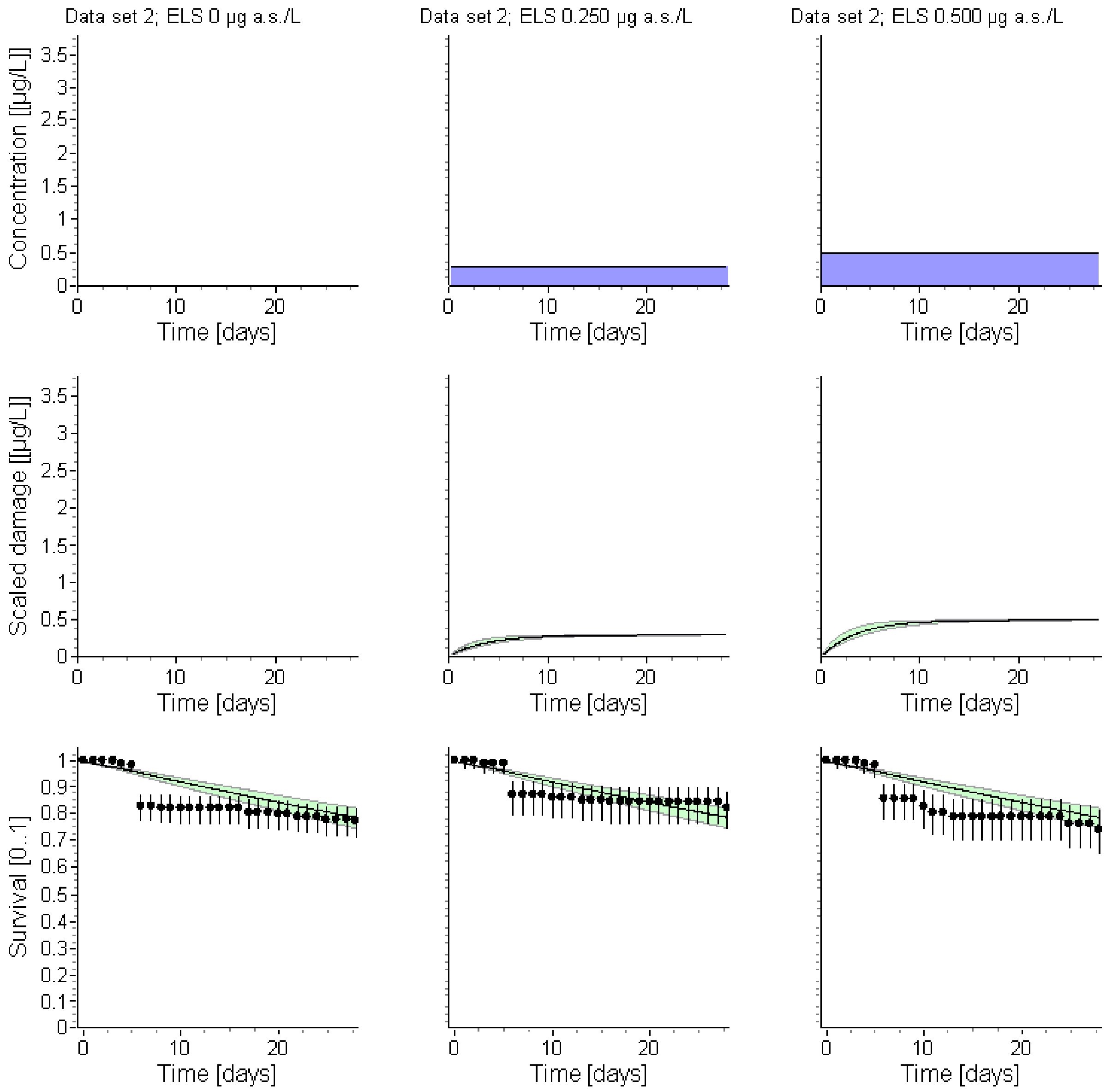

... continued plot:

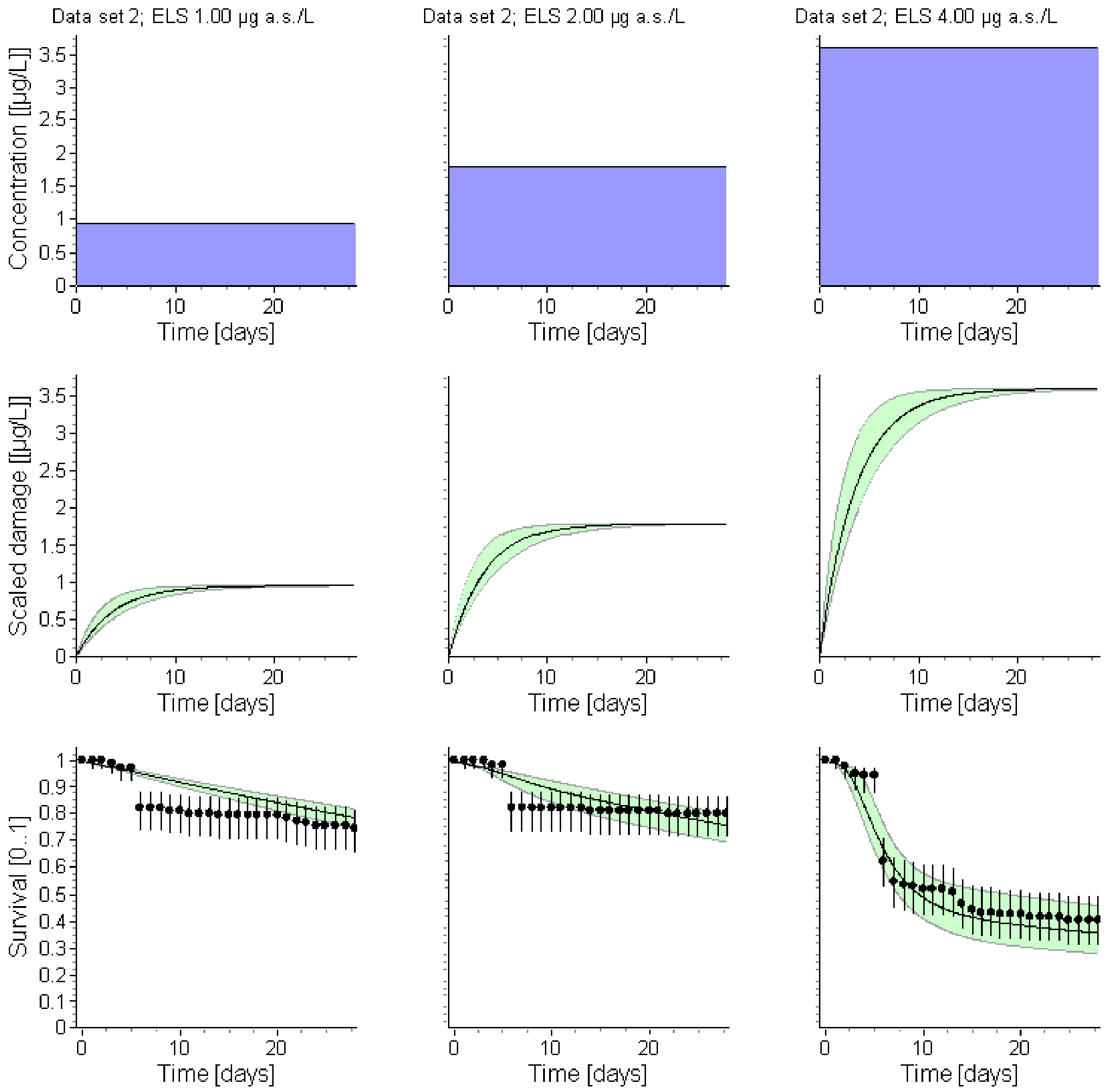

Observed vs. Predicted survival plot for the calibration of GUTS-RED-IT:

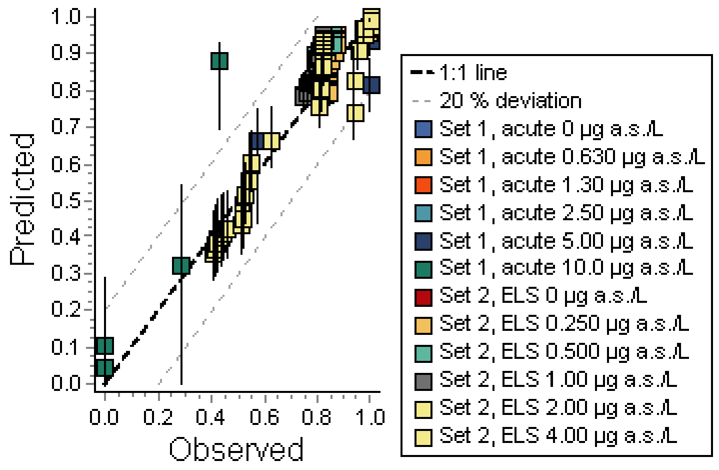

Observed vs. Predicted deaths plot for the calibration of GUTS-RED-IT:

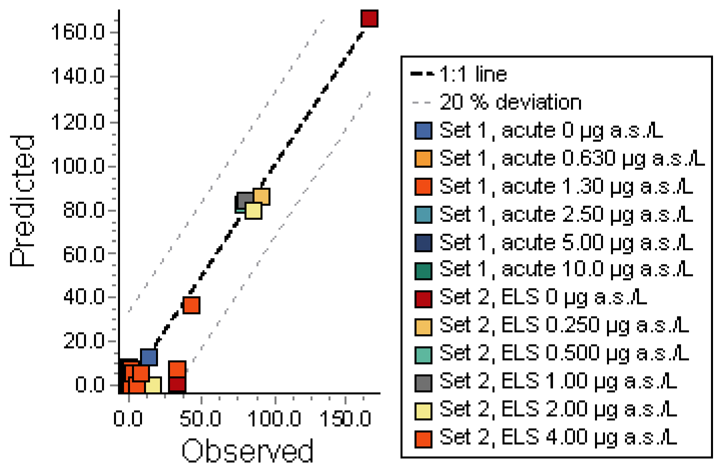

LCx versus time with confidence intervals (plotted for 16 days, GUTS-RED-IT):

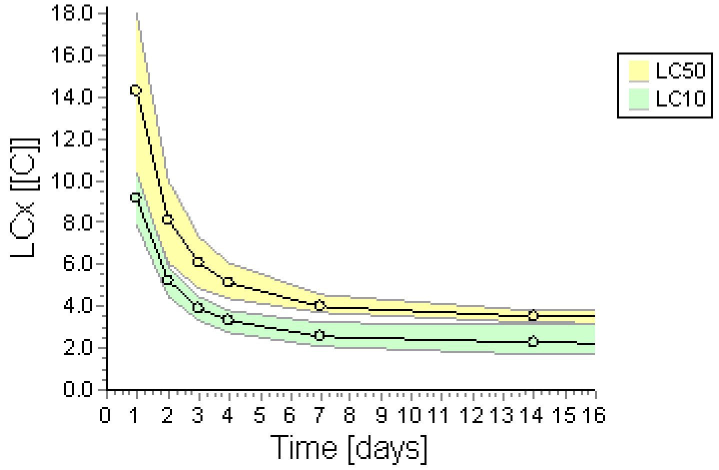

Validation

No validation performed!

Predictions

No predictions performed!
