# Supplementary figures and images for: Fish Species Sensitivity Ranking Depends on Pesticide Exposure Profiles
Source: Environ Toxicol Chem. 2022 Jun 6;41(7):1732–41. doi: 10.1002/etc.5348 (PMC9328144; doi:10.1002/etc.5348)

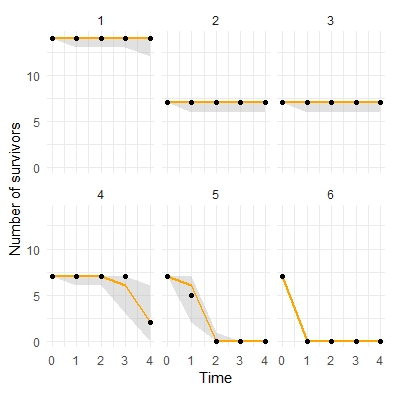

Supplement: Supplementary file 3 — Supporting information. [file ETC-41-1732-s002.zip › morse/Cyprinodon variegatus/IT_calibration_Nsurv.jpg]

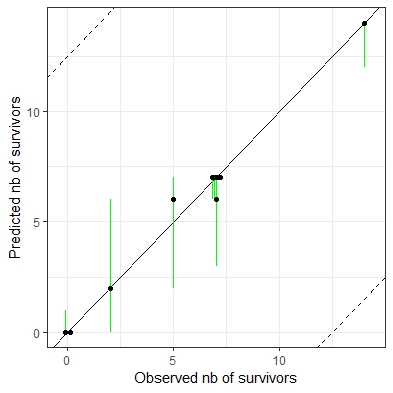

Supplement: Supplementary file 3 — Supporting information. [file ETC-41-1732-s002.zip › morse/Cyprinodon variegatus/IT_calibration_ppc.jpg]

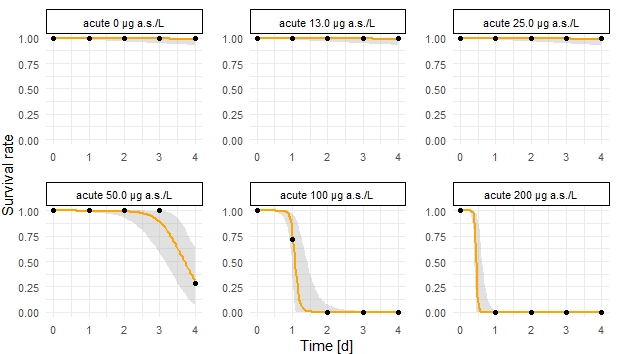

Supplement: Supplementary file 3 — Supporting information. [file ETC-41-1732-s002.zip › morse/Cyprinodon variegatus/IT_calibration_report.jpg]

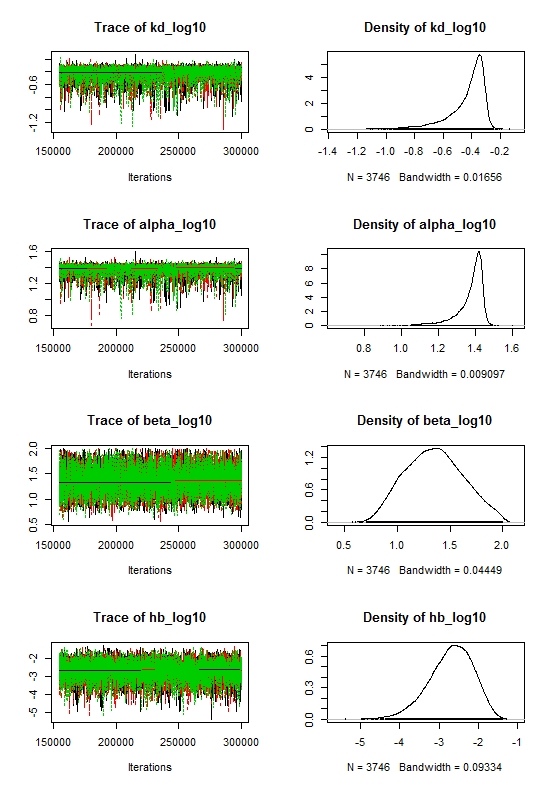

Supplement: Supplementary file 3 — Supporting information. [file ETC-41-1732-s002.zip › morse/Cyprinodon variegatus/IT_MCMC_Chains.jpg]

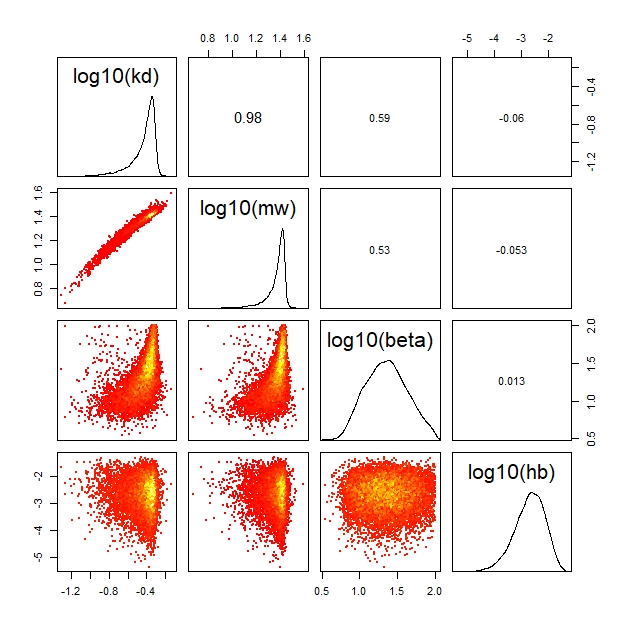

Supplement: Supplementary file 3 — Supporting information. [file ETC-41-1732-s002.zip › morse/Cyprinodon variegatus/IT_MCMC_pairs_log10_report.jpg]

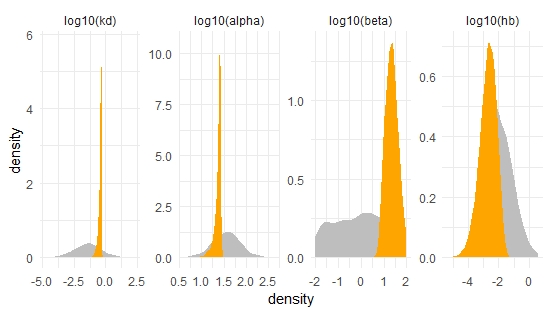

Supplement: Supplementary file 3 — Supporting information. [file ETC-41-1732-s002.zip › morse/Cyprinodon variegatus/IT_post_prior_distributions.jpg]

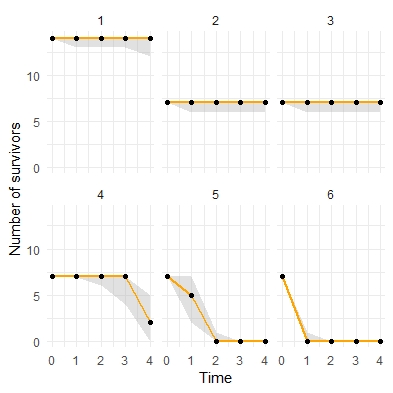

Supplement: Supplementary file 3 — Supporting information. [file ETC-41-1732-s002.zip › morse/Cyprinodon variegatus/SD_calibration_Nsurv.jpg]

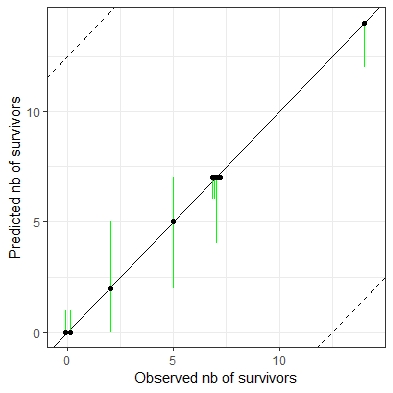

Supplement: Supplementary file 3 — Supporting information. [file ETC-41-1732-s002.zip › morse/Cyprinodon variegatus/SD_calibration_ppc.jpg]

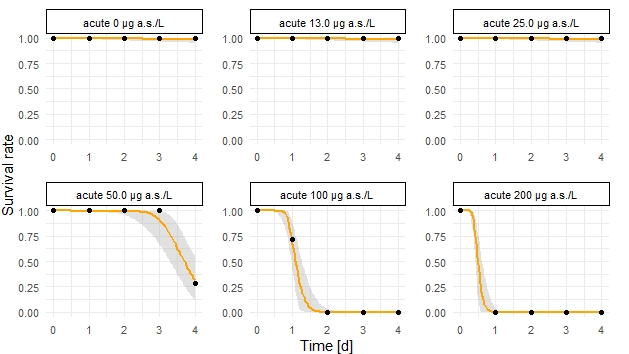

Supplement: Supplementary file 3 — Supporting information. [file ETC-41-1732-s002.zip › morse/Cyprinodon variegatus/SD_calibration_report.jpg]

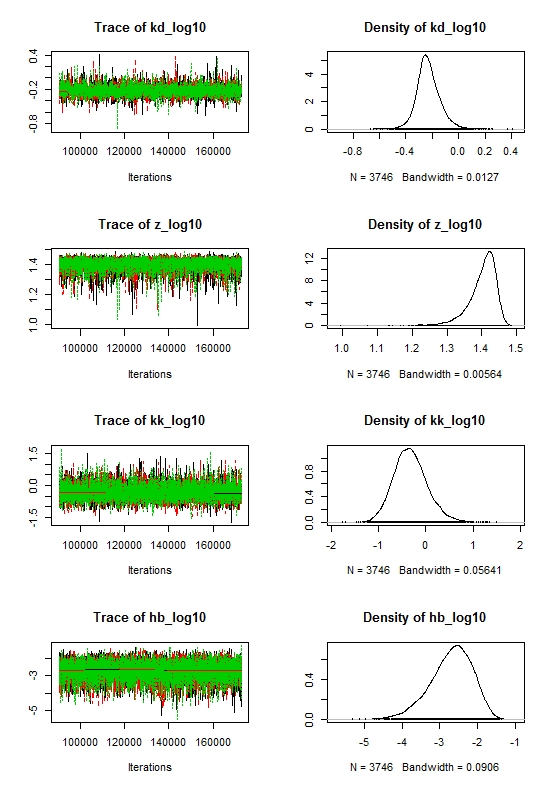

Supplement: Supplementary file 3 — Supporting information. [file ETC-41-1732-s002.zip › morse/Cyprinodon variegatus/SD_MCMC_Chains.jpg]

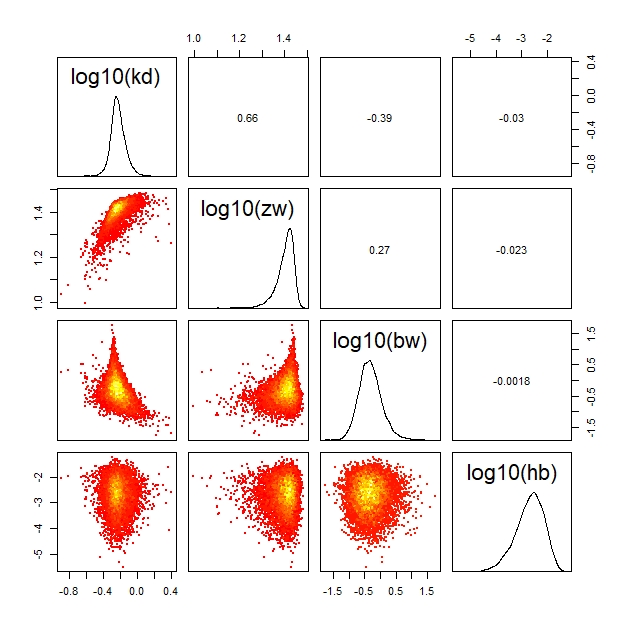

Supplement: Supplementary file 3 — Supporting information. [file ETC-41-1732-s002.zip › morse/Cyprinodon variegatus/SD_MCMC_pairs_log10_report.jpg]

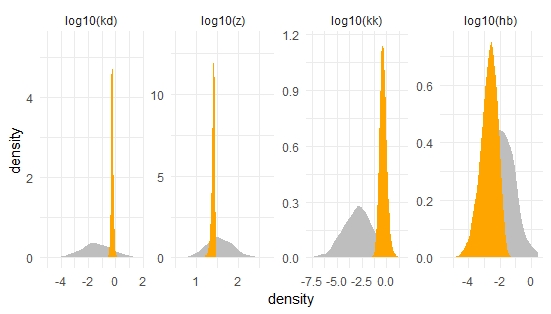

Supplement: Supplementary file 3 — Supporting information. [file ETC-41-1732-s002.zip › morse/Cyprinodon variegatus/SD_post_prior_distributions.jpg]

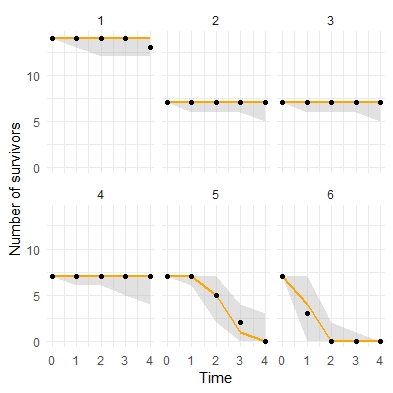

Supplement: Supplementary file 3 — Supporting information. [file ETC-41-1732-s002.zip › morse/Cyprinus carpio/IT_calibration_Nsurv.jpg]

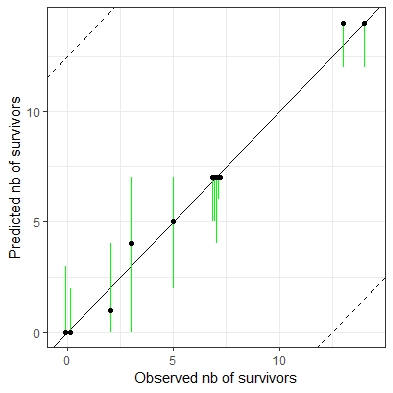

Supplement: Supplementary file 3 — Supporting information. [file ETC-41-1732-s002.zip › morse/Cyprinus carpio/IT_calibration_ppc.jpg]

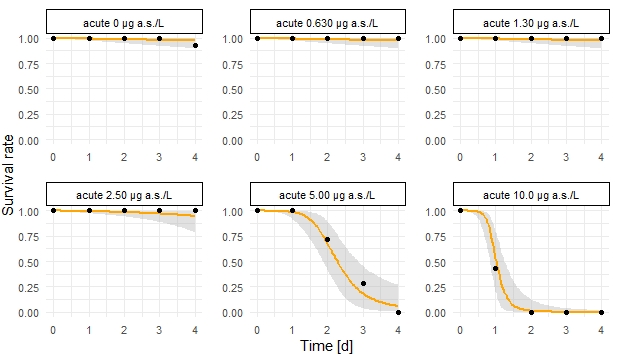

Supplement: Supplementary file 3 — Supporting information. [file ETC-41-1732-s002.zip › morse/Cyprinus carpio/IT_calibration_report.jpg]

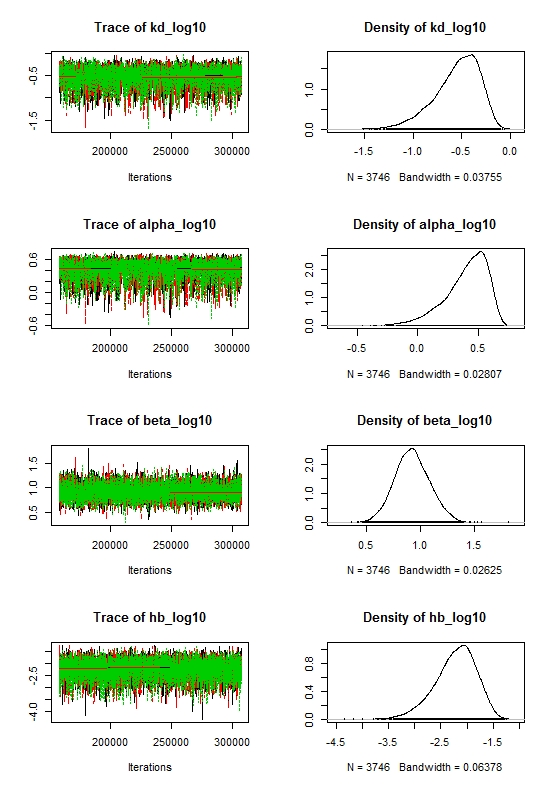

Supplement: Supplementary file 3 — Supporting information. [file ETC-41-1732-s002.zip › morse/Cyprinus carpio/IT_MCMC_Chains.jpg]

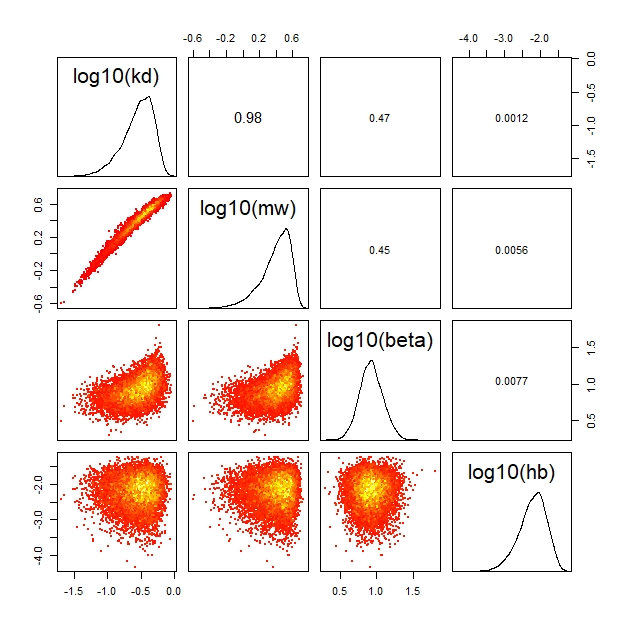

Supplement: Supplementary file 3 — Supporting information. [file ETC-41-1732-s002.zip › morse/Cyprinus carpio/IT_MCMC_pairs_log10_report.jpg]

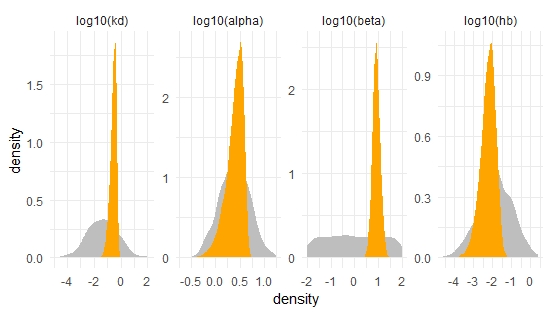

Supplement: Supplementary file 3 — Supporting information. [file ETC-41-1732-s002.zip › morse/Cyprinus carpio/IT_post_prior_distributions.jpg]

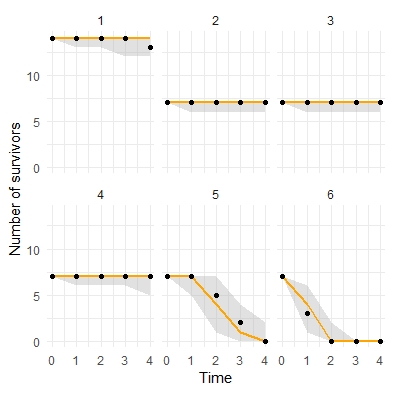

Supplement: Supplementary file 3 — Supporting information. [file ETC-41-1732-s002.zip › morse/Cyprinus carpio/SD_calibration_Nsurv.jpg]

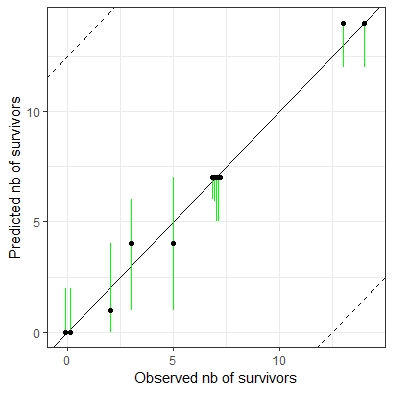

Supplement: Supplementary file 3 — Supporting information. [file ETC-41-1732-s002.zip › morse/Cyprinus carpio/SD_calibration_ppc.jpg]

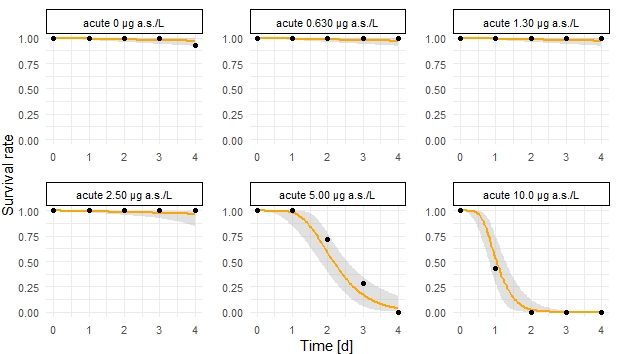

Supplement: Supplementary file 3 — Supporting information. [file ETC-41-1732-s002.zip › morse/Cyprinus carpio/SD_calibration_report.jpg]

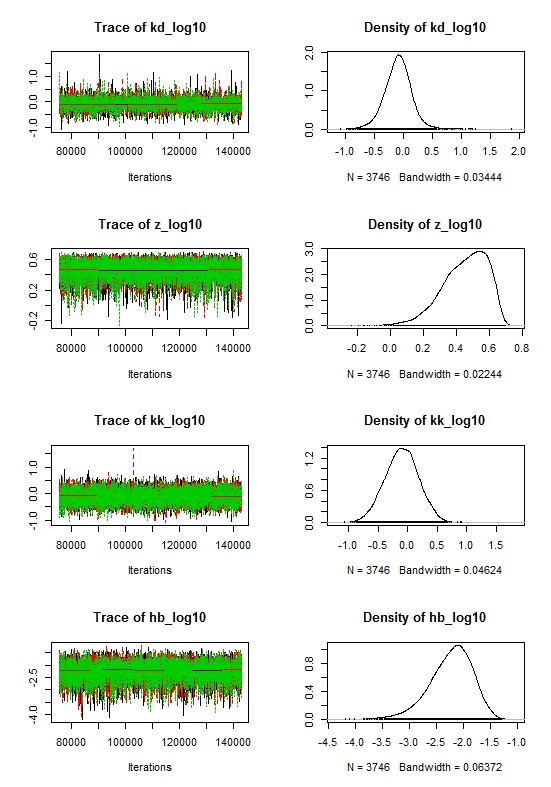

Supplement: Supplementary file 3 — Supporting information. [file ETC-41-1732-s002.zip › morse/Cyprinus carpio/SD_MCMC_Chains.jpg]

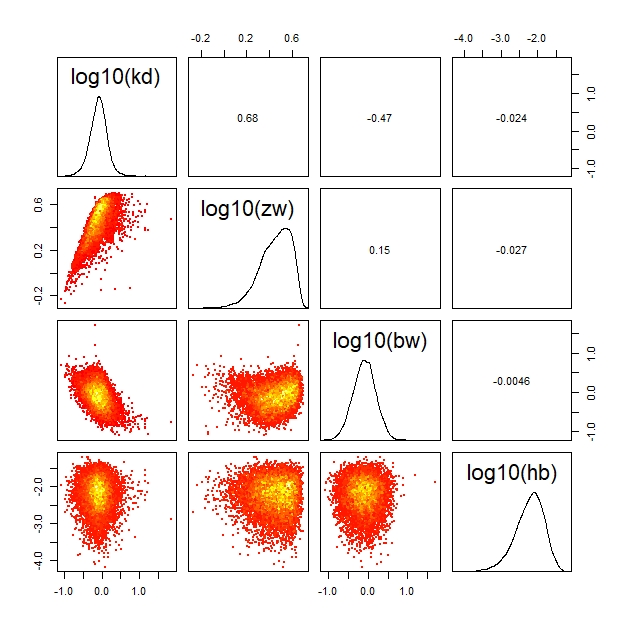

Supplement: Supplementary file 3 — Supporting information. [file ETC-41-1732-s002.zip › morse/Cyprinus carpio/SD_MCMC_pairs_log10_report.jpg]

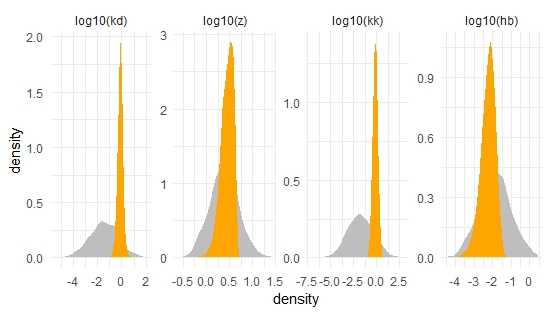

Supplement: Supplementary file 3 — Supporting information. [file ETC-41-1732-s002.zip › morse/Cyprinus carpio/SD_post_prior_distributions.jpg]

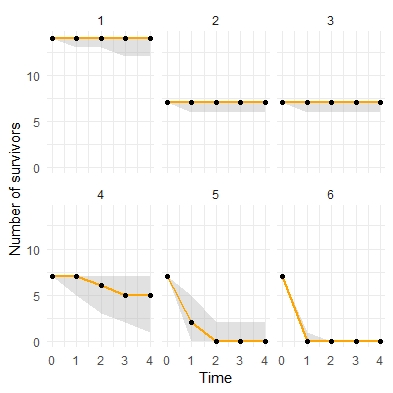

Supplement: Supplementary file 3 — Supporting information. [file ETC-41-1732-s002.zip › morse/Lepomis macrochirus/IT_calibration_Nsurv.jpg]

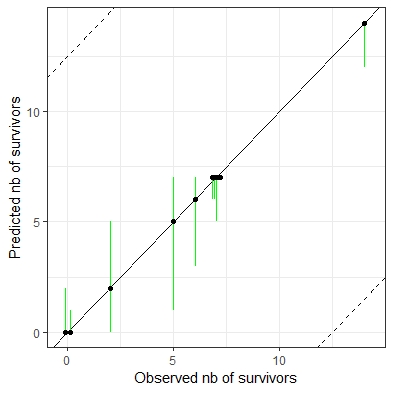

Supplement: Supplementary file 3 — Supporting information. [file ETC-41-1732-s002.zip › morse/Lepomis macrochirus/IT_calibration_ppc.jpg]

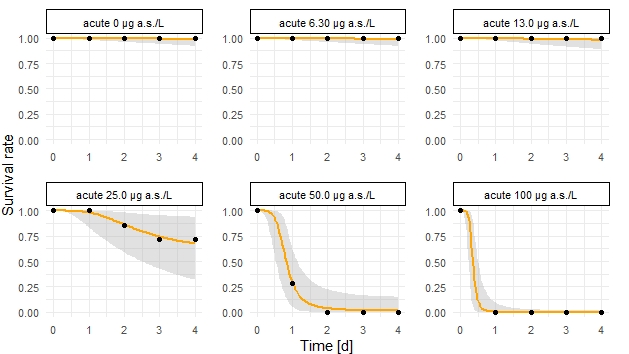

Supplement: Supplementary file 3 — Supporting information. [file ETC-41-1732-s002.zip › morse/Lepomis macrochirus/IT_calibration_report.jpg]

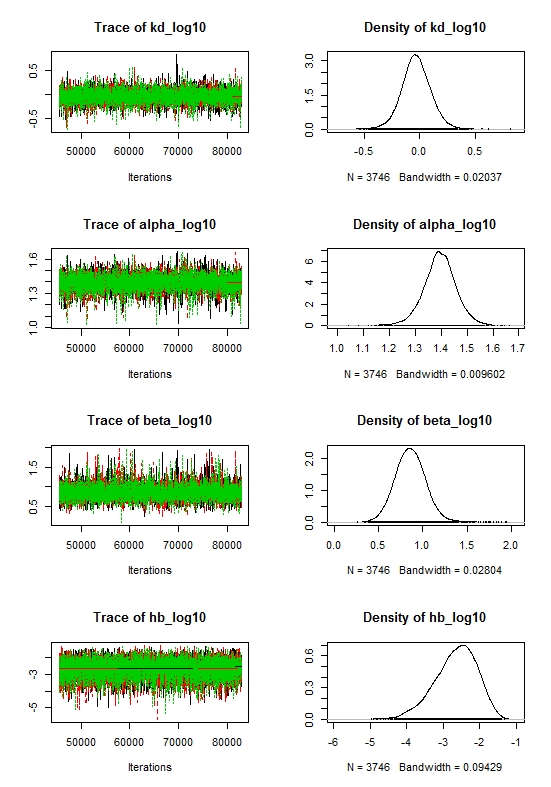

Supplement: Supplementary file 3 — Supporting information. [file ETC-41-1732-s002.zip › morse/Lepomis macrochirus/IT_MCMC_Chains.jpg]

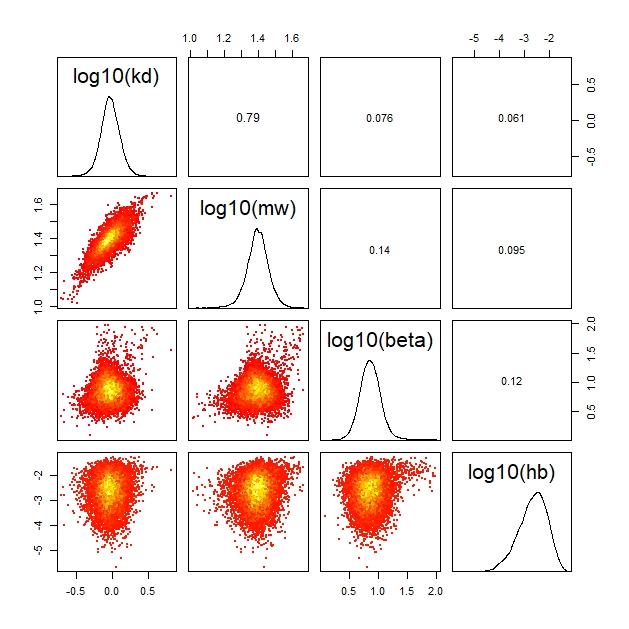

Supplement: Supplementary file 3 — Supporting information. [file ETC-41-1732-s002.zip › morse/Lepomis macrochirus/IT_MCMC_pairs_log10_report.jpg]

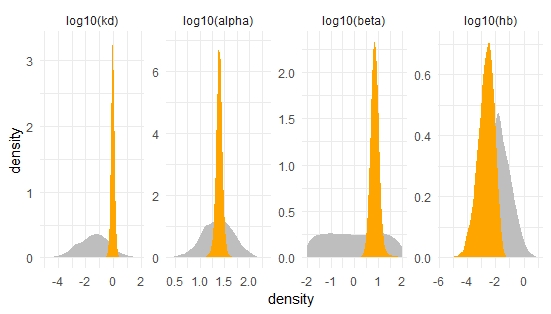

Supplement: Supplementary file 3 — Supporting information. [file ETC-41-1732-s002.zip › morse/Lepomis macrochirus/IT_post_prior_distributions.jpg]

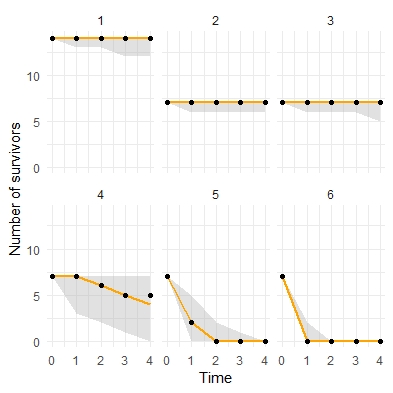

Supplement: Supplementary file 3 — Supporting information. [file ETC-41-1732-s002.zip › morse/Lepomis macrochirus/SD_calibration_Nsurv.jpg]

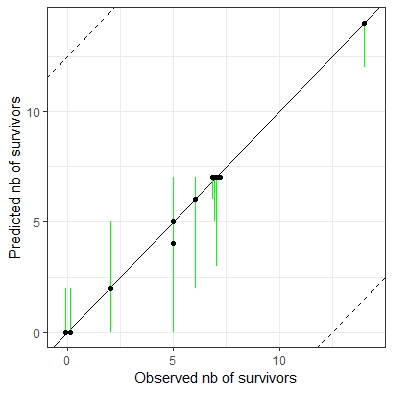

Supplement: Supplementary file 3 — Supporting information. [file ETC-41-1732-s002.zip › morse/Lepomis macrochirus/SD_calibration_ppc.jpg]

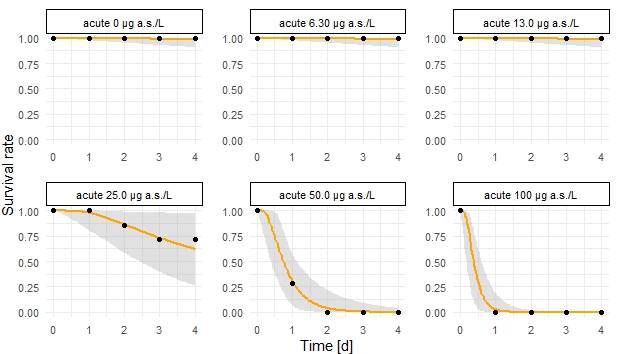

Supplement: Supplementary file 3 — Supporting information. [file ETC-41-1732-s002.zip › morse/Lepomis macrochirus/SD_calibration_report.jpg]

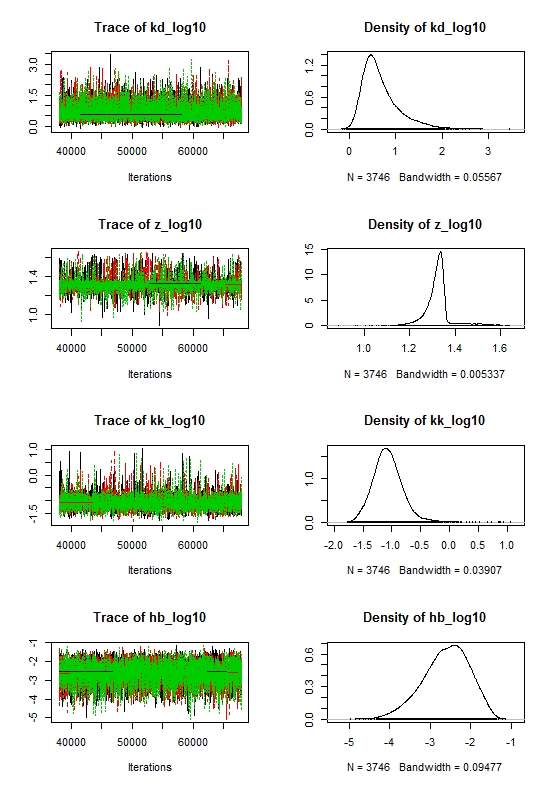

Supplement: Supplementary file 3 — Supporting information. [file ETC-41-1732-s002.zip › morse/Lepomis macrochirus/SD_MCMC_Chains.jpg]

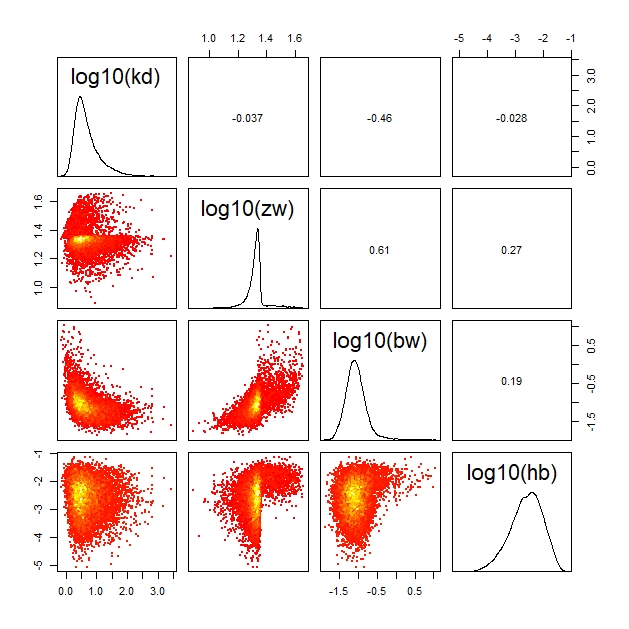

Supplement: Supplementary file 3 — Supporting information. [file ETC-41-1732-s002.zip › morse/Lepomis macrochirus/SD_MCMC_pairs_log10_report.jpg]

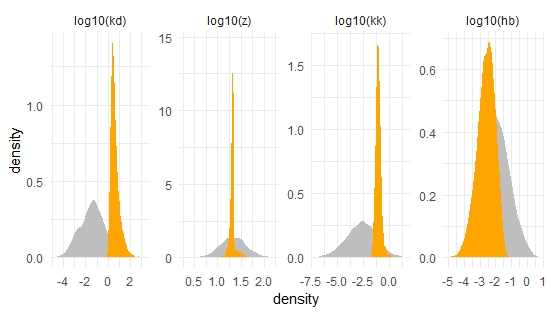

Supplement: Supplementary file 3 — Supporting information. [file ETC-41-1732-s002.zip › morse/Lepomis macrochirus/SD_post_prior_distributions.jpg]

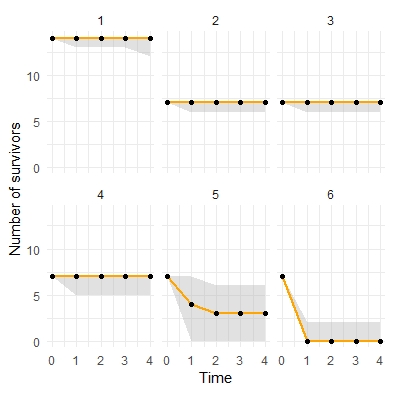

Supplement: Supplementary file 3 — Supporting information. [file ETC-41-1732-s002.zip › morse/Oncorhynchus mykiss/IT_calibration_Nsurv.jpg]

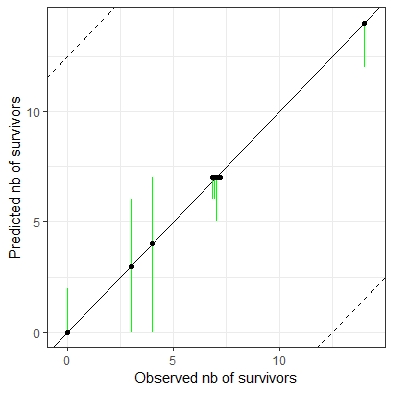

Supplement: Supplementary file 3 — Supporting information. [file ETC-41-1732-s002.zip › morse/Oncorhynchus mykiss/IT_calibration_ppc.jpg]

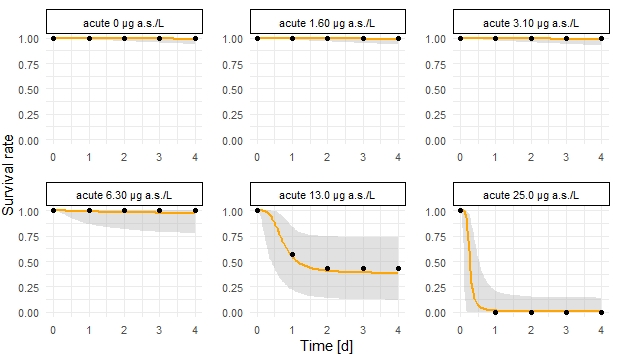

Supplement: Supplementary file 3 — Supporting information. [file ETC-41-1732-s002.zip › morse/Oncorhynchus mykiss/IT_calibration_report.jpg]

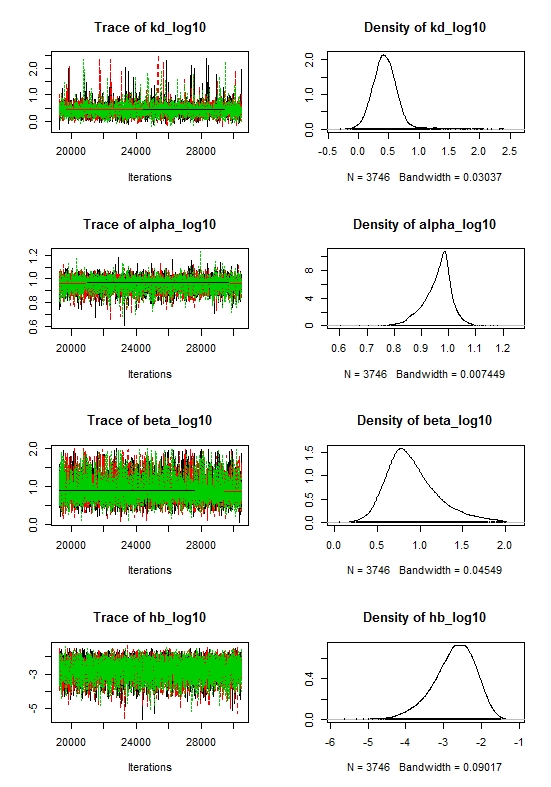

Supplement: Supplementary file 3 — Supporting information. [file ETC-41-1732-s002.zip › morse/Oncorhynchus mykiss/IT_MCMC_Chains.jpg]

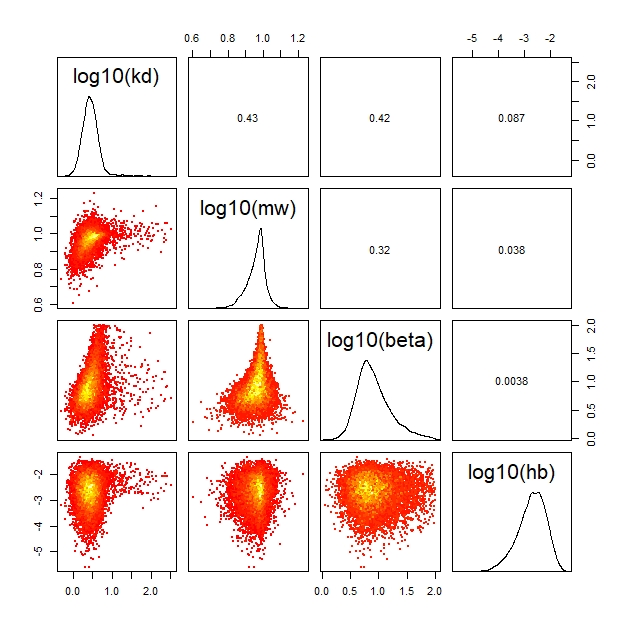

Supplement: Supplementary file 3 — Supporting information. [file ETC-41-1732-s002.zip › morse/Oncorhynchus mykiss/IT_MCMC_pairs_log10_report.jpg]

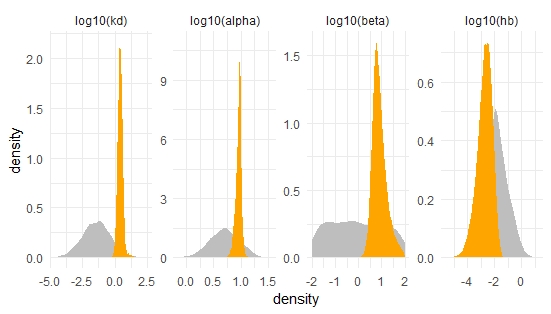

Supplement: Supplementary file 3 — Supporting information. [file ETC-41-1732-s002.zip › morse/Oncorhynchus mykiss/IT_post_prior_distributions.jpg]

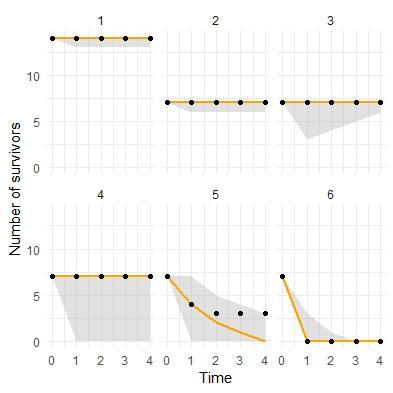

Supplement: Supplementary file 3 — Supporting information. [file ETC-41-1732-s002.zip › morse/Oncorhynchus mykiss/SD_calibration_Nsurv.jpg]

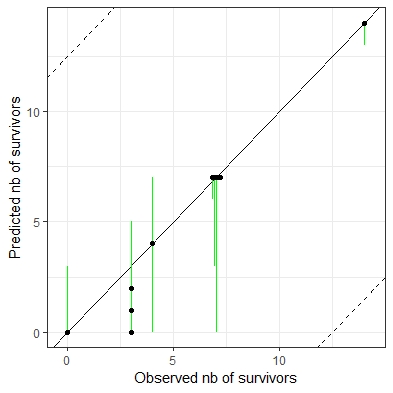

Supplement: Supplementary file 3 — Supporting information. [file ETC-41-1732-s002.zip › morse/Oncorhynchus mykiss/SD_calibration_ppc.jpg]

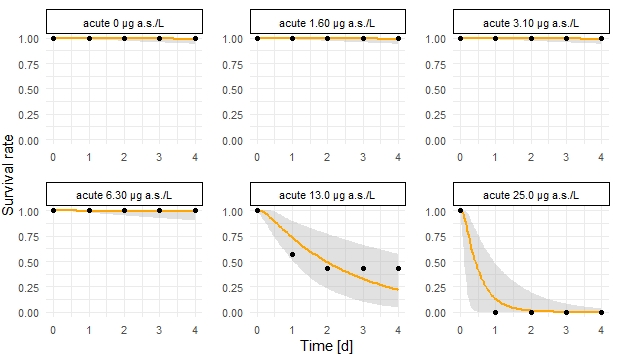

Supplement: Supplementary file 3 — Supporting information. [file ETC-41-1732-s002.zip › morse/Oncorhynchus mykiss/SD_calibration_report.jpg]

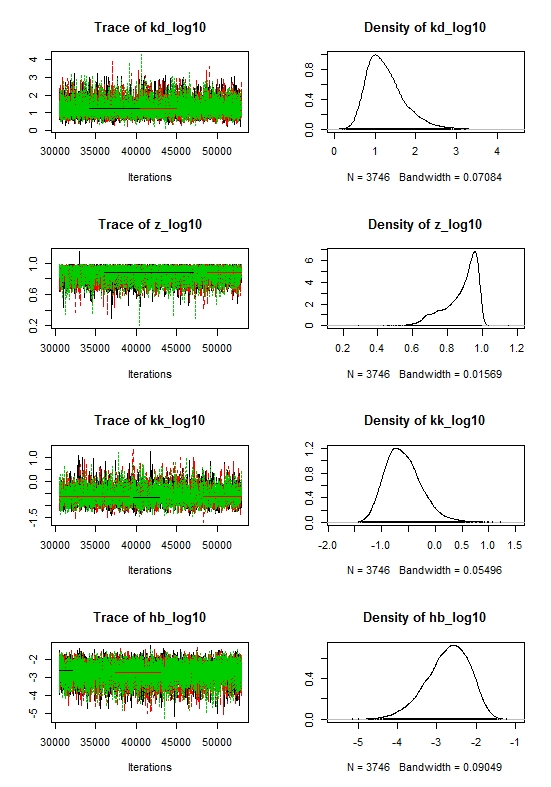

Supplement: Supplementary file 3 — Supporting information. [file ETC-41-1732-s002.zip › morse/Oncorhynchus mykiss/SD_MCMC_Chains.jpg]

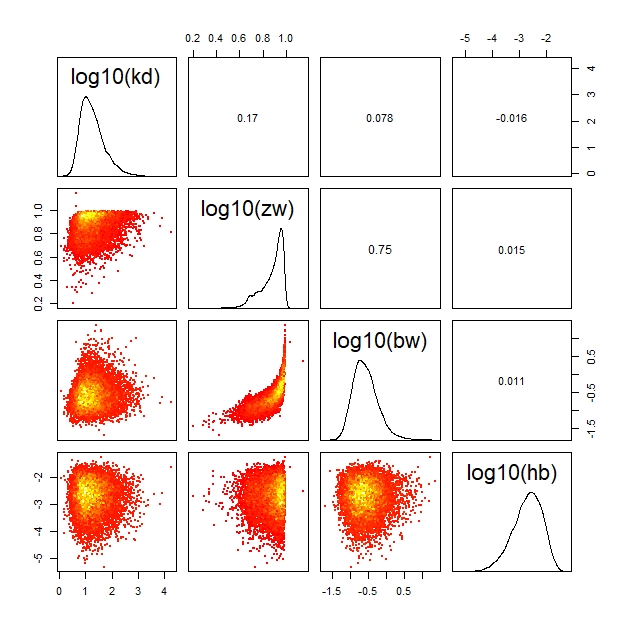

Supplement: Supplementary file 3 — Supporting information. [file ETC-41-1732-s002.zip › morse/Oncorhynchus mykiss/SD_MCMC_pairs_log10_report.jpg]

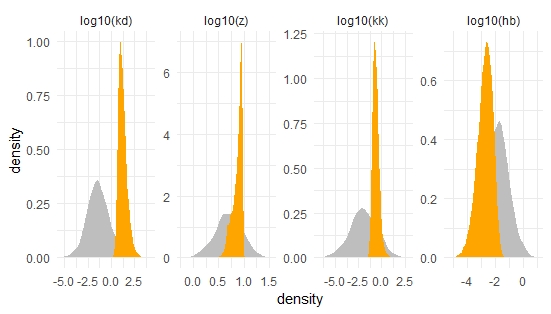

Supplement: Supplementary file 3 — Supporting information. [file ETC-41-1732-s002.zip › morse/Oncorhynchus mykiss/SD_post_prior_distributions.jpg]

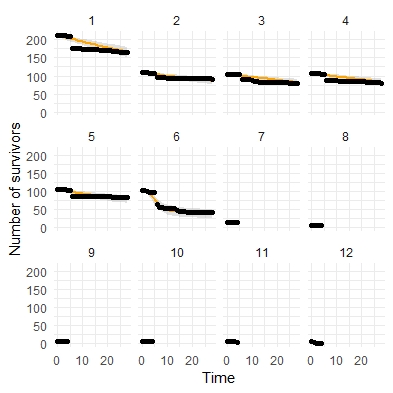

Supplement: Supplementary file 3 — Supporting information. [file ETC-41-1732-s002.zip › morse/Pimephales promelas/IT_calibration_Nsurv.jpg]

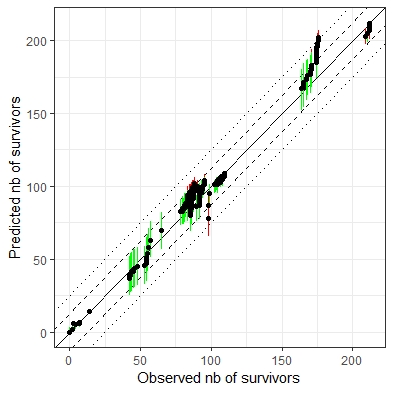

Supplement: Supplementary file 3 — Supporting information. [file ETC-41-1732-s002.zip › morse/Pimephales promelas/IT_calibration_ppc.jpg]

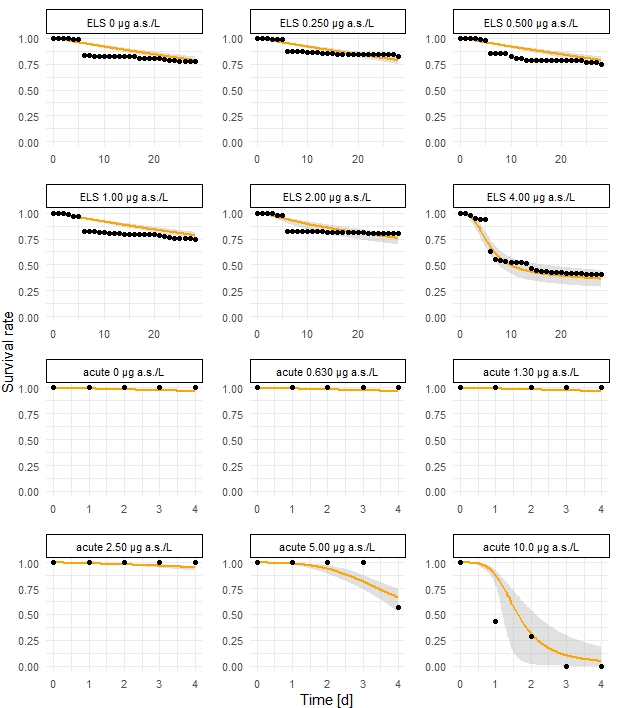

Supplement: Supplementary file 3 — Supporting information. [file ETC-41-1732-s002.zip › morse/Pimephales promelas/IT_calibration_report.jpg]

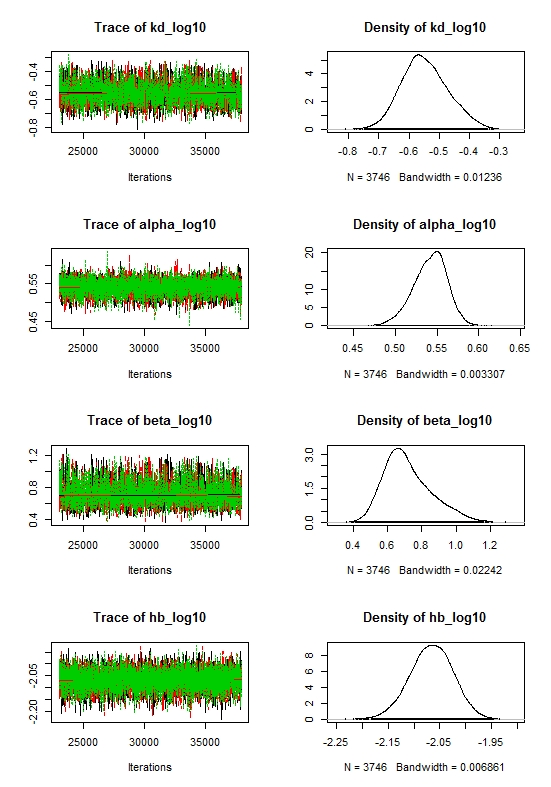

Supplement: Supplementary file 3 — Supporting information. [file ETC-41-1732-s002.zip › morse/Pimephales promelas/IT_MCMC_Chains.jpg]

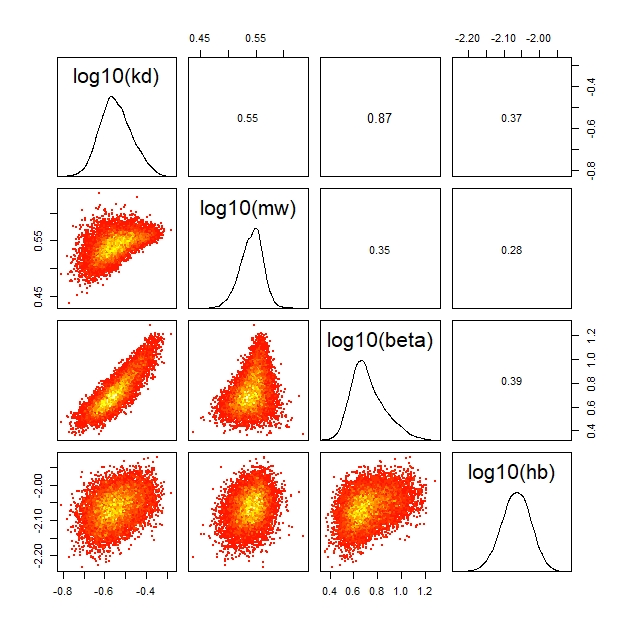

Supplement: Supplementary file 3 — Supporting information. [file ETC-41-1732-s002.zip › morse/Pimephales promelas/IT_MCMC_pairs_log10_report.jpg]

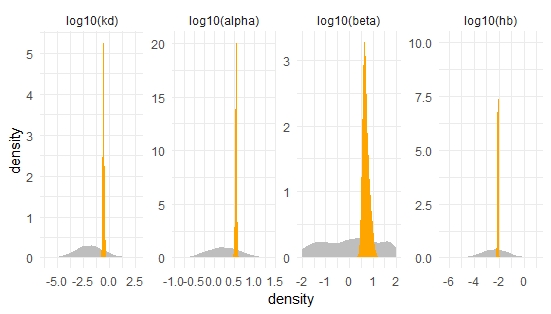

Supplement: Supplementary file 3 — Supporting information. [file ETC-41-1732-s002.zip › morse/Pimephales promelas/IT_post_prior_distributions.jpg]

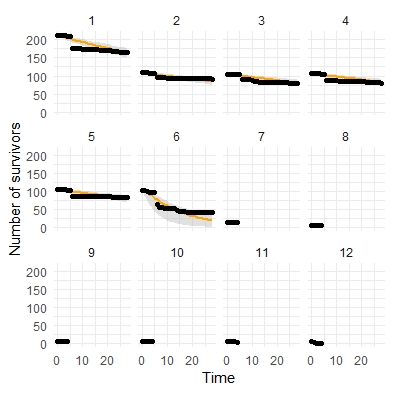

Supplement: Supplementary file 3 — Supporting information. [file ETC-41-1732-s002.zip › morse/Pimephales promelas/SD_calibration_Nsurv.jpg]

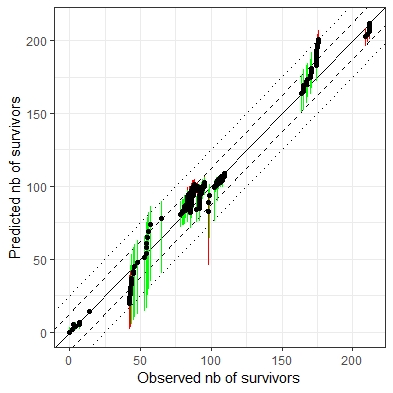

Supplement: Supplementary file 3 — Supporting information. [file ETC-41-1732-s002.zip › morse/Pimephales promelas/SD_calibration_ppc.jpg]

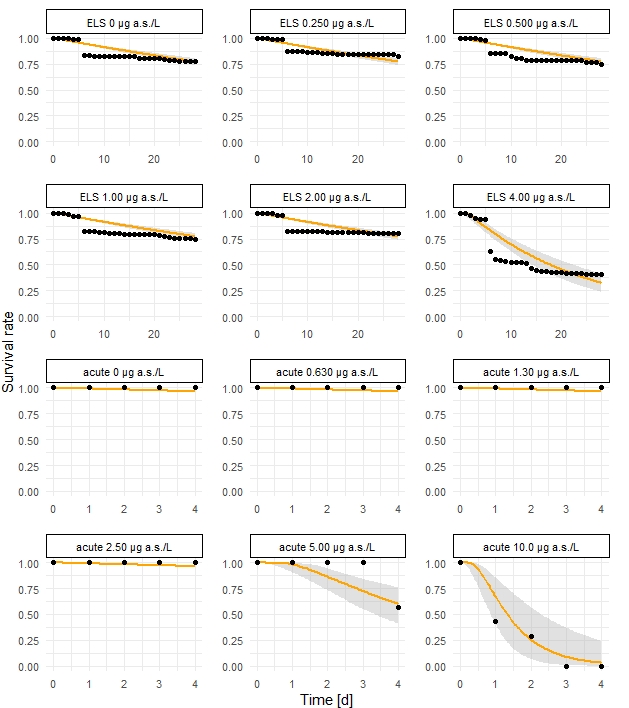

Supplement: Supplementary file 3 — Supporting information. [file ETC-41-1732-s002.zip › morse/Pimephales promelas/SD_calibration_report.jpg]

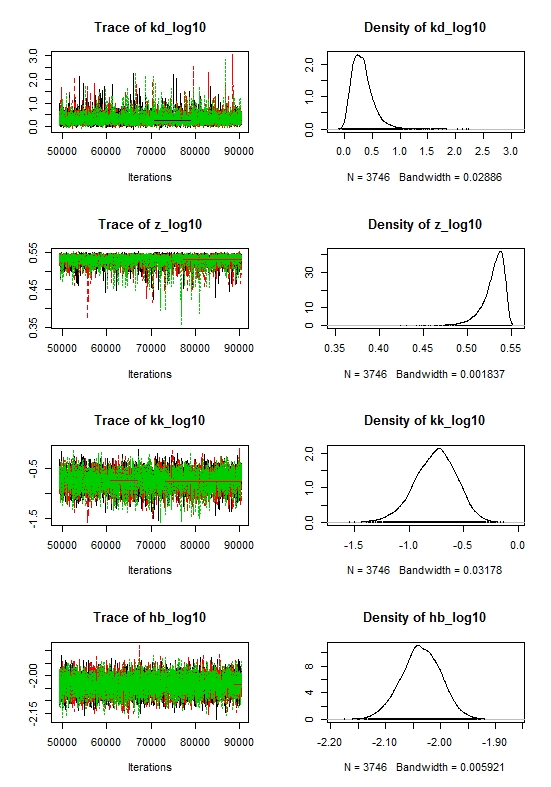

Supplement: Supplementary file 3 — Supporting information. [file ETC-41-1732-s002.zip › morse/Pimephales promelas/SD_MCMC_Chains.jpg]

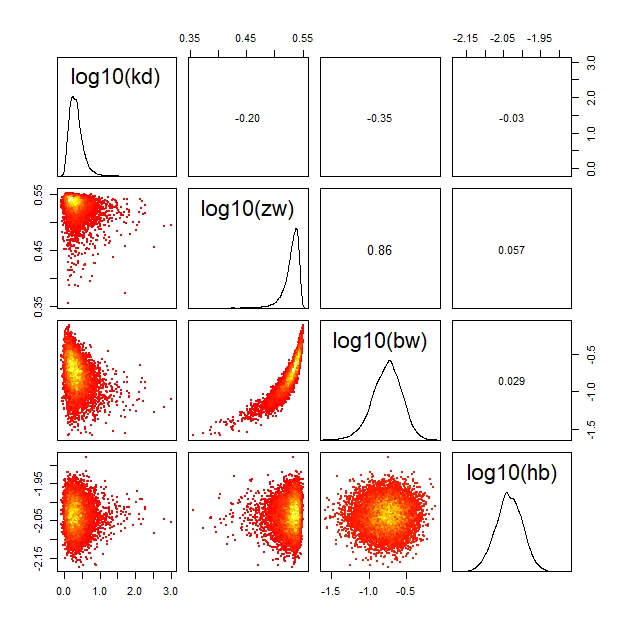

Supplement: Supplementary file 3 — Supporting information. [file ETC-41-1732-s002.zip › morse/Pimephales promelas/SD_MCMC_pairs_log10_report.jpg]

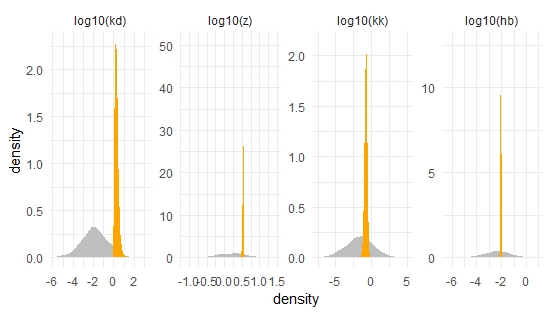

Supplement: Supplementary file 3 — Supporting information. [file ETC-41-1732-s002.zip › morse/Pimephales promelas/SD_post_prior_distributions.jpg]
